# Investigating the Trajectories of Association Between Biomarkers and Cancer-Related Cognitive Impairment in Patients with Breast Cancer: A Systematic Review

**DOI:** 10.3390/cancers17213522

**Published:** 2025-10-31

**Authors:** Angela Boschetti, Laura Danesin, Elisa Bevilacqua, Riccardo Giada, Massimo Gion, Pierfranco Conte, Francesca Burgio

**Affiliations:** IRCCS San Camillo Hospital, 30126 Venice, Italy; laura.danesin@hsancamillo.it (L.D.); elisa.bevilacqua@hsancamillo.it (E.B.); riccardogiada1@gmail.com (R.G.); massimo.gion@hsancamillo.it (M.G.); pierfranco.conte@unipd.it (P.C.); francesca.burgio@hsancamillo.it (F.B.)

**Keywords:** biomarkers, biochemical markers, genetic markers, cancer-related cognitive impairment, breast cancer, objective cognitive tests, self-reported measures

## Abstract

**Simple Summary:**

Cancer-related cognitive impairment (CRCI) is a common and disabling consequence of breast cancer (BC) and its treatments, yet its biological underpinnings remain unclear. This systematic review synthesized evidence from 53 studies examining associations between blood or saliva biomarkers and cognitive outcomes in adults with non-metastatic BC. Most research focused on chemotherapy (ChT), while endocrine therapy (ET) and radiotherapy (RT) were less studied, and immunotherapies were rarely assessed. Assessments were largely conducted post-treatment, with few pre-treatment baselines. Biochemical findings centered on inflammatory cytokines, particularly IL-6 and TNF-α, which were variably associated with cognitive decline depending on timing and assessment type. Other markers, such as CRP, stress-axis hormones, and BDNF, showed mixed results or were associated predominantly with self-reported measures. Genetic studies implicated DNA repair and oxidative stress pathways, but results for APOE, COMT, and BDNF were inconsistent. Overall, biomarker–cognition associations remain heterogeneous, underscoring the need for longitudinal, harmonized, and treatment-specific studies.

**Abstract:**

**Background/Objectives**: Cancer-related cognitive impairment (CRCI) is a frequent and clinically significant consequence of breast cancer (BC) and its treatments. With rapidly evolving therapeutics and a growing body of biomarker research, a BC-specific synthesis is needed. This review aimed to evaluate associations between blood- and saliva-based biomarkers and objective and patient-reported cognitive outcomes in adults with non-metastatic BC, while accounting for treatment modality and assessment timing. **Methods**: This systematic review followed the Preferred Reporting Items for Systematic reviews and Meta-Analyses (PRISMA) guidelines and was pre-registered in the International Prospective Register of Systematic Reviews (PROSPERO, ID: CRD420251079969). PubMed, Embase, and Web of Science were searched for articles published up until April 2025. Eligible studies included adults with BC that investigated associations between blood and/or saliva biomarkers and cognitive outcomes. **Results**: A total of 53 studies met inclusion criteria: 31 examined biochemical biomarkers; 17, genetic; and 5, both. Assessments were predominantly post-treatment. Baseline measures were more infrequent. Chemotherapy (ChT) predominated, while endocrine therapy (ET) and radiotherapy (RT) were variably examined. Targeted therapies and immunotherapies were rarely included. IL-6 and TNF-α were most consistently linked to poorer cognition, although results varied by timing and assessment type. CRP and derived indices showed intermittent associations. Stress-axis markers and BDNF were mainly related to subjective outcomes. Genetic findings implicated DNA repair and oxidative stress pathways, while APOE, COMT, and BDNF results were inconsistent. **Conclusions**: Evidence for biomarker correlates of CRCI in BC is highly heterogeneous. Longitudinal, harmonized, and treatment-specific studies are needed to establish reproducible biomarker panels for risk stratification and targeted intervention.

## 1. Introduction

Breast cancer (BC) is currently the second most common cancer globally, accounting for approximately 2.3 million new cases in 2022, surpassed only by lung cancer [1]. Despite increasing incidence rates over recent decades, BC mortality has steadily declined, leading to improved patient prognosis [2]. This positive trend is consistently attributed to enhanced screening practices, particularly through routine mammography programs that enable earlier detection, as well as heightened public awareness and advances in treatment modalities. Modern therapeutic strategies typically involve multimodal approaches, combining surgery or radiotherapy (RT) with systemic treatments like chemotherapy (ChT), endocrine therapy (ET), or molecular targeted therapies, depending on cancer stage and expected prognosis [3].

Given the improved survival rates, increased attention has been directed towards understanding the long-term sequelae of BC survivorship and assessing potential side effects associated with cancer treatments. Around 70% of cancer patients experience treatment-related symptoms, which may include fatigue, nausea, vomiting, mucositis or stomatitis, fever, hematologic toxicity, and gastrointestinal disturbances. Furthermore, cancer-related cognitive impairment (CRCI) is increasingly recognized as a significant complication of BC and its therapies, affecting 17–78% of patients.

The underlying mechanisms of CRCI are complex and multifactorial. Cancer treatments, particularly ChT and RT, can lead to neurotoxicity by directly damaging neurons or glial cells, or indirectly by altering the neuronal microenvironment [4]. At the molecular level, these treatments can cause oxidative and structural DNA damage [5], contributing to cellular dysfunction. Additionally, they can induce both peripheral and central inflammation, resulting in microglial activation and the release of pro-inflammatory cytokines. These inflammatory mediators may cross the blood–brain barrier, disrupt neuronal signaling, and impair synaptic plasticity, thereby contributing to cognitive decline [6].

Meta-analyses consistently revealed impairments predominantly in visuospatial abilities, language, short-term memory, and fine motor functions among patients undergoing ChT [7,8,9]. ET and RT similarly have been associated with declines in verbal memory, processing speed, language, and executive functioning [10,11,12,13]. Recent evidence suggests that cognitive deficits in BC patients often emerge before the start of systemic therapy and may persist long after treatment completion. A longitudinal study showed that prior to ChT, 21% of patients already demonstrated cognitive dysfunction. Additionally, in the acute interval following ChT, 65% showed cognitive decline, while, long-term evaluations revealed persistent cognitive deficits in 61% of patients after cessation of treatment [14]. Additionally, a recent review showed approximately one-quarter of BC patients exhibited cognitive deficits before initiating any therapy, and many experienced further deterioration as the disease progressed, which in turn, persisted long post-treatment completion, further underscoring that these impairments may extend beyond the direct effects of cancer treatments [15].

Identifying biological mediators of CRCI or associated risk factors is crucial given the multifaceted and persistent nature of cognitive impairment in BC survivors. Uncovering reliable biomarkers could offer valuable insight into biological mechanisms of CRCI, help identify individuals that are potentially at risk of developing CRCI, guide personalized interventions, and provide useful parameters when monitoring therapeutic responses.

Both blood and saliva are valuable biological fluids for collecting biomarkers due to their accessibility and reliability. Blood is one of the most commonly used sources for biomarker analysis because it provides a comprehensive reflection of systemic physiological and pathological processes, allowing the detection of proteins, metabolites, and genetic material with high accuracy [16,17,18]. Moreover, blood collection, although slightly invasive, remains a standardized and efficient procedure for various diagnostic and monitoring purposes [19]. On the other hand, using saliva as a biomarker brings numerous ad-vantages: fast and easy collection, low-cost procedures, and non-invasiveness [20]. In addition, procedures for collection and processing for the determination of several analytes have been standardized [21]. A recent review has also highlighted the potential of saliva to differentiate between BC patients and healthy controls [22]. Despite its ease of collection, fewer studies have focused on saliva biomarkers in relation to CRCI in BC [23,24].

Previous systematic reviews have explored various aspects of biomarkers in relation to CRCI. Castel and colleagues reviewed genetic, inflammatory, and hematologic biomarkers across several cancer types (excluding brain tumors and metastases), identifying associations primarily with genetic polymorphisms (APOE-4 and COMT-Val), inflammatory cytokines (IL-6), anemia, and alterations in hypothalamic–pituitary–adrenal axis hormones [25]. Buskbjerg and colleagues specifically examined genetic risk factors for CRCI in different cancer types, highlighting inconsistent findings regarding APOE4. Other genetic variants identified included those within COMT, DNA repair genes, oxidative stress genes, and genes associated with cancer phenotypes. Notably, genes encoding cytokines and neurotrophic factors generally showed no consistent association [26]. Additionally, Oppegaard and colleagues conducted a broad review on blood-based biomarkers in adults with non-central nervous system cancer. Their synthesis underscored consistent associations of IL-6 and TNF with both subjective and objective cognitive assessments, although they concluded the overall state of biomarker research remained exploratory [27].

Focusing exclusively on BC, Yang and colleagues identified genetic polymorphisms linked to “psycho-neurological” symptoms, such as anxiety, cognitive impairment, depressive symptoms, fatigue, pain, and sleep disturbances. Polymorphisms predominantly related to immune and neuronal pathways were highlighted, with significant variability in their relationship to symptom severity [28]. Furthermore, a recent systematic review investigated biological markers associated with cognitive impairment across treatment trajectory in BC patients treated with ChT. Specifically, blood-based biomarkers, predominantly inflammatory cytokines such as IL-1β, IL-6, IL-17, TNF-α, and CRP, showed consistent associations with cognitive domains including memory, processing speed, and executive functioning. However, the nature of these associations varied significantly depending on the biomarker investigated, timing of measurement, and specific cognitive domains assessed. For example, increased levels of IL-1β and CRP were negatively correlated with processing speed during ChT, whereas some cytokines showed both positive and negative associations with cognitive performance depending on the cognitive domain evaluated. Other biomarkers such as hematologic factors (G-CSF and GM-CSF), metabolic markers (rcSO2), neuronal integrity proteins (tau, Aβ 40, and Aβ 42), and epigenetic changes (e.g., DNA methylation ratios) have also been implicated, particularly in long-term assessments [29].

Despite several prior reviews on biomarkers of CRCI [25,26,27,28,29] important gaps remain. Of note, key aspects need to be further clarified: (1) While many syntheses pooled mixed cancer populations, results focusing on one type of cancer, e.g., BC, are more limited and inconsistent. (2) Prior studies and reviews have mainly emphasized the role of ChT alone on CRCI and related biomarkers, while a comprehensive overview of studies encompassing different therapeutic strategies and their effects at the biological and cognitive levels is still lacking. (3) While some studies show that cancer therapies may highly influence CRCI and related biomarkers, others have highlighted that CRCI may be present even before the administration of pharmacotherapies. However, only a few studies have mapped findings to the timing of assessment of outcome measures (pre-treatment, during treatment, early post-treatment, or long-term survivorship) and investigated how the relationship between CRCI and biomarkers may evolve over time.

Building on prior work, this up-to-date, BC-specific systematic review addresses unmet needs arising from the rapid growth of the literature and the unique biological and therapeutic context of BC. To avoid cross-cancer conflation, we restricted our scope to BC and investigated blood and salivary biomarkers, including genetic variants, while broadening the search to pharmacologic treatment modalities beyond ChT. We examined both objective neuropsychological performance and patient-reported cognitive outcomes to assess convergence versus divergence of results, explicitly noting the timepoint along the cancer trajectory when the cognition was measured, and accounting for key sources of heterogeneity (e.g., cancer stage, molecular subtype, and treatment combinations). Finally, by summarizing the certainty of evidence using the GRADE approach, we aimed to identify the consistency of findings, replications, and which biomarker candidates, if any, warrant prospective validation.

The overarching goals of this review were therefore multiple and complementary. First, we sought to map the current state of evidence linking blood and salivary biomarkers, including genetic variants, with cognitive outcomes in individuals with BC. By doing so, we aimed to determine whether consistent biological signatures of CRCI can be identified across studies, and whether these markers differ according to treatment modality, cancer stage, or individual patient characteristics. Second, we intended to account for the role of different therapeutic strategies beyond ChT in shaping biomarker–cognition associations. This approach acknowledges that CRCI in BC is likely multifactorial, and that mechanisms may differ across treatment classes, requiring a more nuanced understanding. Third, by considering the timing of cognitive assessments along the cancer trajectory (i.e., pre-treatment, during active treatment, or long-term survivorship), we aimed to clarify the temporal dynamics of biomarker influences on cognition. This temporal perspective is crucial to distinguish transient versus persistent impairments, as well as to identify potential windows of vulnerability or resilience. Finally, we systematically examined heterogeneity in the operationalization of cognitive outcomes, explicitly contrasting objective neuropsychological testing with patient-reported cognitive concerns. This dual perspective allows us to assess convergence or divergence between biological correlates of measurable deficits and subjective experiences, both of which have meaningful implications for patient care.

## 2. Materials and Methods

This review was conducted following the Preferred Reporting of Systematic Reviews and Meta-Analyses (PRISMA) statement [30] and was registered in the International Prospective Register of Systematic Reviews (Prospero, ID: CRD420251079969). The PRISMA checklist and abstract checklists are available in the Supplementary Materials (Tables S1 and S2).

### 2.1. Data Sources, Search, and Selection Criteria

A three-step search strategy was undertaken to build the query. Pubmed database was preliminarily searched to capture titles and abstracts, with terms comprising common keywords and free vocabulary. The results were analyzed to validate and build the final query (Table 1), which was adapted to the syntax of three databases (Pubmed, Embase, and Web of Science) that were systematically searched for primary studies published until April 2025 (preliminary search performed on 4 April; last search performed on 28 April 2025). Gray literature was not searched in this review.

Selection criteria for inclusion comprised clinical trials, randomized controlled trials (RCTs) and observational studies, which included adults (>18 years old) who received a diagnosis of BC. Key outcomes for inclusion were the assessment of subjective or objective cognitive functioning and the measurement of blood-based or salivary biomarkers, including biochemical and genetic factors. Exclusion criteria included articles in any language other than English, abstracts, posters, studies that included patients with metastatic cancers or with other types of cancer, studies that did not perform statistical analysis to directly link cognitive functioning and biomarkers, or studies that assessed biomarkers based on other methodologies (e.g., neuroimaging markers). Inclusion and exclusion criteria are also schematically reported in Table 1.

### 2.2. Study Selection

The identified records obtained from the search of each database were imported into Zotero (v. 7.0.16). Duplicate items were identified and removed through the Zotero plugin Zoplicate (Zotero plugin; v 3.0.8). Records were then imported in Rayyan (https://www.rayyan.ai/) [31], an online tool used to perform title and abstract screening. For study selection through abstract screening, three evaluators (AB, LD, and RG) blindly screened the titles and abstracts for eligibility, following the inclusion and exclusion criteria reported in Table 1. Each record was screened by at least two of the three evaluators. The results of the blind selection were then examined and compared. Articles receiving a positive rating from two evaluators were selected for full-text screening. The third reviewer was involved to solve any disagreements between the other two assessors. The three evaluators then independently reviewed the full texts of the publications selected for inclusion, sharing the number of works. If the full text was not publicly available, the corresponding author was contacted by email, and if there was no response in the following two weeks, the study was excluded.

### 2.3. Data Extraction and Synthesis

Data extraction was performed for all full texts that met the inclusion criteria. Relevant data were extracted in a synoptic table created ad hoc for this work, following a modified version of the Population, Intervention, Comparison, and Outcomes (PICOs) guidelines: participants, methodology, outcomes, study design, and results. Relevant outcomes included the assessment of subjective and/or objective cognitive functioning (e.g., administration of neuropsychological tests or self-reported questionnaires) and the measurement of biomarkers. Other relevant outcomes concerned physical efficiency, psychological concerns, daily life activities, or other health-related variables, and were extracted if available and relevant.

Results were charted based on the number of studies investigating different types of biomarkers (inflammation, neuroplasticity, genetic, other) and types of cognitive outcomes (subjective vs. objective cognitive difficulties) to create graphical representations to summarize the results. We assessed certainty of evidence using the Grading of Recommendations, Assessment, Development and Evaluation **(GRADE) approach**, following guidelines for narrative synthesis [32,33], rating each outcome as high, moderate, low, or very low based on risk of bias, inconsistency, indirectness, imprecision, and publication bias. Two reviewers judged certainty independently with consensus by discussion.

### 2.4. Risk of Bias and Methodological Quality Assessment

Risk of bias was evaluated using the Joanna Briggs Institute (JBI) critical appraisal tools, with the checklist corresponding to each study design (analytical cross-sectional study, case–control study, cohort study, quasi-experimental study, and randomized controlled trial checklists) [34]. Afterwards, each item was scored 1 (“Yes”) if the criterion was met and 0 (“No” or “Unclear”) if not met. The total score for each study was converted into a percentage to facilitate comparison, and studies were categorized as low risk (80–100% “Yes”), moderate risk (50–79% “Yes”), or high risk (20–49% “Yes”). JBI tools were selected because they provide validated, design-specific checklists that capture core domains of internal validity, including participant selection, measurement reliability, confounding, and analytical rigor.

## 3. Results and Characteristics of Included Studies

### 3.1. Selected Studies

**Figure 1:**
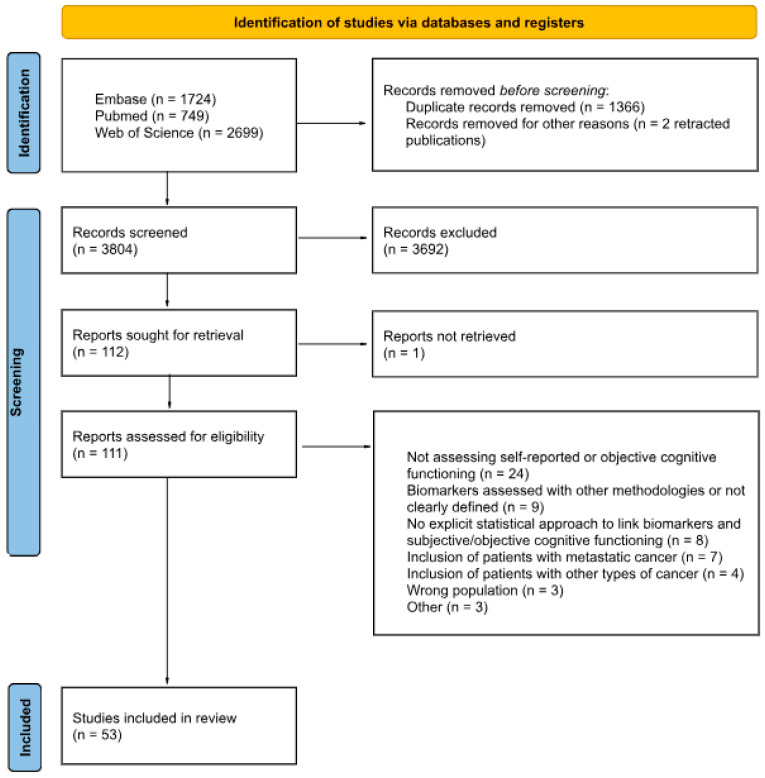
PRISMA flow diagram of study selection. The figure illustrates the process of study identification, screening, eligibility assessment, and final inclusion in the systematic review.

The screening process is summarized in Figure 1. After removing duplicates, 3804 abstracts were screened and 112 full texts were assessed, yielding 53 studies for inclusion. Of these, 31 examined biochemical biomarkers (e.g., inflammatory, stress-related, and neuroendocrine markers), 17 examined genetic biomarkers, and 5 assessed both categories.

Most studies were centered on exploring associations between biomarkers and cognitive function, assessed through both objective neuropsychological testing and self-reported measures. Many studies also sought to characterize specific cognitive domains affected and to determine whether biological changes during or post-treatment related to perceived or objectively measured cognitive performance. Table 2 presents a detailed overview of the specific aims for each study, as well as the study design.

Sample size varied widely, ranging from 8 to 613 participants. All samples for the groups of interest were composed of women with BC, frequently middle-aged, with demographic reporting typically including age and, less consistently, education or ethnicity. Thirty studies included a control group, most often consisting of healthy individuals matched on key demographic variables or another BC patient group. Table 3 provides detailed information on the samples of the included studies, covering demographic and oncological characteristics such as cancer stage, molecular subtype, and biological cancer features.

### 3.2. Assessment Timepoints

Across the included studies, assessment timepoints varied greatly, but most outcome measures were collected after the main treatment phases rather than before. In the studies investigating biochemical biomarkers, only a small number assessed participants prior to surgery (*n* = 4), whereas a larger proportion collected measures post-surgery but pre-pharmacotherapy (*n* = 19) or during pharmacotherapy (*n* = 11). Nonetheless, the most common assessment timepoint was the end of pharmacotherapy (*n* = 28), often to evaluate treatment-related changes. Follow-up assessments were reported in 13 studies, reflecting interest in long-term effects.

**Figure 2:**
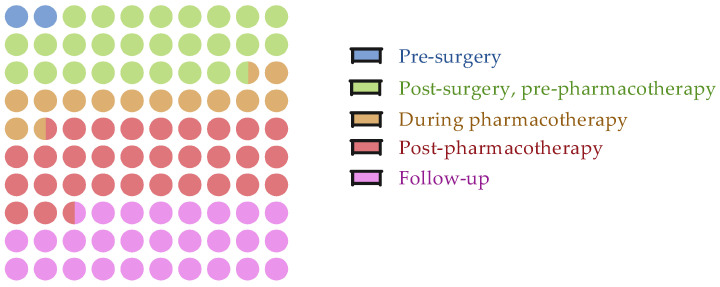
Assessment timepoints across studies. The figure illustrates the distribution of assessment timepoints reported in the included studies. Timepoints are categorized as pre-surgery, post-surgery pre-pharmacotherapy, during pharmacotherapy, post-pharmacotherapy, and follow-up.

In genetic biomarker studies, no pre-surgery assessments of cognitive performance were seen. Most data were gathered post-surgery but pre-pharmacotherapy (*n* = 16) or at the end of pharmacotherapy *(n* = 12), with fewer during treatment (*n* = 7) or in follow-up (*n* = 9). In this case, multiple assessments refer to cognitive outcomes, while genetic outcome measures were mostly collected at one assessment. Figure 2 shows a summary of assessment timepoints. Full details for each study, including the number of assessments to collected outcome measures for both biomarkers and cognitive tests, can be found in the Supplementary Materials (Table S3).

### 3.3. Treatment Regimes

**Figure 3:**
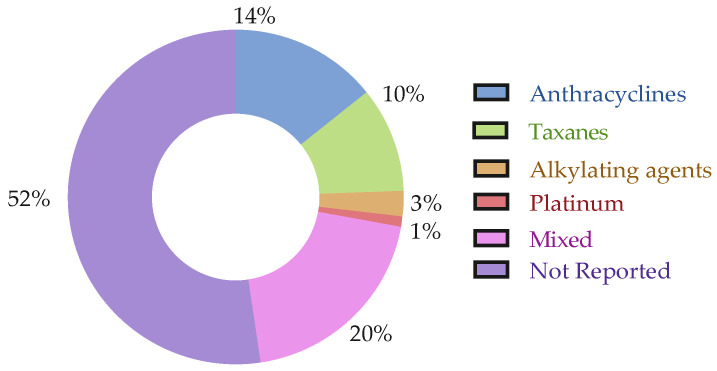
Types of ChT reported across studies. The figure displays the distribution of ChT regimens among the included studies.

**Figure 4:**
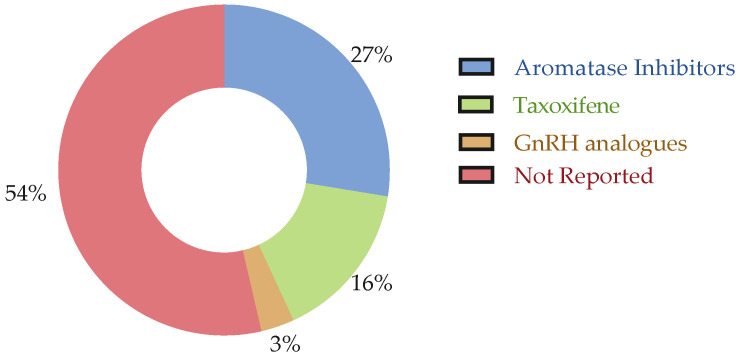
Types of ET reported across studies. The figure displays the distribution of ET regimens among the included studies.

Regarding studies on biochemical biomarkers, patients were reported to have undergone RT (*n* = 22), ET (*n* = 20), ChT (*n* = 35), and, less often, targeted therapy (*n* = 4) (see Figure 3 and Figure 4). Only one study included untreated patients [24]. Two studies clearly reported that patients were ChT-naive and/or RT-naive. Among the studies reporting that patients received ET, more than half (*n* = 12) did not provide treatment details, while the others included patients taking aromatase inhibitors (AI) (*n* = 7) and Tamoxifen (*n* = 7), or involved studies with mixed treatment groups. For studies involving patients who underwent ChT, over one-third (*n* = 14) did not report treatment details. Anthracyclines were the most commonly used treatment (*n* = 17), followed by taxanes (*n* = 15), and other drugs (*n* = 7) or combinations thereof in studies with mixed treatments. Among the studies reporting that patients received targeted therapy, the majority (*n* = 3) used trastuzumab, while only one study involved anti-HER2 therapy. None of the analyzed studies reported information regarding immunotherapy.

On the other hand, regarding studies on genetic biomarkers, patients were reported to have undergone RT (*n* = 9), ET (*n* = 13), and ChT (*n* = 22). Seven studies reported that patients were ChT-naive and/or RT-naive. Among the studies reporting that patients received ET, almost half (*n* = 6) did not provide treatment details, while the others included patients taking aromatase inhibitors (AI) (*n* = 6) and Tamoxifen (*n* = 3). For studies involving patients who underwent ChT, half (*n* = 11) did not report treatment details. Anthracyclines and taxanes were the most commonly used treatment (*n* = 12 and *n* = 12, respectively), followed by other drugs (*n* = 11) or combinations thereof in studies with mixed treatments. None of the analyzed studies reported information regarding immunotherapy or targeted therapy. Full details regarding cancer treatment can be found in the Supplementary Materials (Table S4).

### 3.4. Outcome Measures

Most studies (*n* = 31) used a combination of objective and subjective cognitive assessment, with fewer including solely objective measures (*n* = 16) or subjective measures (*n* = 6). Objective testing most often employed standard neuropsychological batteries, particularly the Mini-Mental State Examination (MMSE) [24] and the Cambridge Neuropsychological Test Automated Battery (CANTAB) [24]. Additional tasks targeted specific processes including attention, processing speed, and working memory. Subjective assessment was dominated by the Functional Assessment of Cancer Therapy-Cognitive Function (FACT-Cog) [24]. Other tools included the Prospective and Retrospective Memory Questionnaire (PRMQ) [24] and the Subjective Memory Questionnaire (SMQ) [24]. The cognitive domains most frequently examined included memory (verbal, working, and episodic), attention (sustained and selective), processing speed, and executive function, with some studies also assessing visuospatial abilities and global cognition.

**Figure 5:**
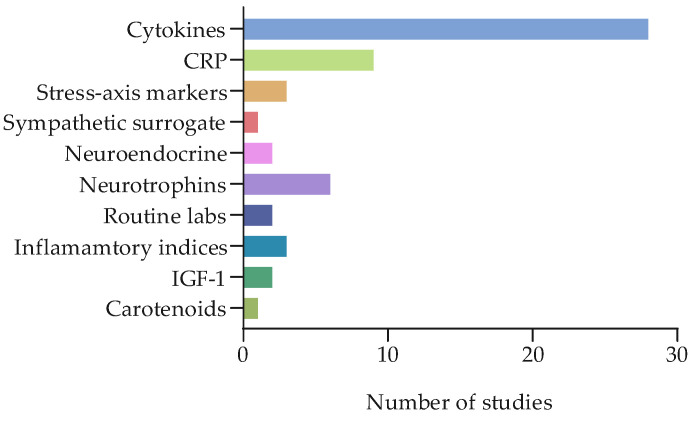
Outcome measures: biochemical biomarkers. The table shows the distribution of biomarkers investigated by the biochemical biomarker studies.

In terms of biomarkers, most studies (28) on biochemical measures evaluated cytokines, including pro-inflammatory markers and anti-inflammatory markers. CRP was measured in nine studies. Stress-axis markers were less common. Endocrine/neuroendocrine and sex-hormone measures (estradiol, progesterone, and testosterone) appeared in two studies. Neurotrophins (BDNF/NGF) were measured in six studies. Among other metabolic markers, two studies evaluated routine basic laboratory tests (hemoglobin, hematocrit, platelet and white-blood-cell counts, albumin, and glucose), while three studies assessed derived inflammatory indices (e.g., NLR, PLR, MLR, GLR, PIV, and SII) (see Figure 5 for details).

**Figure 6:**
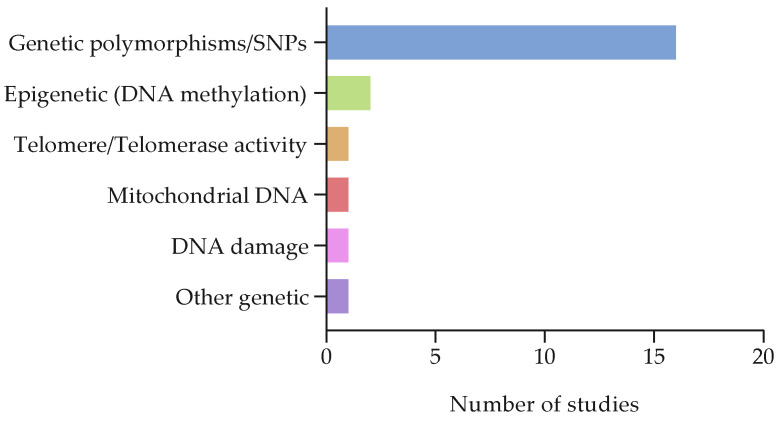
Outcome measures: genetic biomarkers. The table shows the distribution of biomarkers investigated by the genetic biomarker studies.

Across the genetic studies, most examined genetic polymorphisms and SNPs, including variants in COMT, APOE, BDNF, DNMT1, and cytokine-related genes. Epigenetic markers were assessed in two studies, both of which evaluated DNA methylation (e.g., BDNF promoter methylation). Telomeric biomarkers were examined in one study, which assessed leukocyte telomere length and PBMC telomerase activity. Mitochondrial DNA copy number or integrity was reported in one study, while DNA damage was measured in one study, focusing on direct and oxidative damage. One additional study included other isolated genetic measures (see Figure 6).

Full details regarding outcome measures for each study can be found in the Supplementary Materials (Table S5).

## 4. Risk of Bias Assessment and Quality Appraisal

The quality appraisal indicated generally moderate to low risk of bias across the included studies. Most studies met a substantial proportion of JBI checklist criteria, particularly regarding the clarity of inclusion criteria, validity of exposure and outcome measurement, and appropriateness of statistical analyses. However, several studies were rated as moderate risk, largely due to limited reporting of strategies to address confounding factors and incomplete follow-up information. Only one study demonstrated high risk of bias, primarily due to insufficient methodological details. Results are presented in Table 4, Table 5, Table 6, Table 7 and Table 8 depending on study design.

## 5. Relationship Between Biochemical Biomarkers and CRCI

### 5.1. Stress-Axis Biomarkers

Andreano and colleagues demonstrated that acute stress enhanced memory recall for emotional stories in healthy controls but not in BC patients undergoing ET, who exhibited poorer overall retention. Correspondingly, only the control group showed a positive correlation between post-stressor cortisol levels and delayed memory recall, indicating a disrupted cortisol-memory relationship in BC patients [23]. Similarly, Aspelund and colleagues found that patients exhibiting a steeper daily decline in cortisol demonstrated faster processing speed, whereas a flatter cortisol slope was associated with slower cognitive performance. Additionally, a steeper diurnal α-amylase slope, reflecting a pronounced day-to-evening variation, predicted better overall cognitive functioning [24].

### 5.2. Pro-Inflammatory Cytokines

Studies investigating immune-related cytokines and their associations with cognitive performance in BC patients have provided mixed and nuanced findings. Gan and colleagues reported that the pro-inflammatory cytokine IL-1β partially mediated the relationship between psychological distress and self-perceived memory impairment post-ChT [49]. Additionally, post-ChT, higher IL-1β and IL-2 levels were linked to poorer immediate and delayed recall, impaired cognitive flexibility [17], reduced psychomotor speed [60], and poorer global cognitive performance [29]. Interestingly, these cytokines were associated with fewer self-reported cognitive impairments [17,55].

Higher GM-CSF concentrations were associated with slower processing speed post-ChT but fewer subjective cognitive complaints, a pattern similarly observed with IL-2 [17,52]. Another study revealed higher baseline GM-CSF predicted faster reaction times pre-ChT, but six months post-ChT, higher GM-CSF was linked to poorer composite memory. Two years post-treatment, elevated GM-CSF was related to improved psychomotor speed. This same study also reported that baseline elevations in G-CSF were linked to better psychomotor speed, executive functioning, and cognitive flexibility, while higher post-ChT G-CSF correlated with worse reaction times [60].

Among controls and pre-ChT patients, higher IL-5 levels correlated with greater subjective cognitive impairment but not objectively measured deficits [44]. Conversely, during and immediately post-ChT, higher IL-5 was associated with better psychomotor speed and composite memory, yet worse verbal memory two years post-treatment [60].

Higher plasma IL-6 concentrations, which increased during ChT and peaked afterward, were strongly associated with more severe self-reported cognitive impairment; however, Chae and colleagues reported no association between pro-inflammatory cytokines and objectively measured cognitive deficits [41]. In contrast, Duivon and colleagues found that elevated IL-6 levels at diagnosis predicted poorer processing speed and episodic memory two years later [48]. Consistent with these findings, it was observed that higher levels of IL-6 and TNF-α in BC survivors correlated with poorer cognitive performance across various neuropsychological assessments [62].

Another study demonstrated that increasing levels of IL-6 (along with MCP-1) from pre- to post-ChT were associated with declines in executive speed, planning, and verbal fluency. In contrast, higher baseline IL-6 levels pre-ChT predicted better executive functioning and fluency performance post-ChT [53]. Additionally, Lyon and colleagues observed that one year post-ChT, higher IL-6 was linked to improved complex attention [60]. An interaction between IL-6 and TNF-α was also reported, showing that the negative association of TNF-α with memory performance was strongest when IL-6 levels were low and this detrimental relationship diminished as IL-6 levels increased [18].

Lower baseline TNF-α levels predicted deficits in working memory two years later [48]. In another study, elevated TNF-α during ChT was associated with increased self-reported cognitive complaints [55]. Furthermore, from six months to ten years post-ChT, higher TNF-α levels were consistently linked to poorer delayed recall [17] and worse global cognitive performance [68]. Additionally, a prospective study reported that two years post-ChT, elevated TNF-α was associated with slower psychomotor speed [60].

At mid-ChT, higher IL-7 levels were associated with improved composite and visual memory. Six months post-ChT, elevated IL-7 correlated with poorer composite memory but better reaction time, cognitive flexibility, and executive functioning. However, two years post-ChT, higher IL-7 was associated with reduced composite and visual memory performance [60].

Elevated IL-8 levels have been linked to diminished attention, working memory, and processing speed both pre- and post-ChT [16,17]. Greater IL-8 concentrations have also been associated with more self-reported cognitive complaints post-treatment [55] and poorer overall cognitive performance [73]. Interestingly, however, one study observed that higher IL-8 measured a year post-ChT correlated with better executive functioning [60].

In a longitudinal cohort of ChT patients, Lyon and colleagues observed that higher IL-17 levels predicted slower psychomotor speed at baseline, but from the treatment midpoint through 6 months and out to 2 years, elevated IL-17 was linked to faster psychomotor performance, and at 6 months it was also associated with quicker reaction times. At that same 6-month mark, higher IFN-γ corresponded to slower reaction times, yet by 1 year, greater IFN-γ was tied to improved executive functioning. The study further reported that elevated IL-12p70 at the ChT midpoint was related to poorer psychomotor speed, whereas at 6 months higher IL-12p70 was linked to better composite memory [60].

Given the abundance and complexity of findings on pro-inflammatory cytokines, Table 9 synthesizes results, with a specific focus on assessment timepoints and treatment type.

### 5.3. Anti-Inflammatory Cytokines

Elevated levels of anti-inflammatory cytokines IL-4 and IL-10 have been linked to stronger or faster cognitive performance and to longitudinal improvements from pre- to post-ChT in domains such as attention, working memory, processing speed, and executive function [16,60]. Conversely, among BC survivors assessed 6 months to 10 years post-ChT, higher IL-10 has been tied to poorer perceived cognition and weaker objective outcomes, including reduced verbal fluency and impaired executive functioning [17,60,62]. Greater IL-4 concentrations have similarly been associated with slower processing speed and lower global cognition [68], while elevated IL-13 has been connected to diminished verbal fluency [17].

In a separate study, both healthy controls and patients assessed pre-ChT showed that higher IL-10 levels correlated with better self-reported cognition, whereas elevated IL-13 predicted poorer self-reported cognition without corresponding objective deficits [44]. Because cytokine profiles did not differ among patients who went on to receive ChT, those who did not, and healthy controls, these patterns likely reflect an underlying baseline immune environment rather than treatment effects, suggesting that the cognitive difficulties observed in newly diagnosed BC patients may stem more from the disease itself than from impending ChT.

### 5.4. TNF Pathway Markers

Elevated markers of the TNF pathway, specifically sTNFRI and sTNFRII, measured post-ChT have been tied to poorer attention, working memory, and processing speed, while higher pre-treatment sTNFRI predicted worse cognitive performance over time [16]. In another cohort of BC patients who received ChT followed by ET, higher baseline sTNF-RII was associated with greater memory complaints after adjustment for major covariates, but this link vanished once fatigue was taken into account; moreover, reductions in sTNF-RII over time paralleled improvements in self-reported memory [50].

### 5.5. Other Inflammatory Markers

High CRP levels at baseline were associated with impairment in episodic memory, processing speed, and global cognition two years later in BC patients who underwent ChT [48]. At 12–18 months post-ChT, patients with cognitive impairment displayed CRP levels that were nearly twice as high [39]. In addition, higher CRP levels were linked to a stronger attentional bias toward sad faces [38].

SII substantially mediated the relationship between distress and self-reported prospective memory post-ChT [49], while NLR levels increased in patients post-ChT with cognitive impairment compared to those post-ChT without cognitive impairment, and correlated with subjective cognitive difficulties as well as global cognition [80]. Additionally, in another study investigating a behavioral intervention, both higher PIV and higher MLR tracked closely with self-reported cognitive difficulties [76].

### 5.6. Growth Hormones

At least one year post-ChT, lower IGF-1 levels were associated with poorer global cognition [39]. Furthermore, another study, which implemented a behavioral intervention, found that in the intervention group, compared to the control group, IGF-1 rose significantly from baseline to both 4 weeks and 16 weeks and these increases were positively correlated with better cognitive performance, specifically executive control [63].

### 5.7. Cluster of Differentiation (CD) Markers

Higher levels of CD4 and CD8 were related to poorer neuropsychological performance during cancer treatment, while post-treatment completion, they were no longer correlated [36].

### 5.8. Tumor-Associated Antigens

CA153 levels increased in patients post-ChT with cognitive impairment compared to those post-ChT without cognitive impairment and correlated with subjective cognitive difficulties as well as global cognition. CEA was also correlated with cognitive difficulties (both subjective and objective) [80].

### 5.9. BDNF

Higher plasma BDNF was significantly associated only with self-perceived concentration deficits over time in BC patients undergoing ChT [65]. Another study showed BDNF decreased during ChT and a smaller decline was associated with a lower likelihood of clinically meaningful overall subjective CRCI. Considering prognosis post-ChT, higher absolute BDNF predicted less risk of CRCI [78].

### 5.10. Null Findings

Across studies, numerous biomarkers showed no significant associations with either objective or subjective cognitive outcomes in BC patients. For instance, no significant cortisol changes following stress in BC patients were found [23], biological predictors did not explain verbal memory variance [24], and no relationship between sTNF-RII and either self-reported or objectively measured cognition was seen [40]. Similar null associations were seen in other studies, where inflammation indices alone did not predict subjective nor objective cognitive performance [54,61,71]. Immune cell counts were also unrelated to neuropsychological performance outside active treatment phases [36]. No relationships between CRP, IL-6, or TNF-α and global cognitive scores were also reported [39]. Additionally, multiple other cytokines, including IL-1β, IL-2, IL-12p70, TNF-α, and IL-17A showed no significant links to cognition [41,49,50,60,79,81], while IL-8 associations have been seen to lose significance when analyzed in combined models [48]. No BDNF differences between cognitively impaired and non-impaired patients and no correlations between BDNF and cognition were reported [46,53], as well as no effects of NDE class on cognitive performance [67].

**Figure 7:**
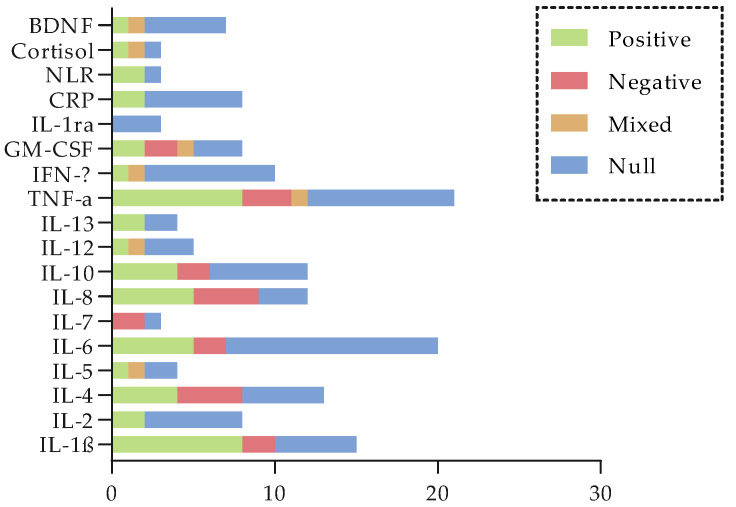
Summary of findings on the relationship between biochemical biomarkers and CRCI. The bar graph shows, across studies, the major non-genetic biomarkers and how their levels are associated with CRCI (consistently positive/direct associations in green; consistently negative/inverse associations in red; null associations in blue and mixed results in orange).

Figure 7 summarizes findings on the relationship between biochemical biomarkers and CRCI while Table 10 displays the relevant GRADE summary of evidence. A summary table (Table A1) can be found in Appendix A, while full results are displayed in the Supplementary Materials (Table S6).

## 6. Genetic Biomarkers

### 6.1. DNA Repair and Oxidative Stress Pathways

Across studies, variants in DNA repair and redox genes showed multiple associations with objective cognition. In a trajectory-based analysis, variation in PARP1 (rs2271347) and ERCC3 (rs4150402) increased the odds of belonging to lower executive function subgroups, while ERCC5 (rs751402; minor T) reduced those odds. In the same cohort, GPX1 (rs1050450; minor A) predicted higher odds of being in the high vs. moderate concentration subgroup, whereas ERCC3 (rs4150407; minor G) was associated with greater odds of low vs. moderate concentration [35].

A larger candidate-gene program similarly linked oxidative stress and DNA repair loci to multiple objective domains. Attention was poorer in carriers of minor alleles in ERCC3 (rs2134794) and ERCC5 (rs873601). Mental flexibility showed effects across ERCC2 (rs13181), ERCC3 (rs4150407 and rs4150477), PARP1 (rs2271347), SEPP1 (rs230819), and SOD1 (rs1041740) variants. Psychomotor speed was associated with CAT (rs511895 and rs769214), ERCC5 (rs11069498, rs751402, and rs873601), and SEPP1 (rs3877899). Concentration was poorer within each SOD2 variant tested (rs4880, rs5746136, rs8031). Importantly, treatment–gene interactions mattered: in patients treated only with AI, ERCC2, ERCC3, or ERCC5, variants bidirectionally influenced concentration, while in those treated with ChT and AI, ERCC3, ERCC5, or PARP1, variants modulated executive function. Moreover, ERCC5 and CAT variants interacted with treatment to influence also verbal memory, visual memory, and visual working memory [57]. By contrast, a separate study found no association between a global oxidative DNA damage marker and any objective cognitive score [47].

### 6.2. Telomere Biology

Objectively measured cognition was seen to correlate more with telomerase activity than with telomere length. Lower telomerase activity predicted worse attention, executive function, and motor speed, while higher DNA damage was related to lower executive function, whereas telomere length was unrelated to any objective domain. Also, none of these biomarkers predicted subjective complaints after covariate adjustment [83].

### 6.3. Cytokine Genotypes

Findings for inflammatory polymorphisms were limited. Subjective memory complaints were marginally worse among TNF-308 GG carriers [37]. However, there were no significant associations of IL6-174 or TNF-308 with either subjective or objective impairment in another cohort [41].

### 6.4. Epigenetics

Epigenome-wide and candidate-site analyses highlighted domain-specific links. In a BDNF/RASA2-focused study, greater methylation at cg20108357 and cg00567892 related to better subjective perceived cognitive function, whereas methylation at BDNF cg21291635 and RASA2 cg20247102 was associated with poorer objective processing speed. Gene-level M-values were not associated with processing speed or perceived cognition [46]. In a separate longitudinal analysis, memory was the only domain with robust epigenetic correlates: 56 CpGs predicted greater objective memory decline after adjustment, while no CpGs predicted psychomotor speed, reaction time, complex attention, or cognitive flexibility [75].

### 6.5. APOE

Associations for APOE ε4 were heterogeneous. Among ChT-treated survivors, ε4 carriage was linked to worse objective processing speed [51]. In a mixed treatment context, ε4 carriers showed poorer verbal learning and memory at two early assessments, greater visual memory decline, and, when combined with AI-only, worse executive function and attention changes. Conversely, under ChT + AI, ε4 status was associated with improved verbal learning and memory over time [56]. Additionally, a large survivorship study reported no APOE-related differences in clinical tests, CANTAB, impairment rates, or FACT-Cog scores [73].

### 6.6. COMT

Multiple COMT loci related to subjective and objective outcomes, with model-dependent effects. In one cohort, rs165599 GA/AA genotypes were protective against objective decline compared to GG. rs737865 genotypes (AG and GG) differed from GG in risk models, but recessive/dominant models did not show consistent effects [45]. Another study found rs737865 AA associated with better event-based prospective memory and rs165599 GG associated with higher odds of objective decline. Model contrasts suggested rs737865 could be protective or risky depending on inheritance assumptions [59]. In longitudinal symptoms, rs4680 (Val158Met) GG genotype showed the most favorable improvement in subjective global cognition, without parallel domain-specific objective advantages [70].

### 6.7. BDNF (Val66Met)

Across multiple datasets, BDNF variants and circulating levels predominantly tracked subjective function. Met carriers had markedly lower odds of subjective global decline and lower odds of deficits in verbal fluency, multitasking, and concentration, with the strongest effects in older participants. There were no objective domain associations in the same cohorts [64,65]. Longitudinally, a smaller reduction in plasma BDNF at end of ChT and higher post-treatment BDNF were protective against subjective global and functional-interference CRCI. Met carriage reduced odds of persistent subjective impairment in mental acuity and multitasking. Neither plasma BDNF nor rs6265 related to objective CRCI [78].

### 6.8. DRD2

Dopaminergic variation showed divergent subjective vs. objective patterns. Subjective perceived cognition improved most for DRD2 rs6277 GG carriers, yet the same genotype was associated with declines in objective visuospatial memory, verbal memory, and executive function [70].

### 6.9. Genome-Wide Association Findings (GWAS)

A case–control GWAS identified two loci, rs76859653 (chr1) and rs78786199 (chr2), that were differentially associated with objective attention, processing speed, and executive function over one year: in controls, minor-allele carriers performed similarly or better than non-carriers, whereas in BC cases, minor-allele carriers performed worse after controlling for baseline scores. Gene-based testing highlighted POC5 as enriched for variants linked to case–control performance differences [66].

### 6.10. Other Polymorphisms

During ChT, carriers of ALDH2 rs671 GG showed worse digit span and verbal fluency. Additional risk was observed for rs886205 GG, rs4648328 CC, and rs4767944 TT. Cognitive indicators declined significantly post-ChT, with the ALDH2 rs671 difference most pronounced [77]. For subjective outcomes, the DNMT1 rs2162560 A allele was protective against concentration difficulties and functional interference, with stronger protection in younger patients (≤51 y) for memory, concentration, and mental acuity. There were no objective associations and no differences by Headminder vs. CANTAB testing [43].

Table 11 displays the relevant GRADE summary of evidence, while a summary table (Table A2) can be found in the Appendix A. Full results on genetic biomarkers are displayed in Supplementary Materials (Table S7).

## 7. Discussion

This systematic review synthesized evidence on biochemical and genetic biomarkers of CRCI in BC. Across the literature, an extensive range of candidate markers has been examined, spanning stress-axis hormones, pro- and anti-inflammatory cytokines, growth factors, and genetic polymorphisms and epigenetic modifications. However, the overall impression is of marked heterogeneity, poor reproducibility, and limited specificity, with findings often varying by study design, timepoint of assessments, and analytic strategy. While some associations may emerge, the field remains far from establishing biological biomarkers of sufficient stability or predictive value to inform clinical application.

Biochemical research to date has focused primarily on inflammatory cytokines, with only a few studies also investigating stress-related hormones and other biochemical markers. Although individual studies demonstrated biologically plausible associations, such as disrupted cortisol-memory coupling in BC patients [24] and links between diurnal cortisol or α-amylase slopes and processing speed [16], null results were equally common. Therefore, even though these findings suggest that hypothalamic–pituitary–adrenal dysregulation may contribute to CRCI, effects appear context-dependent (e.g., related to therapy) and inconsistently reproducible.

It is well established that multiple pharmacological or therapeutic interventions (e.g., RT) can influence a patient’s inflammatory status. Consequently, pro- and anti-inflammatory cytokines, already subject to fluctuations due to the dynamic balance between initiation and resolution of inflammation, may vary even further if the most appropriate timepoint is not taken into account. The cytokine literature is particularly extensive but difficult to reconcile. Pro-inflammatory markers (IL-1β, IL-6, TNF-α, snf IL-8) were each associated with poorer cognitive outcomes in some studies, yet either null or paradoxically protective associations in others. Temporal dynamics further complicate interpretation: several markers (e.g., GM-CSF, IL-5, IL-7, and IL-17) predicted both worse and better cognition depending on treatment phase or follow-up interval. Anti-inflammatory cytokines (IL-4, IL-10, and IL-13) showed similarly inconsistent relationships, sometimes linked to stronger cognition and sometimes to poorer outcomes, depending on study population and analytic approach. These bidirectional or time-sensitive findings may reflect complex immune rebalancing during cancer treatment and survivorship, but they also highlight the limitations of cross-sectional or small-sample designs. In general, there seems to be a tendency towards higher IL-β and IL-6 being associated with cognitive impairment during and after ChT. Similarly, IL-8 shows associations, even before patients are exposed to the toxic effects of ChT. However, the inconsistency of results and the low certainty of the evidence preclude firm mechanistic conclusions.

Across all studies, information regarding potential factors that could further influence the inflammatory status of the patients analyzed is lacking. Although it was often reported that blood samples were collected in the early hours of the day, suggesting adherence to circadian rhythm, a few of the studies provided details on comorbidities, presence of other conditions (e.g., autoimmune diseases), other relevant risk factors (such as smoking or alcohol consumption), or, most importantly, the use of concomitant medications (in addition to ChT or ET examined in the study). These variables can substantially affect the inflammatory balance and, consequently, the measured levels of pro- and anti-inflammatory cytokines. While inflammation is undoubtedly relevant to CRCI pathophysiology, single-point cytokine measurements may be too dynamic to provide clinically useful prognostic information. This is further evidenced by time-dependent, bidirectional associations: higher cytokine concentrations have been linked to both poorer and better cognitive performance, depending on the phase of the cancer trajectory (pre-/mid-/post-ChT and long-term survivorship) [60].

Other biomarker classes, including TNF-receptor signaling, CRP and systemic inflammation indices (NLR, SII, and PIV), IGF-1, CD markers, tumor-associated antigens, and BDNF, were likewise inconsistently related to cognition. Importantly, several large or longitudinal cohorts reported no associations at all between inflammatory markers and objective outcomes [18,41,50,56,57,58]. The view of inconsistent associations between inflammatory markers and cognitive performance or complaints is also strengthened by the fact that, in some cases, studies on the same patient cohorts (e.g., parent and secondary studies on the same sample) yielded opposite or different findings. Thus, although immune dysregulation and systemic inflammation remain plausible mechanistic contributors to CRCI, the current biochemical evidence base lacks consistency, and effect estimates are highly context- and assay-dependent.

Genetic findings similarly reveal intriguing but fragmented patterns. Candidate gene studies of DNA repair and oxidative stress pathways (e.g., ERCC2, ERCC3, ERCC5, PARP1, SOD1/2, SEPP1, CAT, and GPX1) linked minor alleles to multiple objective cognitive domains, sometimes moderated by treatment exposures [63,64]. Yet across cohorts, the direction and magnitude of effects varied, and replication was rare.

Telomere biology yielded some associations, with lower telomerase activity predicting worse executive and attentional function [65], but telomere length itself was not informative. Cytokine polymorphisms (e.g., TNF-308 and IL6-174) showed little or no replicable association with cognition [41,66]. Epigenetic studies highlighted potential CpG sites and BDNF/RASA2 methylation correlates of subjective or domain-specific performance [67,68], but findings were highly exploratory.

Classical risk alleles such as APOE ε4 yielded heterogeneous results: detrimental in some cohorts [69,70], neutral in others [48], and occasionally paradoxically protective depending on treatment context. Likewise, COMT and dopaminergic variants showed mixed associations that were model-dependent and often discordant across subjective versus objective outcomes [64,65,66]. BDNF Val66Met and DRD2 variants were more consistently linked to subjective cognitive difficulties, but rarely to objective impairment [54,55,73,74]. Finally, GWAS have only begun to be applied, with a single study reporting novel loci (chr1 rs76859653 and chr2 rs78786199) and enrichment in POC5 [75], findings which remain unreplicated.

The distribution of assessment timepoints across the included studies has important implications for interpreting biomarker–cognition associations. Most studies, both biochemical and genetic, prioritized post-treatment assessments, particularly post-ChT, with far fewer including pre-surgical baselines. As a result, it is often difficult to disentangle whether observed biomarker–cognition links reflect pre-existing biological vulnerability, acute treatment effects, or longer-term survivorship processes. Only a minority of studies incorporated longitudinal follow-up, further limiting insight into the durability or evolution of biomarker–cognition relationships. Evidence aligned by time frame supports only cautious inferences about trajectories. At baseline, some cohorts suggest that higher inflammatory pattern (e.g., IL-6 and CRP) predicts later deficits in selected domains and that lower TNF-α may presage working-memory problems, but these signals are not consistently reproduced across samples [48]. During ChT, within-person increases in IL-6 and MCP-1 were associated with contemporaneous decrements in executive/fluency and processing speed, while, paradoxically, higher baseline IL-6 related to better post-ChT performance in the same cohort [53]. Mid-treatment associations with specific cytokines (e.g., IL-7 for memory; IL-1β/IL-12 for slower psychomotor speed) were observed in a longitudinal study [60], though directions varied across domains and timepoints. Early post-treatment (0–12 months) studies diverged: pro-inflammatory markers was related to worse self-reported cognition in some work, with no parallel objective effects in others [42]. Over longer-term survivorship (>12–24 months), several markers (e.g., IL-7, IL-8, and GM-CSF) showed time-reversed or domain-specific effects within the same cohort [60], and CRP and TNF-α were intermittently linked with poorer performance or complaints in survivors in individual studies [48,82]. Taken together, available data does not really allow for robust time-anchored comparisons, and it is difficult to establish specific trajectories of biomarker–CRCI associations at this stage.

Treatment heterogeneity further complicates synthesis. ChT, especially anthracyclines and taxanes, was the predominant focus, with RT and ET variably reported, a paucity of data regarding targeted therapy, and entirely absent data regarding immunotherapy, mainly because the PD-1 inhibitor pembrolizumab, recently approved for high-risk, early-stage triple-negative BC based on the phase 3 KEYNOTE-522 trial, was only recently introduced into clinical practice [84,85]. Nonetheless, recent case reports [86,87] highlight the need for greater vigilance about neurological immune-related adverse events associated with immune checkpoint inhibitors, including autoimmune complications. These events may have lasting effects on biomarkers and could contribute to cognitive decline. Notably, treatment details were often under-reported, with many studies failing to specify drug class, regimen, or cumulative dose. This lack of granularity undermines replication and precludes nuanced evaluation of treatment–biomarker interactions, despite growing evidence that regimen type and intensity modulate both cognitive outcomes and biomarker trajectories. The heavy reliance on ChT-based cohorts also limits generalizability to patients treated with newer regimens such as HER2-targeted agents, CDK4/6 inhibitors, or immune checkpoint inhibitors, which are increasingly standard in contemporary BC care. This gap has also been highlighted in a recent umbrella review, which concluded that while ChT and ET are consistently associated with CRCI, evidence for targeted therapies and immunotherapy remains scarce and fragmented [88].

Where available, ChT cohorts more often reported links between inflammatory signaling and objective speed/executive outcomes [16,53], but null and opposite findings were common. ET-specific results appeared in small studies and were not replicated [23] and evidence was insufficient to evaluate consistent biomarker–cognition pattern unique to ET or RT [50,51]. To enable genuine trajectory analyses and modality-specific inferences, future work should standardize pre-treatment baseline and align mid- and post-treatment timepoints. Furthermore, it should be said that even if most studies focused on ChT, these are still patients who also underwent other cancer treatments (e.g., surgery) and can reasonably be assumed to have received peri-operative/supportive medicines. As a result, it is hard to disentangle ChT-specific mechanisms. Precise reporting of co-treatments and their timing is therefore essential, especially considering that inadequate control for these potential confounders was the primary quality concern in risk-of-bias assessments in this review. Additionally, because only a small number of studies assessed biomarkers prior to any treatment (even before surgery), likely reflecting difficulties recruiting patients at that stage, it remains difficult to determine whether biomarker–cognition relationships reflect cancer biology per se or therapy sequelae.

Another notable challenge is the discrepancy between biomarkers associated with subjective versus objective cognitive outcomes. Most studies employed a combination of assessment approaches, while fewer relied solely on objective or subjective measures. These methodological differences likely contribute to the divergence in biomarker associations. Several inflammatory markers (e.g., IL-1β, IL-2, and GM-CSF) were linked to poorer performance on neuropsychological tests but fewer perceived difficulties [36,38,39], whereas BDNF variants, circulating BDNF, and COMT polymorphisms were more robustly tied to self-reported cognitive decline than to objective deficits [54,55,73,74]. Conversely, APOE ε4 and DRD2 variation frequently predicted measurable impairment yet were not mirrored in subjective reports [69,70,73]. Such patterns may be related to the fact that subjective measures are often more sensitive to psychological distress, fatigue, and quality of life, while objective tests may isolate domain-specific deficits but risk underestimating functional relevance. Furthermore, associations with self-reported cognition were more frequent and often attenuated after adjusting for fatigue/mood/sleep [17,42], underscoring the need to measure these mediators explicitly. By contrast, objective processing/executive speed sometimes tracked inflammatory change during treatment [53].

This review has some important limitations that should be acknowledged. First, although conducted as a systematic review, no quantitative meta-analysis was undertaken. We carefully considered whether statistical pooling would be appropriate; however, the included studies displayed substantial clinical and conceptual, rather than statistical, heterogeneity, including a broad array of biomarkers (e.g., CRP, IL-6, IL-8, TNF-α, and cortisol) and genetic polymorphisms (e.g., APOE, COMT, BDNF, and IL-6), each reflecting distinct biological pathways which did not allow for comparable interpretations. Therefore, consistent with PRISMA guidance, a structured narrative synthesis was conducted with the aim to provide a preliminary mapping of the field. Nonetheless, this choice undoubtedly limits the ability to quantify effects. Future systematic reviews and meta-analyses focusing on specific biomarkers, homogeneous cognitive outcomes, and aligned follow-up intervals will be essential to advance quantitative understanding of these associations.

Second, although our search strategy was systematic and clearly reported, it may not have captured all potentially relevant studies. We limited our searches to PubMed, Embase, and Web of Science, without inclusion of more specialized databases such as PsycINFO or trial registries. Given that CRCI spans medical and psychological domains, some relevant studies may have been missed. Moreover, we restricted inclusion to peer-reviewed articles published in English and excluded gray literature, which may have introduced publication and language bias. While these decisions were made for reasons of feasibility and quality control, they nonetheless represent limitations of our review process that should be addressed in future work.

Across biochemical and genetic domains, a consistent theme emerged: putative biomarkers of CRCI are characterized by heterogeneity, poor information, and limited clinical specificity, ultimately leading to poor consistency and reproducibility. Associations often vary by timing (pre-, peri-, or post-treatment), by outcome type (subjective vs. objective), and by analytic model. In some instances, the same biomarker predicts opposite outcomes at different follow-ups. Such inconsistency likely reflects multiple factors: methodological heterogeneity in cognitive testing and biomarker assays, small, underpowered samples, and the biological complexity of CRCI itself. Moreover, the interaction between biological markers and psychophysical factors (e.g., fatigue, distress, and sleep) complicates interpretation of CRCI. Since these mediators may themselves influence both biomarker expression and cognitive outcomes, integrating psychosocial interventions is essential.

From a translational perspective, these findings underscore the current gap between biomarker discovery and clinical application. No biomarker currently demonstrates adequate reproducibility or predictive value to guide individual patient management or support integration into clinical risk stratification, which remains a critical long-term goal. Clinicians should remain alert to cognitive complaints across the cancer trajectory, particularly in patients receiving neurotoxic or inflammatory treatments, and provide early supportive interventions such as cognitive rehabilitation, psychological support, and fatigue management. Awareness of emerging biomarker research may also help clinicians contextualize patient concerns and communicate that CRCI reflects genuine, multifactorial biological processes rather than purely subjective experiences. From a research-clinical integration perspective, clinicians can contribute to advancing the field by supporting longitudinal studies, standardized cognitive testing, and biospecimen collection within clinical trials.

Knowing the state of the art so far and acknowledging that issues with existing evidence are mostly attributable to insufficient reproducibility or predictive stability encourages the need for well-designed prospective studies with standardized timing, harmonized CRCI assessments, and adequately powered cohorts. The ultimate goal is to enable replication and validation across independent samples, with the aim of identifying reliable biomarker panels that could ultimately allow clinicians to find patients at higher risk of CRCI, guide surveillance intensity, and tailor supportive interventions. Given the multifactorial nature of CRCI, future work should also explore multimodal models that integrate biological, psychological, and treatment-related variables. Telemedicine-based rehabilitation, for example, has been shown to mitigate psychological distress and cognitive difficulties in BC patients [89], suggesting that such approaches could indirectly modulate biomarker–cognition relationships and help improve patient outcomes. Bridging these research and clinical domains will be essential to translate biomarker research into meaningful tools that enhance survivorship care and improve quality of life for BC patients.

## 8. Conclusions

In conclusion, this comprehensive review highlights the complex and often inconsistent landscape of biological biomarkers associated with CRCI in BC patients. While numerous candidate biomarkers, including inflammatory cytokines, genetic variants, and neurotrophic factors, show potential links to cognitive outcomes, variability in study methodologies, timing of assessments, and treatment heterogeneity hinder the identification of reliable and clinically useful markers. Future research adopting standardized, longitudinal approaches with larger sample sizes and comprehensive control of confounding factors is essential to clarify the biological mechanisms underlying CRCI and to develop robust biomarkers that can facilitate early identification, personalized interventions, and improved survivorship care.

## Figures and Tables

**Table 1:** Inclusion and exclusion criteria applied for the selection of relevant studies. The table presents the search strategy and predefined eligibility parameters applied to identify and screen studies for inclusion in the systematic review.

Search Strategy	Details
Search query (title/abstract/keywords)	(“Breast Neoplasms” OR “Neoplasm Staging” OR “Breast cancer” OR “Mammary cancer” OR “Breast carcinoma” OR “breast neoplasm *” OR “breast tumor *” OR “breast malignan *”) AND (“Biomarkers” OR “Interleukins” OR “Oxidative Stress” OR “Brain-Derived Neurotrophic Factor” OR “BRCA *” OR “Human Epidermal Growth Factor Receptor 2” OR “HER2” OR “Hormone receptor *” OR “blood biomarker *” OR “serum biomarker *” OR “plasma biomarker *” OR “saliva biomarker *” OR “salivary biomarker *” OR “inflammatory marker *” OR “immune marker *” OR “cytokine *” OR “chemokine *” OR “tumor necrosis factor *” OR “Cortisol” OR “glucocorticoid *” OR “neurotransmitter *” OR “ILs” OR “TNF-alpha” OR “tnfr *” OR “BDNF” OR “SNP”) AND (“cognitive impair *” OR “cognitive deficit *” OR “cognitive difficult *” OR “cognitive dysfunction” OR “Chemo-brain” OR “cancer related cognitive impair *” OR “CRCI” OR “Neurocognitive dysfunction” OR “neuropsychological test *” OR “cognitive test *” OR “cognitive assessment *” OR “cognitive evaluation *” OR “neuropsychology *” OR “neuropsychological assessment *” OR “neuropsychological evaluation *” OR “Memory” OR “Attention” OR “Executive” OR “processing speed *” OR “Fatigue” OR “Cancer-related Fatigue” OR “CRF”)) NOT (“Neurological disorder *” OR “Brain disease *” OR “Neurodegenerative disease *” OR “Dementia” OR “Stroke” OR “Traumatic brain injury” OR “Epilepsy” OR “Multiple sclerosis” OR “Parkinson*” OR “Alzheimer *” OR “Schizophrenia” OR “Bipolar disorder” OR “Psychosis” OR “PTSD” OR “Personality disorder *” OR “Substance abuse” OR “Alcohol abuse” OR “Drug addiction” OR “Opioid use disorder”)
Inclusion criteria	• Population: adult patients with non-metastatic BC. • Outcome: ○ Self-reported or objectively measured cognitive functioning; ○ Biochemical or genetic markers assessed through blood or salivary samples. • Study design: experimental and observational studies.
Exclusion criteria	Population: metastatic cancer, other types of cancer, age < 18 years, not including patients with BC. Outcome: not including measures of self-report or objective cognitive functioning; measuring other types of biomarkers. Study design: not peer-reviewed, gray literature.
Language filter	English
Time filter	4–25 April 2025

* Note: if an article included different types of cancer, including BC, only the results concerning BC were considered. If an article included different types of cancer, including BC, but did not stratify the results based on the cancer type, it was excluded.

**Table 2:** Summary of included studies. The table provides an overview of the studies included in the systematic review, detailing author(s), publication year, study design, and study aims.

Author(s), Year	Study Design	Study Aim(s)
Andreano et al., 2012 [23]	Cross-sectional case–control study	To investigate whether BC-related alterations in the ovarian and glucocorticoid systems interact to influence cognitive function
Aspelund et al., 2024 [24]	Cross-sectional case–control study	To investigate whether pre-treatment cortisol, α-amylase, and stress-related psychiatric symptoms predict CRCI in BC patients
Belcher et al., 2022 [16]	Prospective longitudinal cohort study	To compare serum cytokine and receptor concentrations in BC patients and cancer-free controls, and to examine how these immune markers relate to patients’ cognitive performance
Bender et al., 2018 [35]	Prospective longitudinal cohort study	To describe different patterns of change in cognition from before to during adjuvant therapy in BC patients, and to determine the phenotypic and genetic factors that predict subgroup classification
Boivin et al., 2020 [36]	Prospective longitudinal cohort study	To determine how emotional psychological variables relate to circulating immune cells and to explore whether these immunological markers are associated with neuropsychological performance
Bower et al., 2013 [37]	Cross-sectional study	To test whether promoter polymorphisms are associated with self-reported fatigue in BC survivors and to explore whether these same variants also relate to depressive symptoms, subjective CRCI, and sleep disturbance
Boyle et al., 2017 [38]	Cross-sectional study	To examine whether heightened inflammation in BC survivors is linked to attentional bias towards sad faces
Carlson et al., 2018 [39]	Cross-sectional study	To assess whether biomarkers of vascular aging and ChT-related changes in cerebral blood flow are associated with neuropsychological symptoms in BC survivors
Carrol et al., 2019 * [40]	Cross-sectional study	To investigate whether cellular indicators of biological aging and inflammation are linked to more severe CRCI in BC survivors
Chae et al., 2016 * [41]	Prospective longitudinal cohort study	To determine whether pro-inflammatory cytokine gene variants IL6-174 and TNF-308 are associated with CRCI in early-stage BC patients
Chae et al., 2018 [42]	Prospective longitudinal cohort study	To investigate whether ChT-linked declines in peripheral-blood mitochondrial DNA are associated with the onset and severity of cancer-related fatigue and CRCI in early-stage BC patients
Chan et al., 2019 [43]	Prospective longitudinal cohort study	To investigate whether the DNMT1 gene polymorphism rs2162560 is associated with CRCI in BC patients
Chen et al., 2021 [44]	Cross-sectional study	To explore links between cancer status, ChT exposure, and peripheral cytokine levels with both objective and self-reported CRCI in women with BC, comparing three cross-sectional cohorts
Cheng et al., 2016 [45]	Prospective longitudinal cohort study	To examine whether genetic polymorphisms in COMT, APOE, and BDNF influence susceptibility to CRCI in BC patients
Cho et al., 2024 [46]	Cross-sectional study	To assess whether DNA methylation of the BDNF and RASA2 genes is associated with processing speed and self-reported cognitive function in BC patients
Conroy et al., 2013 [47]	Cross-sectional study	To investigate whether BC patients exhibit greater cognitive dysfunction, neuroimaging abnormalities, and DNA damage compared to than HC, especially with shorter post-ChT intervals
Duivon et al., 2024 [48]	Prospective longitudinal cohort study	To investigate whether higher pre-treatment levels of pro-inflammatory markers predict greater CRCI two years after BC diagnosis
Gan et al., 2025 [49]	Cross-sectional study	To test whether inflammation mediates the link between psychological distress and self-reported CRCI in BC survivors
Ganz et al., 2013 [50]	Prospective longitudinal cohort study	To evaluate whether recent ChT exposure in women with early-stage BC is associated with higher pro-inflammatory cytokine levels and to explore how these inflammatory markers relate to cerebral function and behavioral symptoms
Harrison et al., 2021 [51]	Cross-sectional study	To evaluate how genetic variants relate to brain health in BC patients, as measured by neurocognitive performance and functional connectome analysis
Henneghan et al., 2018 [17]	Cross-sectional study	To investigate whether cytokines predict CRCI in BC survivors 6 months to 10 years after completing ChT, using multivariate non-parametric analyses
Henneghan et al., 2021 [52]	Cross-sectional study	To explore the symptom-cytokine networks and their principal metrics in BC survivors post-ChT
Janelsins et al., 2022 [53]	Prospective longitudinal cohort study	To investigate the inflammatory pathways that underlie CRCI in BC survivors
Jenkins et al., 2016 [54]	Prospective longitudinal cohort study	To evaluate the feasibility and relevance of pre-surgical assessments for CRCI and to determine whether inflammatory markers mediate ChT-related neuropsychological impairments in women with BC
Keetile et al., 2023 [55]	Prospective longitudinal cohort study	To investigate whether neuroinflammation is linked to self-reported CRCI in BC patients
Kesler et al., 2013 [18]	Cross-sectional case–control study	To determine whether ChT-treated BC survivors exhibit hippocampal atrophy and verbal-memory deficits and whether these impairments correlate with circulating levels of pro-inflammatory cytokines
Koleck et al., 2014 [56]	Prospective longitudinal cohort study	To investigate the influence of APOE genotype on cognitive function in postmenopausal women with early-stage BC, both before the start of adjuvant therapy and longitudinally throughout treatment
Koleck et al., 2017 [57]	Cross-sectional study	The current study investigated associations between SNPs in 25 breast cancer-related candidate genes and cognitive performance prior to treatment initiation
Koleck, et al., 2016 [58]	Cross-sectional study	To investigate the associations between genetic polymorphisms and cognitive function in postmenopausal women with BC prior to the initiation of adjuvant therapy
Li et al., 2020 [59]	Prospective longitudinal cohort study	To examine how BDNF, APOE, and COMT polymorphisms influence ChT-induced prospective memory impairments in BC patients, considering varying levels of estrogen and progesterone receptor expression
Lyon et al., 2016 [60]	Prospective longitudinal cohort study	To prospectively assess whether systemic cytokine levels predict CRCI over the two years following diagnosis in women with early-stage BC
Madison et al., 2023 [61]	Prospective longitudinal cohort study (secondary analysis from four distinct studies)	To investigate whether depression accompanied by elevated inflammation or increased intestinal permeability predicts worse subjective and objective CRCI in BC survivors
Mandelblatt et al., 2023 [62]	Cross-sectional study	To assess whether immune activation and inflammatory markers account for CRCI differences between older BC survivors and cancer-free controls
Myers et al., 2022 [63]	Prospective longitudinal sub study nested within a randomized, wait-list controlled trial	To characterize neurotrophic/growth factors and inflammatory biomarkers at baseline, 4 weeks post-intervention, and 16 weeks post-intervention, as well as examine whether shifts in neuroprotective and inflammatory biomarkers track with changes in CRCI
Ng et al., 2016 [64]	Prospective longitudinal cohort study	To evaluate the impact of the BDNF Val66Met polymorphism on CRCI in patients with early-stage BC
Ng et al., 2017 * [65]	Prospective longitudinal cohort study	To investigate how plasma BDNF levels and self-reported CRCI change over the course of ChT in early-stage BC patients and to determine whether plasma BDNF trajectories differ according to BDNF Val66Met genotype
Nudelman et al., 2023 [66]	Prospective longitudinal cohort study	To identify genetic factors that contribute to the risk of cognitive decline in older female BC survivors
Palesh et al., 2025 [67]	Prospective longitudinal cohort study	To investigate the mechanisms driving CRCI in BC survivors, focusing on neuron-derived exosomes as a novel biomarker of neurocognitive decline
Pang et al., 2021 [68]	Prospective longitudinal quasi-experimental cohort study	To assess whether a psychosocial intervention alters serum and buffy-coat biomarkers and cognitive performance in early-stage BC patients with CRCI
Pang et al., 2023 [69]	Cross-sectional study	To determine whether psychological distress contributes to CRCI by modulating circulating levels of IL-1β, TNF-α, and IL-4
Park et al., 2025 [70]	Prospective longitudinal randomized controlled trial	To assess whether genetic variants moderate the effects of Mindfulness-Based Stress Reduction for BC on improvements in cognitive functioning and related symptoms among BC survivors
Patel et al., 2023 [71]	Prospective longitudinal cohort study	To quantify how inflammatory-marker levels shift across specific BC treatment modalities and to test whether treatment-related rises in these markers coincide with worse symptoms, such as greater fatigue, poorer physical functioning, both objective and self-reported CRCI, amplified pain, disrupted sleep, and depressed mood
Toh et al., 2020 [72]	Prospective longitudinal cohort study	To identify distinct cytokine profiles that correspond to differing self-perceived CRCI trajectories in BC survivors
Vardy et al., 2019 * [73]	Cross-sectional study	To examine how neuropsychological performance measured via clinical tests, CANTAB, and FACT-Cog relates to laboratory biomarkers and fMRI findings across three groups
Von Ah et al., 2022 [74]	Prospective longitudinal randomized controlled trial	Exploratory outcomes encompassed objective performance on neuropsychological tests and plasma BDNF concentrations
Yang et al., 2020 [75]	Prospective longitudinal quasi-experimental observational study	To determine whether early-stage BC ChT-induced changes in DNA methylation patterns that persist one year post-treatment initiation are associated with cognitive function
Yao et al., 2022 [76]	Prospective longitudinal randomized controlled trial	To test whether CALM psychotherapy lowers systemic inflammation and, in turn, lessens CRCI in BC patients undergoing ChT
Yao et al., 2023 [77]	Prospective longitudinal cohort study	To determine whether ALDH2 genotyping can identify subgroups of BC patients who are more susceptible to cognitive impairment during ChT
Yap et al., 2020 * [78]	Prospective longitudinal cohort study	To evaluate whether plasma BDNF concentrations and the BDNF Val66Met genotype are linked to CRCI at ChT completion and to persistent or delayed subjective CRCI up to 24 months afterwards in early-stage BC survivors
Yap et al., 2021 [79]	Prospective longitudinal cohort study	To investigate how cytokine and BDNF levels vary across distinct CRCI trajectories in early-stage BC patients
Yu et al., 2022 [80]	Cross-sectional study	To determine whether peripheral blood biomarkers NLR, carcinoembryonic antigen CEA and CA153 are associated with CRCI in early-stage BC patients pre- and post-ChT
Zhao et al., 2020 [81]	Cross-sectional study	To assess pre- versus post-ChT shifts in plasma cytokines and determine how those changes relate to CRCI and quality of life in early-stage BC patients
Zuniga et al., 2018 [82]	Cross-sectional study	To determine whether BC survivors with higher serum total carotenoid levels exhibit better cognitive function and to explore whether elevated carotenoid concentrations correspond to lower systemic inflammatory markers, thereby implicating reduced inflammation as a potential pathway for carotenoid-related cognitive benefits

* Note: studies with asterisk investigated both biochemical and genetic biomarkers.

**Table 3:** Participant characteristics by study and group. This table summarizes, for each included study, the composition of the BC groups and any control groups, along with demographics and cancer-related variables as reported at baseline. Values are presented as means (±SD), ranges, counts, or percentages as provided in the source articles.

Author(s), Year, Country	Group of Interest	Group of Interest: Demographics	Group of Interest: Cancer-Related Information	Control Group	Control Group: Demographics	Control Group: Cancer-Related Information
Andreano et al., 2012 [23] Country: USA	18 BC patients	Age (years, mean): 41.9		20 HC	Age (years, mean): 39.9	/
Aspelund et al., 2024 [24] Country: Iceland	112 BC patients	Age (years, mean ± SD): 61.8 ± 10.7 Education level (%): Primary 16.1; Secondary 32.1; University 47.3	Cancer stage (%): 0 3.6; I 50.9; II 36.6; III 8.9 Molecular type (%): HER-2 positive 7.1; ER positive 90.2; PR positive 71.4	67 HC	Age (years, mean ± SD): 60.9 ± 9.5 Education level (%): Primary 14.9; Secondary 25.4; University 55.2	/
Belcher et al., 2022 [16] Country: USA	519 BC patients	Age (years, mean ± SD): 53.3 ± 10.6 Education level (%): <High school 2.1; High school or GED 22.7; >Some college 75.1; Ethnicity (%): American Indian or Alaska Native 1.2; Asian or Asian American 1.7; Black or African American 8.3; White 88.8	Cancer stage (%): I 26.8; II 49.9; III 18.9; Unknown 4.4	338 HC	Age (years, mean ± SD): 52.8 ± 10.3 Education level (%): <High school 0; High school or GED 11.8; >Some college 88.2; Ethnicity (%): Asian or Asian American 0.9; Black or African American 4.1; White 95	/
Bender et al., 2018 [35] Country: USA	288 early-stage BC (one cohort received ChT followed by anastrozole and the other received anastrozole alone)		BC cohort only (*n* = 261) Cancer stage (%): I 68; IIa 21; IIb 7; IIIa 4	111 HC	/	/
Boivin et al., 2020 [36] Country: USA	20 BC patients in remission	Age (years, mean ± SD): 55.2 ± 12.26 Education level (%): High school or less 40; Some college 20; College degree 30; Post-graduate work or degree 10	In remission	26 BC patients in active treatment	Age (years, mean ± SD): 55.7 ± 10.21 Education level (%): High school or less 23; Some college 31; College degree 19; Post-graduate work or degree 27	Cancer stage (%): 0 4; I 25; II 55; III 15
Bower et al., 2013 [37] Country: USA	171 BC patients	Age (years, mean, range): 51.5 (31–66) Education level (%): High school/some college 15.20; Associate degree/college graduate 54.39; Graduate degree 30.41 Ethnicity (%): White 79.53; Other 20.47	Cancer stage: 0–IIIA	/	/	/
Boyle et al., 2017 [38] Country: USA	91 BC patients	Age (years, mean ± SD): 57 ± 7.85	Cancer stage (%): I 48; II 30; III 22	/	/	/
Carlson et al., 2018 [39] Country: USA	15 BC patients	Age (years, mean ± SD): 64.4 ± 12.3 Education level (n): Post-secondary education 7 Ethnicity (*n*): Caucasian 13	Cancer stage (%): I 6.67; II 53.33; III 40 Biological type: mostly invasive ductal carcinoma	/	/	/
Carrol et al., 2019 * [40] Country: USA	94 BC patients	Age (years, mean ± SD): 56.5 ± 8.1 Education level (%): After college 50; College degree 31; No college degree 19 Ethnicity (%): White, non-Hispanic 80; Hispanic 9; Black 4; Asian 3; Other 4	Cancer stage: 0–IIIA	/	/	/
Chae et al., 2016 * [41] Country: Singapore	125 BC patients	Age (years, mean ± SD): 50.26 ± 8.82 Education level (%): Primary school 16; Secondary school 46.40; Pre-university 20; Graduate/postgraduate 17.60 Ethnicity (%): Chinese 80.80; Malay 10.40; Indian 5.60; Others 3.20	Cancer stage (%): I 17.60; II 52.80; III 29.60	/	/	/
Chae et al., 2018 [42] Country: Singapore	108 BC patients	Age (years, mean ± SD): 52.0 ± 9.2 Ethnicity (%): Chinese 82.4; Malay 9.3; Indian 4.6; Others 3.7	Cancer stage (%): I 12.0; II 66.7; III 21.3	/	/	/
Chan et al., 2019 [43] Country: Singapore	351 BC patients pre-ChT	Age (years, mean ± SD): 51.2 ± 9.1 Education level (%): No education 1.1; Grade school 13.7; High school 45.6; Pre-university college 20.5; College/graduate degree 19.1 Ethnicity (%): Chinese 81.2; Malay 9.7; Indian 5.7; Other 3.4	Cancer stage (%): I 17.4; II 59.8; III 22.8	/	/	/
Chen et al., 2021 [44] Country: Taiwan	106 BC patients divided into two groups: (1) Pre-ChT (70) (2) Post-ChT (36)	Pre-ChT Age (years, mean ± SD): 51.74 ± 11.39 Education (years, mean ± SD): 12.50 ± 3.97 Post-ChT Age (years, mean ± SD): 49.97 ± 10.04 Education (years, mean ± SD): 11.97 ± 3.82	Pre-ChT Cancer stage (%): I 30; II 51.4; III 17.1 Biological type: Invasive breast carcinoma Post-ChT Cancer stage (%): I 30; II 44.4; III 19.4 Biological type: Invasive breast carcinoma	30 HC	Age (years, mean ± SD): 49.97 ± 10.04 Education (years, mean ± SD): 12.50 ± 3.97	/
Cheng et al., 2016 [45] Country: China	80 triple-negative BC patients	Age (years, mean ± SD): 48.48 ± 10.57 Education (years, mean ± SD): 10.09 ± 3.37	Molecular type (%): triple-negative 100 Biological type (%): Non-special type invasive carcinoma 92.5; Special type invasive carcinoma 3.75; Carcinoma in situ 3.75	165 non-triple negative BC patients	Age (years, mean ± SD): 49.39 ± 10.61 Education (years, mean ± SD): 10.08 ± 3.63	Molecular type (%): Non-triple-negative 100 Biological type (%): Non-special type invasive carcinoma 94.55; Carcinoma in situ 4.85; Micro invasive carcinoma 0.61
Cho et al., 2024 [46] Country: USA	102 BC patients	Age (years, mean ± SD): 62.7 ± 7.99 Education (years, mean ± SD): 16.3 ± 2.46 Ethnicity (%): White 88.2; Black 7.8; Other 4	Cancer stage (%): 0 13.7; I 62.7; IIA 14.7; IIB 4.9; IIIA 3.9 Biological type (%): Ductal carcinoma in situ 13.7	/	/	/
Conroy et al., 2013 [47] Country: USA	24 BC patients who received ChT	Age (years, mean ± SD): 57.8 ± 9.6 Education (years, mean ± SD): 15.7 ± 2.1	Cancer stage (%): I 29; IIa 33; IIb 25; IIIa 8; IIIb 4	23 HC	Age (years, mean ± SD): 61.2 ± 9.9 Education (years, mean ± SD): 16.0 ± 2.3	/
Duivon et al., 2024 [48] Country: France	200 BC patients	Age (years, mean ± SD): 54 ± 11 Education (years, mean ± SD): 13.2 ± 2.8	Cancer stage (%): I 43; II 42; III 15; Missing 1 Molecular type (%): HER-2 positive 13; HER-2 negative 87; Missing 0.5	/	/	/
Gan et al., 2025 [49] Country: China	53 BC patients with psychological distress	Age (years, mean ± SD): 51.00 ± 8.06 Education level (%): Primary school 30.19; Junior high school 45.28; University and above 24.53	Cancer stage (%): I 15.09; II 47.17; III 37.74 Molecular type (%): Luminal A 7.55; Luminal B 67.92; HER-2 overexpression 16.98; Triple-negative 7.55 Pathological type (%): Non-invasive carcinoma 3.77; Invasive carcinoma no special type 96.23	51 BC patients without psychological distress	Age (years, mean ± SD): 50.94 ± 6.78 Education level (%): Primary school 21.57; Junior high school 50.98; University and above 27.45	Cancer stage (%): I 7.84; II 54.90; III 37.26 Molecular type (%): Luminal A 3.92; Luminal B 54.90; HER-2 overexpression 31.37; Triple-negative 9.81 Pathological type (%): Non-invasive carcinoma 1.96; Invasive carcinoma no special type 98.04
Ganz et al., 2013 [50] Country: USA	49 BC treated with ChT	Age (years, mean ± SD): 49.9 ± 8.5 Education level (%): Post-high school 16; College 35; Post-college 49 Ethnicity (%): White 84	Cancer stage (%): I 35; II 57; III 8	44 BC not treated with ChT	Age (years, mean ± SD): 52.8 ± 6.7 Education level (%): Post-high school 18; College 32; Post-college 50 Ethnicity (%): White 86	Cancer stage: 0 37; I 56; II 7;
Harrison et al., 2021 [51] Country: USA	83 BC patients divided into two groups: (1) Group treated with ChT (42) (2) Group ChT naïve (41)	Group treated with ChT Age (years, mean ± SD): 55 ± 7 Education (years, mean ± SD): 16 ± 3 Group ChT naïve Age (years, mean ± SD): 59 ± 7 Education (years, mean ± SD): 17 ± 2	Group treated with ChT Cancer stage (%): I 26; II 55; III 19 Group ChT naïve Cancer stage (%): 0 37; I 50; II 13; III 0	53 HC	Age (years, mean ± SD): 55 ± 9 Education (years, mean ± SD): 17 ± 3	/
Henneghan et al., 2018 [17] Country: USA	66 BC patients	Age (years, mean ± SD): 49 ± 8.77 Education (years, mean ± SD): 16.7 ± 2.16 Ethnicity (%): White 93.4; Other 6.6	Cancer stage (%): II-III 81.8 Molecular type (%): ER positive/PR positive 84.8 Biological type (%): Invasive ductal carcinoma 69.7	/	/	/
Henneghan et al., 2021 [52] Country: USA	66 BC patients	Age (years, mean ± SD): 48.44 ± 8.73 Education (years, mean ± SD): 16.7 ± 2.16 Ethnicity (%): White 93.4; African American 1.5; Asian 4.5	Cancer stage (%): I 18.2; II 62.1; III 19.7 Molecular type (%): ER positive/PR positive 84.8; HER-2 positive 39.4 Biological type (%): Invasive ductal carcinoma 69.7; Ductal carcinoma in situ 15.2; Invasive lobular carcinoma 7.6; Multiple 7.6	/	/	/
Janelsins et al., 2022 [53] Country: USA	78 BC patients	Age (years, mean, range): 53.1 (30–73) Education level (%): <High-school diploma or General Educational Development certificate 23.1; > High school 76.9 Ethnicity (%): White 89.7; Other 10.3	Cancer stage (%): I 16.7; II 28.2; III 16.7; Unknown 38.5	78 HC	Age (years, mean, range): 54.4 (28–81) Education level (%): <High-school diploma or General Educational Development certificate 20.5; >High school 79.5 Ethnicity (%): White 98.7; Other 1.3	
Jenkins et al., 2016 [54] Country: UK	8 BC patients receiving ChT	Age (years, mean ± SD): 52.6 ± 3.9 Education level (%): Higher 37.5; Further 25; Secondary 37.5	Cancer stage (n): II 12.5; III 87.5	6 BC patients not receiving ChT	Age (years, mean ± SD): 50.2 ± 2.3 Education level (n): Higher 66.67; Further 16.67; Secondary 16.67	Molecular type: NR Cancer stage (n): I 80; II 20
Keetile et al., 2023 [55] Country: South Africa	113 BC patients divided into two groups: (1) CMF group (53) (2) FAC group (60)	CMF group Age (years, mean): 48.2 Educational level (%): Primary 17; Middle school 47.2; High school 34; Tertiary 1.9 Ethnicity (%): Black 100 FAC group Age (years, mean): 47.1 Educational level (%): Primary 13.3; Middle school 46.7; High school 38.3; Tertiary 1.7 Ethnicity (%): Black 100	CMF group Cancer stage (%): II 41.5; III 58.5 Molecular type: Mostly hormone-independent FAC group Cancer stage (%): II 53.3; III 46.7 Molecular type: Mostly hormone-independent	/	/	/
Kesler et al., 2013 [18] Country: USA	42 BC patients	Age (years, mean ± SD): 54.6 ± 6.5 Education (years, mean ± SD): 16.3 ± 2.6	Cancer stage: I–IIIa	35 HC	Age (years, mean ± SD): 55.5 ± 9.3 Education (years, mean ± SD): 17.0 ± 2.7	/
Koleck et al., 2014 [56] Country: USA	78 BC patients divided into two groups: (1) ChT + Anastrozole (37)–(11) e4 carriers and (26) non-e4 carriers (2) Anastrozole alone (41)–(9) e4 carriers and (32) non-e4 carriers	ChT + Anastrozole Age (years, mean ± SD): e4: 58.64 ± 4.61; non-e4: 58.5 ± 5.67 Education (years, mean ± SD): e4: 16.27 ± 3.35; non-e4: 15.42 ± 2.59 Ethnicity (*n*): e4: 10 Caucasian; non-e4: 25 Caucasian Anastrozole alone Age (years, mean ± SD): e4: 61.56 ± 4.61; non-e4: 61.03 ± 5.61 Education (years, mean ± SD): e4: 15.67 ± 2.96; non-e4: 14.97 ± 3.61 Ethnicity: e4: 9 Caucasian; non-e4 32 Caucasian	ChT + Anastrozole Cancer stage (%): e4: I 72.73; II 27.27; non-e4: I 42.31; IIa 38.46; IIb 11.54; IIIa 7.69 Anastrozole alone Cancer stage: e4: I 88.89; IIa 11.11; non-e4: I 78.13; IIa 21.88	50 HC—16 e4 carriers, 34 non-e4 carriers	Age (years, mean ± SD): e4: 60.25 ± 6.56; non-e4: 57.5 ± 5.59 Education (years, mean ± SD): e4: 15 ± 2.63; non-e4: 14.94 ± 3.43 Ethnicity: e4: 15 Caucasian; non-e4: 33 Caucasian	/
Koleck et al., 2017 [57] Country: USA	138 BC patients divided into two groups: (1) ChT + Anastrozole (55) (2) Anastrozole only (83)	ChT + Anastrozole Age (years, mean ± SD): 58.76 ± 5.46 Education (years, mean ± SD): 15.67 ± 2.78 Ethnicity (%): Caucasian 94.5 Anastrozole alone Age (years, mean ± SD): 62.47 ± 5.96 Education (years, mean ± SD): 14.95 ± 3.05 Ethnicity (%): Caucasian 97.6	ChT + Anastrozole Cancer stage (%): I 44; IIa 34; IIb 12; IIIa 10 Molecular type (%): ER status: Positive 96, Negative 4; PR status: Positive 76, Negative 24; HER-2 status: Positive 19.1, Negative 80.9 Biological type (%): Lymph-node status: Positive 38, Negative 62; Invasive type: Ductal 90, Lobular 10 Anastrozole alone Cancer stage (%): I 81.3; IIa 16.3; IIb 2.5 Molecular type (%): ER status: Positive 100; PR status: Positive 88.8; Negative 11.3; HER-2 status: Positive 5.1; Negative 94.9 Biological type (%): Lymph-node status: Positive 6.3; Negative 93.7; Invasive type: Ductal 79.7; Lobular 17.7; Ductal and lobular 2.5	82 HC	Age (years, mean ± SD): 58.39 ± 5.85 Education (years, mean ± SD): 14.93 ± 2.99 Ethnicity (%): Caucasian 92.7	/
Koleck, et al., 2016 [58] Country: USA	138 BC patients divided into two groups: (1) ChT followe + Anastrozole (55) (2) Anastrozole only (83)	ChT + Anastrozole Age (years, mean ± SD): 58.76 ± 5.47 Education (years, mean ± SD): 15.67 ± 2.78 Ethnicity (%): Caucasian 94.5 Anastrozole alone Age (years, mean ± SD): 62.47 ± 5.96 Education (years, mean ± SD): 14.95 ± 3.06 Ethnicity (%): Caucasian 97.6	ChT + Anastrozole Cancer stage (%): I 45.5; IIa 34.5; IIb 9.1; IIIa 10.9 Anastrozole alone Cancer stage (%): I 83.1; IIa 14.5; IIb 2.4	82 HC	Age (years, mean ± SD): 60.06 ± 6.08 Education (years, mean ± SD): 14.84 ± 2.91 Ethnicity (%): Caucasian 92.6	/
Li et al., 2020 [59] Country: China	232 BC patients divided into two groups: (1) ER negative/PR negative group (113) (2) ER positive/PR positive group (119)	ER negative/PR negative group Age (years, mean ± SD): 48.50 ± 10.70 Education (years, mean ± SD): 9.98 ± 3.66 ER positive/PR positive group Age (years, mean ± SD): 48.92 ± 10.14 Education (years, mean ± SD): 9.74 ± 4.07	ER negative/PR negative group Biological type (%): Non-special type invasive carcinoma of breast 92.04; Special type invasive carcinoma of breast 2.65; Carcinoma in situ 5.31 ER positive/PR positive group Biological type (%): Non-special type invasive carcinoma of breast 94.96; Carcinoma in situ 4.20; Micro invasive carcinoma 0.84			/
Lyon et al., 2016 [60] Country: USA	75 BC patients	Age (years, mean ± SD): 51.52 ± 10.34 Educational level (%): <high school 9; High school 12; >high school 79 Ethnicity (%): Caucasian 71; African American 29	Cancer stage (%): I 27; IIa 41; IIb 21; IIIa 11 Molecular type (%): Luminal A 51; Luminal B 11; Triple negative 29; HER2 positive, ER negative/PR negative 9 Biological type (%): Grade 1 7; Grade 2 37; Grade 3 56	/	/	/
Madison et al., 2023 [61] Country: US	613 BC patients who	Age (years, mean ± SD): 54.4 ± 10.2 Education level (%): High school or less 17; Some college 21; College degree 31; Graduate or professional training 31 Ethnicity (%): White 86; Black 10; Asian 2; Native American 1; Mixed 1	Cancer stage (%): 0 8; I 46; II 39; III 7	/	/	/
Mandelblatt et al., 2023 [62] Country: USA	400 BC patients	Age (years, mean ± SD): 67.8 ± 5.3 Education (years, mean ± SD): 15.5 ± 2.1 Ethnicity (%): Non-White (Black, Hispanic, Asian, other) 17.3; White, non-Hispanic: 82.8	Cancer stage (%): 0 17.4; I 60.9; II 18.2; III 3.6 Molecular type (%): ER positive 18.2; HER-2 positive 3.6	329 HC	Age (years, mean ± SD): 67.6 ± 6.2 Education (years, mean ± SD): 15.6 ± 2.2 Ethnicity (%): Non-White (Black, Hispanic, Asian, other) 16.4; White, non-Hispanic 83.6	/
Myers et al., 2022 [63] Country: USA	15 BC patients undergoing combined exercise and game-based cognitive training	Age (years, mean, range): 54.6 (42–66) Education level (%): High school 7; College 60; Graduate School 33 Ethnicity (%): Black or African American 20; White 80	Cancer stage (%): I 33; II 67; III 7	15 BC wait-list controls	Age (years, mean, range): 55.3 (37–66) Education (%): High School 7; College 80; Graduate School 13 Ethnicity (%): Black or African American 6.7; White 86.7; Two or more races 6.7	Cancer stage (%):I 46; II 46; III 7
Ng et al., 2016 [64] Country: Singapore	145 BC patients	Age (years, mean ± SD): 50.8 + 8.8 Education level (%): Primary school 15.2; Secondary school 48.3; Pre-university 20; Graduate/postgraduate 16.6 Ethnicity (%): Chinese 82.1; Malay 10.3; Indian 4.8; Others 2.8	Cancer stage (%): I 22.1; II 49.7; III 28.3	/	/	/
Ng et al., 2017 * [65] Country: Singapore	51 BC patients	Age (years, mean ± SD): 52.6 ± 9.5 Education level (%): None 2; Primary school 11.8; Secondary school 43.1; Pre-university 13.7; Graduate/postgraduate 29.4 Ethnicity (%): Chinese 78.4; Malay 7.8; Indian 11.8; Other 2%	Cancer stage (%): I 13.7; II 62.8; III 23.5	/	/	/
Nudelman et al., 2023 [66] Country: USA	325 BC patients	Age (years, mean ± SD): 68.2 ± 5.7 Education (years, mean ± SD): 15.3 ± 2.1 Ethnicity (%): White, non-Hispanic 100%	Cancer stage: 0–III	340 HC	Age (years, mean ± SD): 67.9 ± 6.6 Education (years, mean ± SD): 15.7 ± 2.2 Ethnicity (%): White, non-Hispanic 100%	
Palesh et al., 2025 [67] Country: USA	73 BC (behavioral therapy for cancer-related insomnia)	Age (years, mean ± SD): 51 ± 12 Education (years, mean ± SD): 16 ± 3	Cancer stage (%): 0 1; I 32; II 49; III 18 Molecular type (%): ER positive 74; PR positive 53; HER-2 positive 37	55 BC patients (healthy eating education for healthy sleep)	Age (years, mean ± SD): 49 ± 10 Education (years, mean ± SD): 16 ± 3	Cancer stage (%): 0 3; I 32; II 47; III 18 Molecular type: ER positive 72; PR positive 63; HER-2 positive 50
Pang et al., 2021 [68] Country: China	50 BC undergoing the CALM intervention	Age (years, mean ± SD): 52.04 ± 8.55 Education level (%): Primary school 44; Secondary school 52; Technical school 2; University 2	Molecular type (%): HER-2 positive 78; HER-2 negative 22 Biological type (%): Infiltrative 52; Invasive ductal 36; Other 12	78 BC undergoing care as usual	Age (years, mean ± SD): 50.60 ± 6.72 Education level (%): Primary school 44.8; Secondary school 50; Technical school 2.6; University 2.6	Molecular type (%): HER-2 positive 79.5; HER-2 negative 20.5 Biological type (%) Infiltrative 47.4; Invasive ductal 50; Other 2.6
Pang et al., 2023 [69] Country: China	62 BC with psychological distress	Age (years, mean ± SD): 51.37 ± 6.63 Education (years, mean ± SD): 8.58 ± 1.97	Cancer stage (%): I 9.7; II 51.6; III 38.7 Molecular type (%): HER-2 positive 85.5; HER-2 negative 14.5 Biological type (%): Infiltrative 48.4; Invasive ductal 51.6	60 BC without psychological distress	Age (years, mean ± SD): 49.48 ± 7 Education (years, mean ± SD): 9.12 ± 2.28	Cancer stage (%): I 13.3; II 45; III 41.7 Molecular type (%): HER-2 positive 78.3; HER-2 negative 21.7 Biological type (%): Infiltrative 60; Invasive ductal 40
Park et al., 2025 [70] Country: USA	69 BC (Mindfulness-Based Stress Reduction program)	Age (years, mean ± SD): 58.0 ± 11.5 Education level (%): Less than college 23.2; Some college or AA degree 26.1; College degree 24.6; Graduate or professional school 26.1 Ethnicity (%): White 100	Cancer stage: I–III Biological type (%): Lobular carcinoma 4.4; Ductal carcinoma 26.1; Invasive lobular 2.9; Invasive ductal 42; Other/unknown 24.6	59 BC (BC Education Support Program)	Age (years, mean ± SD): 57.2 ± 9.9 Education level (%): Less than college 11.9; Some college or AA degree 32.2; College degree 25.4; Graduate or professional school 30.5 Ethnicity (%): White 100	Cancer stage: I–III Biological type (%): Lobular carcinoma 10.2; Ductal carcinoma 35.6; Invasive Lobular 8.5; Invasive ductal 28.8; Other/Unknown 17
Patel et al., 2023 [71] Country: USA	173 BC patients	Age (years, mean, range): 60 (45–84) Education level (%): Less than High School 6.4; High School Diploma 22; Some College 38.2; Bachelor’s Degree 20.2; Graduate Degree 13.3 Ethnicity (%): White, Non-Hispanic 56.1; White, Hispanic 22; Asian 8.1; Black 6.9; Other 6.9	Cancer Stage (%): 0 15.6; I 43.4; II 31.8; III 9.2 Molecular type (%): Hormone positive, HER-2 negative 63.6; Hormone positive, HER-2 positive 8.7; Hormone positive, HER-2 unknown 10.4; Hormone negative, HER-2 negative (Triple Negative) 8.7; Hormone negative, HER-2 positive 5.2; Hormone negative, HER-2 unknown 1.2; Not tested 2.3	77 HC	Age (years, range): 61 (45–86) Education level (%): Less than High School 1.3; High School Diploma 9.1; Some College 33.8; Bachelor’s Degree 20.8; Graduate Degree 35.1 Ethnicity (%): White, Non-Hispanic 81.8; White, Hispanic 5.2; Asian 2.6; Black 2.6; Other 7.8	
Toh et al., 2020 [72] Country: Singapore	128 BC patients	Age (years, mean ± SD): 51.8 ± 8.9 Education (years, mean ± SD): 10.8 ± 3.4 Education level (%): None 2.3; Primary 11.7; Secondary 48.4; Pre-University 18.8; Graduate and above 18.8 Ethnicity (%): Chinese 82.8; Malay 9.4; Indian 3.9; Others 3.9	Cancer stage (%): I 10.9; II 68.7; III 20.3	/	/	/
Vardy et al., 2019 * [73] Country: Canada	BC patients divided into three groups: (1) ChT + CS + (*n* = 44) (2) ChT + CS − (*n* = 52) (3) ChT − (*n* = 30)	ChT + CS+ Age (years, mean, range): 48.39 (30–60) Education (years, mean, range): 15.5 (9–20) ChT + CS− Age (years, mean, range): 48.39 (29–60) Education (years, mean, range): 15.1 (8–20) ChT− Age (years, mean, range): 54.10 (30–59) Education (years, mean, range): 15.37 (12–20)		/	/	/
Von Ah et al., 2022 [74] Country: USA	19 BC (cognitive training)	Age (years, mean ± SD): 56.3 ± 9.3 Education (years, mean ± SD): 15.2 ± 1.9 Ethnicity (%): Black 36.8; White 63.2	Cancer stage (%): I 26.3; II 47.4; III 21.1; Unsure 5.3	17 BC (active control activities)	Age (years, mean ± SD): 58.8 ± 6.7 Education (years, mean ± SD): 16.5 ± 1.9 Ethnicity (%): Black 41.2; White 58.8	Cancer stage (%): I 23.5; II 58.8; III 11.8; Unsure 5.9
Yang et al., 2020 [75] Country: USA	58 BC patients	Age (years, mean, range): 51.48 ± 10.52 Ethnicity (%): African American 32.8; Caucasian 67.2	Cancer stage (%): I 27.6; IIa 44.8; IIb 19; IIIa 8.6 Molecular type (%): Triple negative 32.8	/	/	/
Yao et al., 2022 [76] Country: China	31 BC (CALM intervention)	Age (years, mean ± SD): 51.65 ± 6.67 Education (years, mean ± SD): 8.36 ± 1.91	Cancer stage (%): I 6.5; II 61.3; III 32.3 Molecular type: ER negative 48.4; ER positive 51.6; PR negative 45.2; PR positive 54.8; HER-2 negative 16.1; HER-2 positive 83.9 Pathological type: Invasive carcinoma no special type 58.1; Invasive carcinoma 41.9	38 BC (care as usual)	Age (years, mean ± SD): 51.65 ± 6.67 Education (years, mean ± SD): 8.36 ± 1.91	Cancer stage (%): I 18.4; II 42.1; III 39.5 Molecular type: ER negative 39.5; ER positive 60.5; PR negative 44.7; PR positive 55.3; HER-2 negative 18.4; HER-2 positive 81.6 Biological type: Invasive carcinoma, no special type: 44.7; Invasive carcinoma: 52.6; Invasive carcinoma special type: 2.6
Yao et al., 2023 [77] Country: China	124 BC patients	Age (years, mean, range): 49.5 ± 10.50 Education (years, mean, range): 10.3 ± 3.86	Molecular type (%): Luminal A 18.55; Luminal B 37.9; HER-2 overexpression 27.42; TNBC 16.13 Biological type (%): Invasive carcinoma no special type 92.74; Invasive Carcinoma Special type 0.81; Noninvasive carcinoma 5.65; Other type 0.81	/	/	/
Yap et al., 2020 * [78] Country: Singapore	174 BC patients	Age (years, mean ± SD): 51.8 ± 8.91 Education level (%): Primary school 11.5; Secondary school 46.6; Pre-university 18.4; Graduate/postgraduate 21.8; Not reported 1.7 Ethnicity (%): Chinese 83.3; Malay 9.8; Indian 4; Others 2.9	Cancer stage (%): I 12.1; II 66.1; III 21.8	/	/	/
Yap et al., 2021 [79] Country: Singapore	136 BC patients	Age (years, mean ± SD): 52.0 ± 8.9 Education level (%): Primary school 12.5; Secondary school 49.3; Pre-university 15.4; Graduate/postgraduate 20.6 Ethnicity (%): Chinese 85.3; Malay 7.4; Indian 3.7; Others 3.7	Cancer (%): I 11; II 66.9; III 22.1	/	/	/
Yu et al., 2022 [80] Country: China	122 BC post-ChT divided into two groups: (1) No cognitive impairment (44) (2) Cognitive impairment (78)	Age (years, mean ± SD): 50.6 ± 6.8 Education (years, mean ± SD): 8.8 ± 2.1	Cancer stage (%): I 11.6; II 48.9; III 39.7 Molecular type (%): HER-2 positive 63.6; HER-2 negative 36.4 Biological type (%): Infiltrative non-specific cancer 52.1; Invasive ductal carcinoma 47.9	66 BC patients pre-ChT	Age (years, mean ± SD): 51.9 ± 7.6 Education (years, mean ± SD): 8.6 ± 2.1	Cancer stage (%): I 15.2; II 48.5; III 36.4 Molecular type (%): HER-2 positive 59.1; HER-2 negative 40.1 Biological type (%): Infiltrative non-specific cancer 51.5; Invasive ductal carcinoma 48.5
Zhao et al., 2020 [81] Country: China	122 BC post-ChT divided into two groups: (1) No cognitive impairment (44) (2) Cognitive impairment (78)	No cognitive impairment Age (years, mean ± SD): 50.15 ± 7.15 Education (years, mean ± SD): 8.77 ± 2.11 Cognitive impairment Age (years, mean ± SD): 50.60 ± 6.71 Education (years, mean ± SD): 8.88 ± 2.16	No cognitive impairment Cancer stage (%): I 11.36; II 47.73; III 40.91 Biological type (%): Infiltrative non-specific cancer 47.73; Invasive ductal carcinoma 52.27 Cognitive impairment Cancer stage (%): I 11.54; II 50; III 38.46 Biological type: Infiltrative non-specific cancer 47.44; Invasive ductal carcinoma 52.56	68 BC patients pre-ChT	Age (years, mean ± SD): 51.55 ± 7.69 Education (years, mean ± SD): 8.67 ± 2.07	Cancer stage (%): I 14.71; II 50; III 35.29 Biological type (%): Infiltrative non-specific cancer 50; Invasive ductal carcinoma 50
Zuniga et al., 2018 [82] Country: USA	29 BC patients	Age (years, mean ± SD): 50.1 ± 10.1 Education level (%): ≥4-year college degree 69; Ethnicity (%): White 69; Black 10.3; Asian 6.9; More than one 13.8	Cancer stage (%): I 27.6; II 37.9; III 20.7; Unknown 3.4 Biological type (%): Ductal carcinoma in situ 10.3	38 HC	Age (years, mean ± SD): 50.8 ± 10.0 Education level (%): ≥4-year college degree 84.2 Ethnicity (%): White 86.8; Asian 2.6; More than one 10.5	

* Note: studies with asterisk investigated both biochemical and genetic biomarkers.

**Table 4:** Risk of bias assessment for cross-sectional studies. The table shows the risk of bias assessment for the studies with a cross-sectional design using the JBI Critical Appraisal Checklist for Analytical Cross-Sectional Studies.

Authors	Q1	Q2	Q3	Q4	Q5	Q6	Q7	Q8	% of Yes	Overall Risk
Andreano et al. (2012) [23]	✓	✗	✓	✓	✓	✗	✓	✓	75%	Moderate risk
Aspelund et al. (2024) [24]	✓	✓	✓	✓	✓	✓	✓	✓	100%	Low risk
Bower et al. (2013) [37]	✓	✗	✓	✓	✓	✓	✓	✓	87%	Low risk
Carlson et al. (2018) [39]	✓	✗	✓	✓	✓	✗	✓	✓	75%	Moderate risk
Carroll et al. (2019) [40]	✓	✗	✓	✓	✓	✓	✓	✓	87%	Low risk
Chen et al. (2021) [44]	✓	✗	✓	✓	✓	✓	✓	✓	87%	Low risk
Cho et al. (2024) [46]	✓	✗	✓	✓	✓	✓	✓	✓	87%	Low risk
Conroy et al. (2013) [47]	✗	✗	✓	✓	?	?	✓	✓	50%	Moderate risk
Gan et al. (2025) [49]	✓	✓	✓	✓	✓	✓	✓	✓	100%	Low risk
Harrison et al. (2021) [51]	✓	✓	✓	✓	✓	✓	✓	✓	100%	Low risk
Henneghan et al. (2018) [17]	✓	✓	✓	✓	✓	✓	✓	✓	100%	Low risk
Henneghan et al. (2021) [52]	✓	✓	✓	✓	✓	?	✓	✓	87%	Low risk
Kesler et al. (2013) [18]	✓	✗	✓	✓	✓	✓	✓	✓	87%	Low risk
Koleck et al. (2016) [58]	✓	✓	✓	✓	✓	✓	✓	✓	100%	Low risk
Koleck et al. (2017) [57]	✓	✓	✓	✓	✓	✓	✓	✓	100%	Low risk
Mandelblatt et al. (2023) [62]	✓	✗	✓	✓	✓	✓	✓	✓	87%	Low risk
Pang et al. (2023) [69]	✓	✓	✓	✓	✓	✓	✓	✓	100%	Low risk
Vardy et al. (2019) [73]	✓	✗	✓	✓	✓	✓	✓	✓	87%	Low risk
Yu et al. (2022) [80]	✓	✗	✓	✓	✓	✗	✓	✓	75%	Moderate risk
Zhao et al. (2020) [81]	✓	✗	✓	✓	✓	✗	✓	✓	75%	Moderate risk
Zuniga et al. (2018) [82]	✓	✓	✓	✓	✓	✓	✓	✓	100%	Low risk

Q1. Were the criteria for inclusion in the sample clearly defined? Q2. Were the study subjects and the setting described in detail? Q3. Was the exposure measured in a valid and reliable way? Q4. Were objective, standard criteria used for measurement of the condition? Q5. Were confounding factors identified? Q6. Were strategies to deal with confounding factors stated? Q7. Were the outcomes measured in a valid and reliable way? Q8. Was appropriate statistical analysis used? Legend: ✓ = Yes; ✗ = No; ? = Unclear.

**Table 5:** Risk of bias assessment for case–control studies. The table shows the risk of bias assessment for the two case–control studies using the JBI Critical Appraisal Checklist for Case–Control Studies.

Authors	Q1	Q2	Q3	Q4	Q5	Q6	Q7	Q8	Q9	Q10	% of Yes	Overall Risk
Nudelman et al. (2023) [66]	✓	✓	✓	✓	✓	✓	✓	✓	✓	✓	100%	Low risk
Koleck et al. (2014) [56]	✓	✓	✓	✓	✓	✓	✓	✓	✓	✓	100%	Low risk

Q1. Were the groups comparable other than the presence of disease in cases or the absence of disease in controls? Q2. Were cases and controls matched appropriately? Q3. Were the same criteria used for identification of cases and controls? Q4. Was exposure measured in a standard, valid and reliable way? Q5. Was exposure measured in the same way for cases and controls? Q6. Were confounding factors identified? Q7. Were strategies to deal with confounding factors stated? Q8. Were outcomes assessed in a standard, valid and reliable way for cases and controls? Q9. Was the exposure period of interest long enough to be meaningful? Q10 Was appropriate statistical analysis used? Legend: ✓ = Yes.

**Table 6:** Risk of bias assessment for cohort studies. The table shows the risk of bias assessment for the studies with cohort design using the JBI Critical Appraisal Checklist for Cohort Studies.

Authors	Q1	Q2	Q3	Q4	Q5	Q6	Q7	Q8	Q9	Q10	Q11	% of Yes	Overall Risk
Bender et al. (2018) [35]	✓	✓	✓	✓	✓	✓	✓	✓	✓	N/A	✓	100%	Low risk
Ng et al. (2016) [64]	✓	✓	✓	✓	✓	✓	✓	✓	✓	N/A	✓	100%	Low risk
Ng et al. (2017) [65]	✓	✓	✓	✓	✓	✓	✓	✓	✓	N/A	✓	100%	Low risk
Belcher et al. (2022) [16]	✓	✓	✓	✓	✓	✓	✓	✓	?	?	✓	82%	Low risk
Boivin et al. (2020) [36]	✓	✓	✓	✓	✓	✓	✓	✓	?	?	✓	82%	Low risk
Chae et al. (2016) [41]	✓	✓	✓	✓	✓	✓	✓	✓	?	?	✓	82%	Low risk
Chae et al. (2018) [42]	✓	✓	✓	✓	✓	✓	✓	✓	✗	N/A	✓	90%	Low risk
Duivon et al. (2024) [48]	✓	✓	✓	✓	✓	✓	✓	✓	✓	✓	✓	100%	Low risk
Toh et al. (2020) [72]	✓	✓	✓	✓	✗	✓	✓	✓	?	✗	✓	73%	Moderate Risk
Yap et al. (2020) [78]	✓	✓	✓	✓	✓	✓	✓	✓	✓	✓	✓	100%	Low risk
Yap et al. (2021) [79]	✓	✓	✓	✓	✓	✓	✓	✓	✓	✓	✓	100%	Low risk
Janelsins et al. (2022) [53]	✓	✓	✓	✗	✗	✓	✓	?	✓	N/A	✓	70%	Moderate Risk
Keetile et al. (2023) [55]	✓	✓	✓	✗	✗	✓	✓	✓	✓	N/A	✓	80%	Low Risk
Li et al. (2020) [59]	✓	✓	✓	✗	✗	✓	✓	?	?	N/A	✓	60%	Moderate Risk
Lyon et al. (2016) [60]	✓	✓	✓	✓	✗	✓	✓	?	?	N/A	✓	64%	Moderate Risk
Madison et al. (2023) [61]	✓	✓	✓	✗	✗	✓	✓	✓	✓	N/A	✓	80%	Low Risk
Palesh et al. (2025) [67]	✓	✓	✓	✗	✗	✓	✓	✓	✓	N/A	✓	80%	Low Risk
Pang et al. (2021) [68]	✓	✓	✓	✗	✗	✓	✓	✓	✓	N/A	✓	80%	Low Risk
Patel et al. (2023) [71]	✓	✓	✓	✓	✓	✓	✓	✓	✓	✓	✓	100%	Low risk
Yao et al. (2023) [77]	✓	✓	✓	✗	✗	✗	✓	✗	?	N/A	✓	50%	Moderate Risk
Jenkins et al. (2016) [54]	✓	✓	✓	✓	✓	✓	✓	✓	✓	N/A	✓	100%	Low risk
Ganz et al. (2013) [50]	✓	✓	✓	✓	✓	✓	✓	✓	✓	✓	✓	100%	Low risk
Cheng et al. (2016) [45]	✓	✓	✓	?	✗	✗	✓	✗	?	?	✓	45%	High risk
Chan et al. (2019) [43]	✓	✓	✓	✓	✓	✗	✓	✓	✓	✓	✓	91%	Low risk

Q1. Were the two groups similar and recruited from the same population? Q2. Were the exposures measured similarly to assign people to both exposed and unexposed groups? Q3. Was the exposure measured in a valid and reliable way? Q4. Were confounding factors identified? Q5. Were strategies to deal with confounding factors stated? Q6. Were the groups/participants free of the outcome at the start of the study (or at the moment of exposure)? Q7. Were the outcomes measured in a valid and reliable way? Q8. Was the follow up time reported and sufficient to be long enough for outcomes to occur? Q9. Was follow up complete, and if not, were the reasons to loss to follow up described and explored? Q10. Were strategies to address incomplete follow up utilized? Q11. Was appropriate statistical analysis used? Legend: ✓ = Yes; ✗ = No; ? = Unclear; N/A = Not applicable.

**Table 7:** Risk of bias assessment for quasi-experimental studies. The table shows the risk of bias assessment for the one quasi-experimental study using the JBI Critical Appraisal Checklist for Quasi-Experimental Studies.

Authors	Q1	Q2	Q3	Q4	Q5	Q6	Q7	Q8	Q9	% of Yes	Overall Risk
Yang et al. (2020) [75]	✓	✗	N/A	N/A	✓	✓	✓	✗	✓	71%	Moderate risk

Q1. Is it clear in the study what is the “cause” and what is the “effect” (i.e., there is no confusion about which variable comes first)? Q2. Was there a control group? Q3. Were participants included in any comparisons similar? Q4. Were the participants included in any comparisons receiving similar treatment/care, other than the exposure or intervention of interest? Q5. Were there multiple measurements of the outcome, both pre and post the intervention/exposure? Q6. Were the outcomes of participants included in any comparisons measured in the same way? Q7. Were outcomes measured in a reliable way? Q8. Was follow-up complete and if not, were differences between groups in terms of their follow-up adequately described and analyzed? Q9. Was appropriate statistical analysis used? Legend: ✓ = Yes; ✗ = No; N/A = Not applicable.

**Table 8:** Risk of bias assessment for RCT. The table shows the risk of bias assessment for the RCT using JBI Critical Appraisal Checklist for Randomized-Controlled Trials.

Authors	Q1	Q2	Q3	Q4	Q5	Q6	Q7	Q8	Q9	Q10	Q11	Q12	% of Yes	Overall Risk
Myers (2022) [63]	✓	?	✓	?	?	✓	?	✓	✓	✓	✓	✓	67%	Moderate risk
Park et al. (2025) [70]	✓	?	✓	✗	✗	✓	?	✓	✓	✓	✓	✓	67%	Moderate risk
Von Ah et al. (2022) [74]	✓	✓	✓	?	?	✓	✓	✓	✓	✓	✓	✓	83%	Low risk
Yao et al. (2022) [76]	✓	✓	✗	?	✓	✓	✓	✓	✓	✗	✓	✓	75%	Moderate risk

Q1. Was true randomization used for assignment of participants to treatment groups? Q2. Was allocation to treatment groups concealed? Q3. Were treatment groups similar at the baseline? Q4. Were participants blind to treatment assignment? Q5. Were those delivering the treatment blind to treatment assignment? Q6. Were treatment groups treated identically other than the intervention of interest? Q7. Were outcome assessors blind to treatment assignment? Q8. Were outcomes measured in the same way for treatment groups? Q9. Were outcomes measured in a reliable way? Q10. Was follow up complete and if not, were differences between groups in terms of their follow up adequately described and analyzed? Q11. Were participants analyzed in the groups to which they were randomized? Q12. Was appropriate statistical analysis used? Legend: ✓ = Yes; ✗ = No; ? = Unclear.

**Table 9:** Pro-inflammatory cytokines and CRCI. The table synthesizes findings on pro-inflammatory cytokines across assessment timepoints, providing the type of pharmacotherapy of interest.

Cytokine	Pre-Pharmacotherapy	During Pharmacotherapy	Post-Pharmacotherapy	Long-Term Survivorship
IL- β			Distress → IL-1β → ↑ perceived memory impairment (post-ChT) ↑ IL-1β → ↑ self-reported cognitive complaints; ↓ memory; ↓ cognitive flexibility; ↓ psychomotor speed; ↓ global cognition (post-ChT)	
IL-2			↑ IL-2 → ↑ self-reported cognitive complaints; ↓ immediate/delayed recall; ↓ cognitive flexibility; ↓ psychomotor speed; ↓ global cognition (post-ChT) ↑ IL-2 → ↓ self-reported cognitive complaints (post-ChT)	
IL-5	↑ IL-5 → ↑ self-reported cognitive complaints (pre-ChT)	↑ IL-5 → ↑ psychomotor speed; ↑ memory (during ChT)	↑ IL-5 → ↑ psychomotor speed; ↑ memory (post-ChT)	↑ IL-5 → ↓ memory (2 years post-ChT)
IL-6		↑ IL-6 → ↑ self-reported cognitive complaints (during ChT)	↑ IL-6 → ↑ self-reported cognitive complaints; ↑ objective CRCI (post-ChT) ↑ (baseline) IL-6 → ↓ executive functions; ↑ executive functions (post-ChT)	↑ (baseline) IL-6 → ↓ processing speed; ↓ memory (2 years post-ChT)
TNF-α	↓ TNF-α → ↑ risk of later CRCI	↑ TNF-α → ↑ self-reported cognitive complaints (during ChT)		↑ TNF-α → ↓ psychomotor speed (2 y post-ChT); ↓ delayed recall; ↓ global cognition (6 months–10 year-survivors)
Il-7		IL-7 → ↑ memory (during ChT)		↑ IL-7 → ↓ memory; ↑ reaction time; ↑ executive functions (6 mo post-ChT); ↓ memory (2 years post-ChT)
IL-8	↑ IL-8 → ↓ attention; ↓ working memory; ↓ processing speed (pre-ChT)		↑ IL-8 → ↓ attention; ↓ working memory; ↓ processing speed (post-ChT)	↑ IL-8 → ↑ executive functioning (1 year post-ChT); ↓ overall/global cognition (6 months–10 year-survivors)
Il-17	↑ IL-17 → ↓ psychomotor speed (pre-ChT)	↑ IL-17 → ↑ psychomotor speed (during ChT)	↑ IL-17 → ↑ psychomotor speed (post-ChT)	↑ IL-17 → ↑ psychomotor speed (6 months → 2 years post-ChT); ↓ reaction time (6 months post-ChT)
IFN-γ				↑ IFN-γ → ↑ reaction time (6 months post-ChT)
IL-12p70		IL-12p70 → ↓ psychomotor speed (during ChT)		↑ IL-12p70 → ↑ composite memory (6 months post-ChT)
GM-CSF	↑ GM-CSF → ↓ reaction time (pre-ChT)		↑ GM-CSF → ↓ processing speed; ↓ self-reported cognitive complaints (post-ChT)	↑ GM-CSF → ↓ composite memory (6 months post-ChT); ↑ psychomotor speed (2 years post-ChT)
G-CSF	↑ G-CSF → ↑ psychomotor speed; ↑ executive functions (pre-ChT)		↑ G-CSF → ↑ reaction times (post-ChT)	

Value-based: “Arrows indicate change in the raw metric; for reaction times, ↓ = faster, ↑ = slower; for accuracy/speed composites, ↑ = better, ↓ = worse.” Performance-based: “Arrows indicate performance; ↑ = better, ↓ = worse.”.

**Table 10:** Grade summary on the relationship between biochemical biomarkers and CRCI. The table shows the effect for each biomarker in relation to CRCI, as well as the number of studies and sample size from which the effect was derived, as well as the degree of certainty of evidence.

Outcome	Effect (Summary of Evidence)	Number of Participants (Studies)	Certainty in the Evidence *
IL-1β	Overall, the direction of the effect is inconsistent but tends toward higher IL-1β being associated with cognitive impairment.	~1600 participants (15 studies).	Low ⊕⊕OO (downgraded for serious inconsistency and imprecision. Biological coherence across studies provides minor upgrading).
IL-2	Overall, the body of evidence is predominantly null with a few positive studies and no consistent direction across cognitive domains.	~750 participants (8 studies).	Very low ⊕OOO (downgraded for serious inconsistency and serious imprecision; observational design starts at low and is further downgraded).
IL-4	Evidence is highly mixed: No consistent cognitive domain pattern emerges.	~1700 participants (13 studies).	Very low ⊕OOO (downgraded for serious inconsistency and serious imprecision. Observational designs start at low; no convincing upgrade).
IL-5	Evidence is sparse and inconsistent: No coherent domain pattern.	~250 participants (4 studies).	Very low ⊕OOO (downgraded for serious imprecision and inconsistency).
IL-6	Across the body of evidence, findings are mixed: direction tends toward a relationship between higher IL-6 and worse cognition in several studies, but many analyses show no effect.	~2500 participants (20 studies).	Low ⊕⊕OO (downgraded for inconsistency and imprecision; modest upgrade consideration for biological coherence and replication, but not enough to exceed low).
IL-7	Evidence is very limited and largely non-supportive: no positive studies identified	~66 participants (2 studies).	Very low ⊕OOO (downgraded for serious imprecision and inconsistency/absence of effect; no upgrading factors).
IL-8	Findings are mixed: several studies link higher IL-8 to worse cognition (executive, attention, global), but many analyses are null or opposite.	~1900 (12 studies).	Low ⊕⊕OO (downgraded for inconsistency and imprecision; observational designs).
IL-10	Evidence is inconsistent: direction varies by study (some report worse, others better performance with higher IL-10), and many results are null.	~1800 (12 studies).	Very low ⊕OOO (serious inconsistency and serious imprecision).
IL-12	Evidence is sparse: no consistent domain pattern.	~415 (5 studies).	Very low ⊕OOO (downgraded for serious imprecision and inconsistency; no upgrading factors).
IL-13	Limited but somewhat consistent evidence: positive studies reported higher IL-13 associated with poorer cognition. Additional studies found null results.	~250 (4 studies).	Low ⊕⊕OO (small but directionally consistent evidence of association; downgraded for imprecision but not for inconsistency).
TNF-α/sTNFRI/II	Broad evidence base, but mixed: effects generally suggest higher TNF-α or soluble receptor levels predict worse cognitive performance, though nulls are frequent.	~3100 (21 studies)	Low ⊕⊕OO (downgraded for inconsistency and imprecision; biological coherence prevents downgrading to very low).
IFN-γ	Very limited evidence: no replication of direction across studies.	~1110 (9 studies)	Very low ⊕OOO (downgraded for serious inconsistency and imprecision).
GM-CSF	Evidence is mixed but limited: associations were inconsistent across domains (attention, memory).	~530 (6 studies)	Very low ⊕OOO (downgraded for imprecision and inconsistency; no upgrading factors).
IL-1ra	No study reported a significant or directional association with cognition. All identified studies found null results.	~300 (3 studies)	Very low ⊕OOO (downgraded for serious imprecision; consistent null evidence, but too limited for confidence).
CRP	Evidence is inconsistent: some studies report higher CRP linked to lower attention or executive scores, but most analyses show no association.	~1250 (8 studies)	Low ⊕⊕OO (downgraded for inconsistency and imprecision; modest biological plausibility supports low rating).
NLR	Evidence is mixed, but directionally tends toward higher NLR associated with poorer cognition	~350 (3 studies)	Low ⊕⊕OO (downgraded for imprecision and risk of bias, but not for inconsistency).
Cortisol	Evidence is inconsistent across studies.	~170 (3 studies)	Very low ⊕OOO (downgraded for serious imprecision and inconsistency).
BDNF	Evidence is mixed and inconsistent: the direction of effects (protective vs. detrimental) varied.	~920 (6–7 studies)	Low ⊕⊕OO (downgraded for inconsistency and imprecision, but biological plausibility prevents rating as very low).

* Certainty ratings use standard GRADE symbols: ⊕⊕⊕⊕ = high, ⊕⊕⊕O = moderate, ⊕⊕OO = low, ⊕OOO = very low.

**Table 11:** Grade summary on the relationship between genetic biomarkers and CRCI. The table shows effect for each biomarker in relation to CRCI, as well as the number of studies and sample size from which the effect was derived, as well as the degree of certainty of evidence.

Outcome	Effect	Number of Participants (Studies)	Certainty in the Evidence *
ANKKI polymorphism	Variants associated with reduced odds of CRCI	~130 participants (1 study)	Very low ⊕OOO (downgraded for concerns with risk of bias and imprecision)
ALDH2 polymorphism	Variants associated with increased odds of CRCI	~230 participants (2 studies)	Low ⊕⊕OO (downgraded for concerns with risk of bias)
APOE polymorphism	Evidence is mixed	~890 participants (6 studies)	Very low ⊕OOO (due to inconsistencies in the results and some concerns about the risk of bias)
BDNF polymorphism	Evidence is mixed	~920 participants (6 studies)	Very low ⊕OOO (downgraded for serious inconsistency and concerns about the risk of bias)
CAT polymorphism	Variants associated with reduced odds of CRCI	~140 participants (1 study)	Low ⊕⊕OO (downgraded for imprecision)
CCNBI polymorphism	Variants associated with increased odds of CRCI	~140 participants (1 study)	Low ⊕⊕OO (downgraded for imprecision)
COMT polymorphism	Evidence is mixed	~600 participants (3 studies)	Very low ⊕OOO (due to inconsistencies in the results and some concerns about the risk of bias)
DIAPH3	Variants associated with reduced odds of CRCI	~140 participants (1 study)	Low ⊕⊕OO (downgraded for imprecision)
DNMT1	Variants associated with reduced odds of CRCI	~110 participants (1 study)	Low ⊕⊕OO (downgraded for imprecision)
DRD2 polymorphism	Variants associated with reduced odds of CRCI	~130 participants (1 study)	Very low ⊕OOO (downgraded due to some concerns in the risk of bias and imprecisions)
ERCC2 polymorphism	Mixed results, rs13181 variant associated with increased odds of CRCI, the others with decreased odds	~140 participants (1 study)	Low ⊕⊕OO (downgraded for imprecision)
ERCC3 polymorphism	Mixed results, rs2134794 variant associated with increased odds of CRCI, the others with decreased odds	~430 participants (2 studies)	Very low ⊕OOO (downgraded due to inconsistencies in the results)
ERCC5 polymorphism	Mixed results, rs2296147 variant associated with reduced odds of CRCI, the rs751402 with mixed effects, and the others with increased odds of CRCI	~430 participants (2 studies)	Very low ⊕OOO (downgraded due to inconsistencies in the results)
ESR1	Mixed results on the rs488133 variant, the other yielded null results	~140 participants (1 study)	Very low ⊕OOO (downgraded due to inconsistencies in the results and imprecision)
GPX1 polymorphism	Variants associated with preserved concentration	~300 participants (1 study)	Low ⊕⊕OO (downgraded for imprecision)
GSTM1	Evidence is mixed	~140 participants (1 study)	Very low ⊕OOO (downgraded due to inconsistencies in the results and imprecision)
HMCN1	Variants associated with increased odds of CRCI	~325 participants (1 study)	Low ⊕⊕OO (downgraded for imprecision)
ILB	Null results	~170 participants (1 study)	Low ⊕⊕OO (downgraded for imprecision)
IL6	Evidence is mixed	~300 participants (2 studies)	Very low ⊕OOO (downgraded due to inconsistencies in the results)
MYBL2	Evidence is mixed	~140 participants (1 study)	Very low ⊕OOO (downgraded due to inconsistencies in the results and imprecision)
PARP1	Variants associated with reduced odds of CRCI	~430 participants (2 studies)	Moderate ⊕⊕⊕O (replicated, larger samples)
PGR	Variants associated with reduced odds of CRCI	~140 participants (1 study)	Low ⊕⊕OO (downgraded for imprecision)
SEPP1	Mixed results, rs3877899 variant associated with increased odds of CRCI, while the rs230819 variant was associated with reduced odds	~140 participants (1 study)	Low ⊕⊕OO (downgraded for imprecision)
SOD1	Variants associated with reduced odds of CRCI	~140 participants (1 study)	Low ⊕⊕OO (downgraded for imprecision)
SOD2	Variants associated with increased odds of CRCI	~140 participants (1 study)	Low ⊕⊕OO (downgraded for imprecision)
TNF	Evidence is mixed	~300 participants (2 studies)	Very low ⊕OOO (downgraded due to inconsistencies in the results)
Leukocyte DNA damage	Variants associated with increased odds of CRCI	~95 participants (1 study)	Low ⊕⊕OO (downgraded for imprecision)
PBMC Telomerase Activity	Variants associated with reduced odds of CRCI	~95 participants (1 study)	Low ⊕⊕OO (downgraded for imprecision)
PBMC Telomere Length	Null results	~95 participants (1 study)	Low ⊕⊕OO (downgraded for imprecision)
Mitochondrial DNA	Null results	~125 participants (1 study)	Low ⊕⊕OO (downgraded for imprecision)
Direct and oxidative DNA Damage—Comet Assay	Null results	~ 25 participants (1 study)	Very low ⊕OOO (downgraded for imprecision and some concerns about the risk of bias)
DNA methylation	Variants associated with increased odds of CRCI, except for DDHD1 and HSD17B3	~160 participants (2 studies)	Very low ⊕OOO (downgraded for imprecision and some concerns about the risk of bias)

* Certainty ratings use standard GRADE symbols: ⊕⊕⊕⊕ = high, ⊕⊕⊕O = moderate, ⊕⊕OO = low, ⊕OOO = very low.

## Data Availability

No new data were created for this review.

## Supplementary Materials

The following supporting information can be downloaded at: https://www.mdpi.com/article/10.3390/cancers17213522/s1, Table S1. PRISMA 2020 Checklist; Table S2. PRISMA 2020 for Abstracts Checklist. Table S3. Assessment timepoints. The table shows the different assessment timepoints for each study, as well as the number of assessments to collected outcome measures for both biomarkers and cognitive tests. Table S4. Cancer treatment. The table shows cancer treatment regimens of participants in each study, specifying whether they were exposed to radiotherapy (RT), endocrine therapy (ET), chemotherapy (ChT) or target therapy. Specific treatments are explicited. Of note there is no column for immunotherapy since no study reported information about it. Table S5. Outcome measures. The table displays the biomarker measures assessed in each study, specifying type, source of biomarker collection, quality of measurement and name of biomarker, as well as the cognitive measures administered in each study, distinguishing between objective cognitive assessment and self-reported measures. Cognitive domains assessed are also reported. Table S6. Results on biochemical biomarkers. The table shows main results of each study on the relationships between biochemical biomarkers and self-reported cognitive assessment and objective cognitive assessment, separately. Main negative results are also reported as well as the relevant statistic of interest. 95% confidence intervals (CIs) were reported by the original studies or calculated by the authors when possible; CIs are not applicable for nonparametric rank tests (Friedman/Wilcoxon), and not computed when models reported only χ^2^ and p without coefficients/SEs. Table S7. Results on biochemical biomarkers. The table shows main results of each study on the relationships between genetic biomarkers and self-reported cognitive assessment and objective cognitive assessment, separately. Main negative results are also reported as well as the relevant statistic of interest. 95% confidence intervals (CIs) were reported by the original studies or calculated by the authors when possible; CIs are not applicable for nonparametric rank tests (Friedman/Wilcoxon), and not computed when models reported only χ^2^ and p without coefficients/SEs.

## Abbreviations

The following abbreviations were used in this manuscript and the Supplementary Materials:

**Cognitive Tests/Scales/Questionnaires**	
ANAM	Automated Neuropsychological Assessment Metric
BCPT	Breast Cancer Prevention Trial Symptom Checklist
BCT	Brief Cognitive Test
BRIEF-A	Behavior Rating Inventory of Executive Function-Adult Version
BTACT	Telephone Brief Test of Adult Cognition
BVMT-DR	Brief Visuospatial Memory Test—Delayed Recall
BVMT-R	Brief Visuospatial Memory Test Revised
BVMT-TR	Brief Visuospatial Memory Test—Total Recall
CANTAB	Cambridge Neuropsychological Test Automated Battery
CNSVS	Central Nervous System Vital Signs™
COWA/COWAT	Controlled Oral Word Association
CPT-3	Continuous Performance Test—Third Edition
CTT	Color Trails Test
CVLT-2	California Verbal Learning Test 2nd Edition
D-KEFS	Delis-Kaplan Executive Function System
DLCT	Double Letter Cancelation Task
DMS	Delayed Matching-to-Sample
DST	Digit Span Test
EBPM	Event-Based Prospective Memory Task
FACT-Cog	Functional Assessment of Cancer Therapy-Cognitive Function
FACT-PCA	Functional Assessment of Cancer Therapy—Perceived Cognitive Abilities
FACT-PCI	Functional Assessment of Cancer Therapy—Perceived Cognitive Impairments
HVLT-D	Hopkins Verbal Learning Test—Delayed Recall
HVLT-I	Hopkins Verbal Learning Test—Immediate Recall
HVLT-R	Hopkins Verbal Learning Test-Revised
LNS	Letter–Number Sequencing
MASQ	Multiple Ability Self-Report Questionnaire
MMQ	Multifactorial Memory Questionnaire Ability Scale
MMSE	Mini Mental State Examination
MOCA	Montreal Cognitive Assessment
NAB	Neuropsychological Assessment Battery
NABC	Neuropsychological Assessment Battery—Cognition
NART	National Adult Reading Test
NART-R	National Adult Reading Test-Revised
NIHTB-CB	NIH Toolbox Cognition Battery
OFT	Orthographical Fluency
OTS	One-Touch Stockings of Cambridge
PAOFI	Patient Assessment of Own Functioning Inventory
PASAT	Rao Paced Auditory Serial Addition Test
PMRQ	Penn Memory-Related Questionnaire
PRMQ	Prospective and Retrospective Memory Questionnaire
PROMIS	Patient-Reported Outcomes Measurement Information System
PVT	Psychomotor Vigilance Test
RAVLT	Rey Auditory-Verbal Learning Test
ROCF	Rey Osterreith Complex Figure
RVP	Rapid Visual Information Processing
SDMT	Symbol Digit Modalities Test
SMQ	Squire Memory Questionnaire
STAI-S	State-Trait Anxiety Inventory-State Scale
TBPM	Time-Based Prospective Memory Task
TMT A & B	Trail Making Test A & B
VFT	Verbal Fluency Test
VPA	Verbal Paired Associates
VRM	Verbal Recognition Memory
WAIS	Wechsler Adult Intelligence Scale
WASI	Wechsler Abbreviated Scale of Intelligence
WRAT	Wide Range Achievement Test
WTAR	Wechsler Test of Adult Reading
**Cognitive domains**	
APE	Attention—Processing Speed—Executive function
GDS	Global Deficit Score
LM	Logical Memory
PM	Prospective Memory
RM	Recognition Memory
RM	Retrospective Memory
**Biomarkers**	
ACTH	Adrenocorticotropic Hormone
AKAP6	A-Kinase Anchoring Protein 6
ALDH2	Aldehyde Dehydrogenase 2
ANKK1	Ankyrin Repeat and Kinase Domain Containing 1
APBA1	Amyloid Beta Precursor Protein-Binding Family A Member 1
APOE	Apolipoproteina E
ARPP21	cAMP-Regulated Phosphoprotein 21
AURKA	Aurora Kinase A
Aβ	Amyloid-Beta
BAG1	BCL-2-Associated Thanogene 1
BCL	B-Cell Lymphoma
BDNF	Brain-Derived Neurotrophic Factor
BIRC5	Baculoviral IAP Repeat Containing 5
BRCA	Breast Cancer Susceptibility Gene
CA153	Carbohydrate Antigen 153
CAT	Catalase
CCDC170	Coiled-Coil Domain Containing 170
CCNB1	Cyclin B
CD	Cluster of Differentiation
CDKN2B	Cyclin-Dependent Kinase Inhibitor 2B
CEA	Carcinoembryonic Antigen
CENPA	Centromere Protein A
CHD13	Chromodomain Helicase DNA-Binding Protein 13
CMC2	C-X9-C Motif Containing 2
COMT	Catechol-O-Methyltransferase
CpGs	Cytosine-Phosphate-Guanine Sites
CRP	C Reactive Protein
CTSL2	Cathepsin L2
CXCL12	C-X-C Motif Chemokine Ligand 12
CYP2D6	Cytochrome P450 Family 2 Subfamily D Member 6
DDHD1	DDHD Domain Containing 1
DGKA	Diacylglycerol Kinase Alpha
DHEA	Dehydroepiandrosterone
DIAPH3	Diaphanous-Related Formin 3
DNMT1 gene	DNA Methyltransferase 1 Gene
DRD2	Dopamine Receptor D2
ECE2	Endothelin-Converting Enzyme 2
ERBB2	Human Epidermal Growth Factor Receptor 2
ERCC	Excision Repair Cross-Complementation
ESR1	Estrogen Receptor 1
FGF-2	Fibroblast Growth Factor 2
FSH	Follicle-Stimulating Hormone
G/A	Guanine/Adenine
G-CSF	Granulocyte-Colony Stimulating Factor
GLR	Granulocyte-to-Lymphocyte Ratio
GM-CSF	Granulocyte-Macrophage Colony-Stimulating Factor
GnRH	Gonadotropin-Releasing Hormone
GPX1	Glutathione Peroxidase 1
GRB7	Growth Factor Receptor-Bound Protein 7
GST	Glutathione S-Transferase Genes
GSTM1	Glutathione S-Transferase Mu 1
HDL-C	High-Density Lipoprotein Cholesterol
HSD17B3	Hydroxysteroid 17-Beta Dehydrogenase 3
HTR2A	5-Hydroxytryptamine (Serotonin) Receptor 2A
IFNGR1	Interferon Gamma Receptor 1
IFN-γ	Interferon-γ
IGF-1	Insulin Growth Factor-1
IL1R1	IL1 Receptor Type 1
IL-1ra	IL-1 Receptor Antagonist
ILs	Interleukins
KLF5	Krüppel-Like Factor 5
LBP	Lipopolysaccharide-Binding Protein
LDL-C	Low-Density Lipoprotein Cholesterol
LH	Luteinizing Hormone
MCP-1	Monocyte Chemoattractant Protein-1
MDR1	Multidrug Resistance 1
MELK	Maternal Embryonic Leucine Zipper Kinase
MIP-1β	Macrophage Inflammatory Protein-1β
MIR125A	MicroRNA-125a
MLR	Monocyte-to-Lymphocyte Ratio
MMP11	Matrix Metallopeptidase 11
mtDNA	Mitochondrial DNA
MTHFR	Methylenetetrahydrofolate Reductase
MYBL2	MYB Proto-Oncogene-Like 2
NDC80	Nuclear Division Cycle 80
NDE	Neuron-Derived Exosomes
NDST3	N-Deacetylase/N-Sulfotransferase 3
NFE2L2	Nuclear Factor Erythroid 2 Like 2
NF-KB	Nuclear Factor Kappa B
NGF	Nerve Growth Factor
NLR	Neutrophil-Lymphocyte Ratio
ORC6	Origin Recognition Complex Subunit 6
PARP1	Poly (ADP-ribose) Polymerase 1
PBMC	Peripheral Blood Mononuclear Cell
PGR	Progesterone Receptor
PIV	Pan-Immune-Inflammation Value
PLR	Platelet-to-Lymphocyte Ratio
POC5	Proteome Of Centriole 5
PPFIBP2	Interacting Protein Binding Protein 2
RACGAP1	Rac GTPase Activating Protein 1
RASA2 gene	RAS p21 Protein Activator 2 Gene
RFC4	Replication Factor C Subunit 4
RIPOR2	Rho Family-Interacting Cell Polarization Regulator 2
RPS6KA1	Ribosomal Protein S6 Kinase A1
RRM2	Ribonucleotide Reductase Regulatory Subunit M2
rScO or rcSO^2^	Regional Cerebral Oxygen Saturation
SCUBE2	Signal Peptide-CUB-EGF Domain-Containing Protein 2
SEPP1	Selenoprotein P Plasma 1
SII	Systemic Immune-Inflammation Index
SLC6A4	Solute Carrier Family 6 Member 4
SNPs	Single Nucleotide Polymorphisms
SOD	Superoxide Dismutase
sTNFRI	TNF Receptor Type I
sTNFRII	Soluble Form of the TNF Receptor Type II
Tau	Tubulin-Associated Unit
TMEM161B	Transmembrane Protein 161B
TNF	Tumor Necrosis Factor
TNF-α	Tumor Necrosis Factor-Alpha
TOMM40	Translocase of Outer Mitochondrial Membrane 40
UBE2V1	Ubiquitin-Conjugating Enzyme E2 Variant 1
USP6NL	Ubiquitin Specific Peptidase 6 N-Terminal Like
VEGF-A	Vascular Endothelial Growth Factor
VLDL-C	Very-Low-Density Lipoprotein Cholesterol
**Other**	
AI	Aromatase Inhibitor
AIC	Akaike Information Criterion
BC	Breast Cancer
BMI	Body Mass Index
CALM	Managing Cancer and Living Meaningfully
ChT	Chemotherapy
Chr	Chromosome
CI	Confidence Interval
CMDE	Cyclophosphamide—Methotrexate—Doxorubicin—Etoposide
CMF	Cyclophosphamide—Methotrexate—5-FU
CogPCA	Cognitive Principal Component Analysis
CRCI	Cancer-Related Cognitive Impairment
EDTA	Ethylenediaminetetraacetic Acid
ER	Estrogen Receptor
ET	Endocrine Therapy
FAC	Fluorouracil-Adriamycin-Cyclophosphamid
GED	General Educational Development
GEE	Generalized Estimating Equation
GMM	Growth Mixture Modeling
HC	Healthy Controls
HER-2	Human Epidermal Growth Factor Receptor 2
MCID	Minimal Clinically Important Difference
NR	Not Reported
OR	Odds Ratio
PR	Progesterone Receptor
RCTs	Randomized Controlled Trials
RT	Radiotherapy
SD	Standard Deviation
TT	Targeted Therapy

## Appendix A

**Table A1:** Summary of results on non-genetic studies. The table shows a summary of evidence on link between biochemical biomarkers and CRCI.

Non-Genetic Biomarkers and Associationa with CRCI			Positive	Negative	Mixed	Null	Overall
**Inflammation-related markers**							
	**Immune-related markers** (cytokines, including pro-inflammatory markers and anti-inflammatory markers)						
		IL-1β	[17,18,49,53,60,68,69,72]	[55,81]		[44,52,63,73,79]	Mixed
		IL-2	[17,52]			[44,48,60,63,72,73]	Mixed
		IL-4	[17,49,68,69]	[16,44,60,81]		[48,52,72,73,79]	Mixed
		IL-5	[44]		[60]	[17,52]	Mixed
		IL-6	[18,41,48,62,72]	[16,60]		[17,38,39,50,52,53,54,55,63,71,73,79,82]	Mixed
		IL-7		[17]		[52]	Mixed
		IL-8	[16,18,52,72,73]	[17,48,55,60]		[53,62,79]	Mixed
		IL-10	[18,44,52,62]	[16,17]		[48,54,60,72,73,79]	Mixed
		IL-12	[18]		[60]	[17,44,73]	Mixed
		IL-13	[44,52]			[17,60]	Mixed
		TNF-α	[17,18,42,49,60,62,68,69]	[48,55,81]	[72]	[16,39,44,52,54,61,63,73,79]	Mixed
		IFN-γ	[18]		[60]	[17,44,52,62,63,72,73,79]	Mixed
		GM-CSF	[17,52]	[60]		[72,73,79]	Mixed
		IL-1ra				[50,54,82]	Null
		CRP	[38,48]			[39,50,61,63,71,82]	Mixed
	Inflammatory indexes						
		NLR	[49,80]			[76]	Mixed
**Stress-axis markers**							
		Cortisol	[24]		[23]	[36]	Mixed
**Neurotrophin**							
		BDNF	[65]		[78]	[54,61,63,74,79]	Mixed

**Table A2:** Summary of results of genetic studies. The table shows a summary of evidence on links between genetic biomarkers and CRCI.

Genetic Biomarkers and Association with CRCI			Positive	Negative	Mixed	Null	Overall
**Genetic (polymorphism)**							
	ANKKI						
		rs1800497		[70]			Negative
	ALDH2						
		rs671	[49]				Positive
		rs886205	[77]				Positive
		rs4648328	[58]				Positive
		rs4767944	[58]				Positive
	APOE						
		rs429358	[56]			[45,59,70]	Mixed
		rs7412	[56]			[45,59]	Mixed
		SNP in ε4 allele (not specified)	[51]			[73]	Mixed
	BDNF						
		rs6265		[78]	[65]	[45,59,64,70]	Mixed
	CAT						
		rs511895		[58]			Negative
		rs769214		[58]			Negative
	CCNBI						
		rs164390 (SNP 102 GT in the promoter)	[57]				Positive
		rs350099 (SNP 957 CT in the promoter)	[58]				Positive
		rs350104 (SNP 457 CT in the promoter)	[58]				Positive
	COMT						
		rs165599		[45]		[59]	Mixed
		rs4680		[70]		[45,59,70]	Mixed
		rs737865	[59]			[45]	Mixed
	DIAPH3						
		rs1337652		[57]			Negative
		rs4547237		[57]			Negative
	DNMT1						
		rs2162560		[42]			Negative
	DRD2						
		rs6277		[70]			Negative
	ERCC2						
		rs13181	[58]				Positive
		rs3916874		[58]			Negative
		rs50872		[58]			Negative
	ERCC3						
		rs4150407		[35,58]			Negative
		rs4150477		[58]			Negative
		rs2134794	[58]				Positive
	ERCC5						
		rs751402	[58]	[35]			Mixed
		rs873601	[58]				Positive
		rs11069498	[58]				Positive
		rs2296147		[58]			Negative
	ESR1						
		rs488133			[57]		Mixed
		rs2234693				[58]	Null
		rs9340799				[58]	Null
	GPX1						
		rs1050450		[35]			Negative
	GSTM1						
		rs412543 (SNP 498 CG in the promoter)			[57]		Mixed
	HMCN1						
		rs76859653 (intronic region)	[66]				Positive
		rs78786199 (intergenic region)	[66]				Positive
	ILB						
		rs16944 (SNP 511 CT in the promoter)				[37]	Null
	IL6						
		rs1800795 (SNP 174 GC in the promoter)	[37]			[41]	Null
	MYBL2						
		rs11556379			[57]		Mixed
		rs2070235			[57]		Mixed
	PARP1						
		rs2271347		[38,58]			Negative
	PGR						
		rs1042838		[57]			Negative
		rs474320		[57]			Negative
		rs484389		[57]			Negative
		rs608995		[57]			Negative
	SEPP1						
		rs3877899	[58]				Positive
		rs230819		[58]			Negative
	SOD1						
		rs1041740		[58]			Negative
	SOD2						
		rs4880	[58]				Positive
		rs5746136	[58]				Positive
		rs8031	[58]				Positive
	TNF						
		rs1800629 (SNP 308 GA in the promoter)	[37]			[41]	Null
**Leukocyte DNA Damage**			[83]				Positive
**PBMC Telomerase Activity**				[58]			Negative
**PBMC Telomere Length**						[58]	Null
**Mitochondrial DNA**						[41]	Null
**Direct and Oxidative DNA Damage—Comet Assay**						[47]	Null
**DNA Methylation**							
		BDNF	[46]				Positive
		RASA2	[46]				Positive
		ECE2	[75]				Positive
		USP6NL	[75]				Positive
		PPFIBP2	[75]				Positive
		DDHD1				[75]	Null
		RIPOR2	[75]				Positive
		KLF5	[75]				Positive
		UBE2V1	[75]				Positive
		HSD17B3				[75]	Null
		DGKA	[75]				Positive
		RPS6KA1	[75]				Positive
