# Supplementary material for: Investigating the Trajectories of Association Between Biomarkers and Cancer-Related Cognitive Impairment in Patients with Breast Cancer: A Systematic Review
Source: Cancers (Basel). 2025 Oct 31;17(21):3522. doi: 10.3390/cancers17213522 (PMC12610328; doi:10.3390/cancers17213522)
Supplement: Supplementary file 1 [file cancers-17-03522-s001.zip › cancers-3893250-supplementary.pdf]

Supplementary Materials

Table S1. PRISMA 2020 Checklist.

Section and Topic	Item #	Checklist item	Location where item is reported
**TITLE**			
Title	1	Identify the report as a systematic review.	Page 1
**ABSTRACT**			
Abstract	2	See the PRISMA 2020 for Abstracts checklist.	Supplementary files Table S2
**INTRODUCTION**			
Rationale	3	Describe the rationale for the review in the context of existing knowledge.	Introduction (pages 2-4)
Objectives	4	Provide an explicit statement of the objective(s) or question(s) the review addresses.	Introduction (page 4)
**METHODS**			
Eligibility criteria	5	Specify the inclusion and exclusion criteria for the review and how studies were grouped for the syntheses.	Materials and Methods (pages 4-6); Table 1
Information sources	6	Specify all databases, registers, websites, organisations, reference lists and other sources searched or consulted to identify studies. Specify the date when each source was last searched or consulted.	Materials and Methods (pages 4-5)
Search strategy	7	Present the full search strategies for all databases, registers and websites, including any filters and limits used.	Table 1
Selection process	8	Specify the methods used to decide whether a study met the inclusion criteria of the review, including how many reviewers screened each record and each report retrieved, whether they worked independently, and if applicable, details of automation tools used in the process.	Materials and Methods (page 5)
Data collection process	9	Specify the methods used to collect data from reports, including how many reviewers collected data from each report, whether they worked independently, any processes for obtaining or confirming data from study investigators, and if applicable, details of automation tools used in the process.	Materials and Methods (page 5)
Data items	10a	List and define all outcomes for which data were sought. Specify whether all results that were compatible with each outcome domain in each study were sought (e.g. for all measures, time points, analyses), and if not, the methods used to decide which results to collect.	Materials and Methods (page 5)
	10b	List and define all other variables for which data were sought (e.g. participant and intervention characteristics, funding sources). Describe any assumptions made about any missing or unclear information.	Materials and Methods (page 5)
Study risk of bias assessment	11	Specify the methods used to assess risk of bias in the included studies, including details of the tool(s) used, how many reviewers assessed each study and whether they worked independently, and if applicable, details of automation tools used in the process.	Materials and Methods (page 6)
Effect measures	12	Specify for each outcome the effect measure(s) (e.g. risk ratio, mean difference) used in the synthesis or presentation of results.	Not applicable
Synthesis methods	13a	Describe the processes used to decide which studies were eligible for each synthesis (e.g. tabulating the study intervention characteristics and comparing against the planned groups for each synthesis (item #5)).	Materials and Methods (page 5)
	13b	Describe any methods required to prepare the data for presentation or synthesis, such as handling of missing summary statistics, or data conversions.	Materials and Methods (page 5)
	13c	Describe any methods used to tabulate or visually display results of individual studies and syntheses.	Materials and Methods (page 5)

Section and Topic	Item #	Checklist item	Location where item is reported
	13d	Describe any methods used to synthesize results and provide a rationale for the choice(s). If meta-analysis was performed, describe the model(s), method(s) to identify the presence and extent of statistical heterogeneity, and software package(s) used.	Materials and Methods (page 5)
	13e	Describe any methods used to explore possible causes of heterogeneity among study results (e.g. subgroup analysis, meta-regression).	Not applicable
	13f	Describe any sensitivity analyses conducted to assess robustness of the synthesized results.	Not applicable
Reporting bias assessment	14	Describe any methods used to assess risk of bias due to missing results in a synthesis (arising from reporting biases).	Not applicable
Certainty assessment	15	Describe any methods used to assess certainty (or confidence) in the body of evidence for an outcome.	Materials and Methods (page 5-6)
**RESULTS**			
Study selection	16a	Describe the results of the search and selection process, from the number of records identified in the search to the number of studies included in the review, ideally using a flow diagram.	Results (page 6); Figure 1
	16b	Cite studies that might appear to meet the inclusion criteria, but which were excluded, and explain why they were excluded.	Figure 1
Study characteristics	17	Cite each included study and present its characteristics.	Results (page 6-38); Tables 2-3
Risk of bias in studies	18	Present assessments of risk of bias for each included study.	Not applicable
Results of individual studies	19	For all outcomes, present, for each study: (a) summary statistics for each group (where appropriate) and (b) an effect estimate and its precision (e.g. confidence/credible interval), ideally using structured tables or plots.	Results (page 6-38); Figures (2-7); Supplementary materials (S3-S7)
Results of syntheses	20a	For each synthesis, briefly summarise the characteristics and risk of bias among contributing studies.	Results (page 6-38)
	20b	Present results of all statistical syntheses conducted. If meta-analysis was done, present for each the summary estimate and its precision (e.g. confidence/credible interval) and measures of statistical heterogeneity. If comparing groups, describe the direction of the effect.	Not applicable
	20c	Present results of all investigations of possible causes of heterogeneity among study results.	Not applicable
	20d	Present results of all sensitivity analyses conducted to assess the robustness of the synthesized results.	Not applicable
Reporting biases	21	Present assessments of risk of bias due to missing results (arising from reporting biases) for each synthesis assessed.	Not applicable
Certainty of evidence	22	Present assessments of certainty (or confidence) in the body of evidence for each outcome assessed.	Results (table 10-11)
**DISCUSSION**			
Discussion	23a	Provide a general interpretation of the results in the context of other evidence.	Discussion (pages 42-46)
	23b	Discuss any limitations of the evidence included in the review.	Discussion (pages 42-46)
	23c	Discuss any limitations of the review processes used.	Discussion (pages 42-46)
	23d	Discuss implications of the results for practice, policy, and future research.	Discussion (pages 42-46); Conclusion (page 46)

Section and Topic	Item #	Checklist item	Location where item is reported
**OTHER INFORMATION**			
Registration and protocol	24a	Provide registration information for the review, including register name and registration number, or state that the review was not registered.	Materials and Methods (page 4)
	24b	Indicate where the review protocol can be accessed, or state that a protocol was not prepared.	Protocol not prepared
	24c	Describe and explain any amendments to information provided at registration or in the protocol.	Not applicable
Support	25	Describe sources of financial or non-financial support for the review, and the role of the funders or sponsors in the review.	Funding (page 46)
Competing interests	26	Declare any competing interests of review authors.	Conflict of interest (page 46)
Availability of data, code and other materials	27	Report which of the following are publicly available and where they can be found: template data collection forms; data extracted from included studies; data used for all analyses; analytic code; any other materials used in the review.	Data Availability Statement (page 46)

Table S2. PRISMA 2020 for Abstracts Checklist.

Section and Topic	Item #	Checklist item	Reported (Yes/No)
**TITLE**			
Title	1	Identify the report as a systematic review.	Yes
**BACKGROUND**			
Objectives	2	Provide an explicit statement of the main objective(s) or question(s) the review addresses.	Yes
**METHODS**			
Eligibility criteria	3	Specify the inclusion and exclusion criteria for the review.	Yes
Information sources	4	Specify the information sources (e.g. databases, registers) used to identify studies and the date when each was last searched.	Yes
Risk of bias	5	Specify the methods used to assess risk of bias in the included studies.	Yes
Synthesis of results	6	Specify the methods used to present and synthesise results.	Yes
**RESULTS**			
Included studies	7	Give the total number of included studies and participants and summarise relevant characteristics of studies.	Yes
Synthesis of results	8	Present results for main outcomes, preferably indicating the number of included studies and participants for each. If meta-analysis was done, report the summary estimate and confidence/credible interval. If comparing groups, indicate the direction of the effect (i.e. which group is favoured).	Yes
**DISCUSSION**			
Limitations of evidence	9	Provide a brief summary of the limitations of the evidence included in the review (e.g. study risk of bias, inconsistency	Yes

Section and Topic	Item #	Checklist item	Reported (Yes/No)
		and imprecision).	
Interpretation	10	Provide a general interpretation of the results and important implications.	Yes
**OTHER**			
Funding	11	Specify the primary source of funding for the review.	Yes
Registration	12	Provide the register name and registration number.	Yes

Table S3. Assessment timepoints. The table shows the different assessment timepoints for each study, as well as the number of assessments to collected outcome measures for both biomarkers and cognitive tests.

Author(s), Year	Pre-surgery	Post-surgery, pre-pharmacotherapy	During pharmacotherapy	Post-pharmacotherapy	Follow-up	Biomarker timepoints	Cognitive timepoints
Andreano et al., 2012 [1]			Assessment 1 during ET			1	1
Aspelund et al., 2024 [2]	Assessment 1 Pre-surg					1	1
Belcher et al., 2022 [3]		Assessment 1 before ChT		Assessment 2 after ChT		2	2
Bender et al., 2018 [4]		Assessment 1 pre-ChT or AI			Assessment 2 Post-ChT	1	4
Boivin et al., 2020 [5]		Assessment 1 (group of interest)	Assessment 2 during RT and 3 during ChT (group of interest)	Assessment (control group) Assessment 4 post-ChT (group of interest)	Assessments 2, 3, 4 (remission group)	4	4
Bower et al., 2013 [6]				Assessment 1 pre-ET		1	1
Boyle et al., 2017 [7]					Assessment 1	1	1
Carlson et al., 2018 [8]				Assessment 1 post-ChT		1	1

Carrol et al., 2019* [9]					Assessment 1	1	1
Chae et al., 2016* [10]		Assessment 1 pre-ChT	Assessment 2 during ChT	Assessment 3 post-ChT		3	3
Chae et al., 2018 [11]		Assessment 1 pre-ChT	Assessment 2 during ChT			2	2
Chan et al., 2019 [12]		Assessment 1 Pre-ChT	Assessment 2 during ChT	Assessment 3 post-ChT		1	3
Chen et al., 2021 [13]	Assessment 1 for HC	Assessment 1 for pre-ChT BC group		Assessment 1 for post-ChT BC group		1	1
Cheng et al., 2016 [14]		Assessment 1 pre-ChT		Assessment 2 post-ChT		1	2
Cho et al., 2024 [15]		Assessment 1 pre-ET				1	1
Conroy et al., 2013 [16]					Assessment post-ChT	1	1
Duivon et al., 2024 [17]		Assessment 1 pre-ChT			Assessment 1 post-ChT	2	2
Gan et al., 2025 [18]				Assessment 1 post-ChT		1	1
Ganz et al., 2013 [19]		Assessment 1 pre-ET	Assessment 2 and 3 during ET			3	3
Harrison et al., 2021 [20]					Assessment 1	1	1
Henneghan et al., 2018 [21]				Assessment 1 post-ChT		1	1
Henneghan et al., 2021 [22]				Assessment 1 post-ChT		1	1
Janelsins et al., 2022 [23]		Assessment 1 pre-ChT		Assessment 2 post-ChT		2	2
Jenkins et al., 2016 [24]	Assessment 1 pre-surgery	Assessment 2 pre-ChT		Assessment 3 post-ChT		3	3

Keetile et al., 2023 [25]		Assessment 1 Pre-ChT	Assessment 2 during ChT	Assessment 3 post-ChT		3	3
Kesler et al., 2013 [26]				Assessment 1		1	1
Koleck et al., 2014 [27]		Assessment 1 Pre-ChT or ET	Assessment 2 and 3 for ET-group during ET	Assessment 2 for ChT+ET group pre-ET	Assessment 3 for ChT+ET group post-ET	1	3
Koleck et al., 2017 [28]		Assessment 1				1	1
Koleck, et al., 2016 [29]		Assessment 1				1	1
Li et al., 2020 [30]		Assessment 1 pre-ChT		Assessment 2 post-ChT		1	2
Lyon et al., 2016 [31]		Assessment 1 pre-ChT	Assessment 2 during ChT	Assessment 3 post-ChT	Assessment 4	5	5
Madison et al., 2023 [32]		Study 1: Assessment 1 Study 4: Assessment 1			Study 1: Assessments 2 and 3 post-treatment Study 2: assessments 1, 2 and 3 Study 3: Assessment 1 and 2 Study 4: assessment 2	study 1: 3 study 2: 3 study 3: 2 study 4: 2	Study 1: 3 Study 2: 3 Study 3: 2 Study 4: 2
Mandelblatt et al., 2023 [33]		Assessment 1			Assessment 2, 3, 4, 5, 6	6	6
Myers et al., 2022 [34]				Assessment 1	Assessment 2	3	3
Ng et al., 2016 [35]		Assessment 1 pre-ChT	Assessment 2 post-ChT	Assessment 3 post-ChT		1	3
Ng et al., 2017* [36]		Assessment 1 pre-ChT	Assessment 2 during ChT	Assessment 3 post ChT		3	3

Nudelman et al., 2023 [37]		Assessment 1			Assessment 2	1	2
Palesh et al., 2025 [38]			Assessment 1	Assessment 2	Assessment 3 and 4	4	4
Pang et al., 2021 [39]				Assessment 1 and 2 for CALM group post-ChT Assessment 1 for control group		2	2 (CALM group) 1 (control group)
Pang et al., 2023 [40]				Assessment 1		1	1
Park et al., 2025 [41]					Assessment 1 and 2	1	4
Patel et al., 2023 [42]			Assessment 1 before surgery or systemic therapy	Assessment 2	Assessment 3 and 4	4	4
Toh et al., 2020 [43]		Assessment 1 pre-ChT	Assessments 2 during ChT	Assessment 3 post-ChT	Assessment 4	3	4
Vardy et al., 2019* [44]				Assessment 1		1	1
Von Ah et al., 2022 [45]				Assessments 1 and 2		2	2
Yang et al., 2020 [46]		Assessment 1 pre-ChT		Assessment 2 post-ChT	Assessment 3	2	3
Yao et al., 2022 [47]				Assessment 1 and 2 post-ChT		2	2
Yao et al., 2023 [48]		Assessment 1 pre-ChT		Assessment 1 post-ChT		1	1
Yap et al., 2020* [49]		Assessment 1 pre-ChT	Assessment 2 during ChT	Assessment 3 post-ChT	Assessment 4	3	4
Yap et al., 2021 [50]		Assessment 1 pre-ChT	Assessment 2 during ChT	Assessment 3 post-ChT	Assessment 4	3	4
Yu et al., 2022 [51]		Assessment 1		Assessment 2		2	2

Zhao et al., 2020 [52]		Assessment 1 pre-ChT		Assessment 1 post-ChT		1	1
Zuniga et al., 2018				Assessment 1		1	1

*Note: studies with asterisk investigated both biochemical and genetic biomarkers.

Table S4. Cancer treatment. The table shows cancer treatment regimens of participants in each study, specifying whether they were exposed to radiotherapy (RT), endocrine therapy (ET), chemotherapy (ChT) or target therapy. Specific treatments are explicited. Of note there is no column for immunotherapy since no study reported information about it.

**Author(s), Year**	**RT**	**ET**	**ChT**	**Targeted therapy**	**Treatment regimens**
Andreano et al., 2012 [1]		X	X		ET: Luprolide (100.0%); aromatase inhibitors (30%); Tamoxifen (20%)<br>ChT: 5.0% in NR treatment; 70.0% history of NR ChT
Aspelund et al., 2024 [2]					No treatment
Belcher et al., 2022 [3]			X		ChT: 100.0% in NR treatment
Bender et al., 2018 [4]	X	X	X		RT: 71.0%<br>ET: 100.0% Aromatase inhibitor (anastrozole)<br>ChT prior to ET: 28.0% in NR treatment
Boivin et al., 2020 [5]	X	X	X		RT: [Remission group: 38.0%; Active group: 100.0%]<br>ET: Tamoxifen [Remission group: 69.0%; Active group: 40.0%]<br>ChT: Cyclophosphamide + Methotrexate + Fluorouracil [Remission group: 50.0%; Active group: 15.0%]; Taxotere (Docetaxel) [Remission group: 19.0%- Active group: 30.0%]
Bower et al., 2013 [6]	X	X	X		RT: 73.0%<br>ET: not yet started at enrollement (% NR)<br>ChT: 53.0% in NR treatment

Boyle et al., 2017 [7]	X		X	X	RT: 74.6%<br>ChT: 51.3% (Anthracycles 22.6% of ChT)<br>TT: Trastuzumab 14.8%
Carlson et al., 2018 [8]	X	X	X		RT: 80.0%<br>ET: 66.75 (Aromatase inhibitors 80% of ChT)<br>ChT: 100.0% in NR treatment
Carrol et al., 2019* [9]	X	X	X		RT only: 29.8%<br>ChT only: 11.7% in NR treatment<br>RT+ ChT: 42.6%<br>ET: 72.0%
Chae et al., 2016* [10]			X		ChT: Anthracycline (Doxorubicin 68.0%); Taxane (Docetaxel 32.0%)<br>No prior RT
Chae et al., 2018 [11]			X		ChT: Anthracyclines (Doxorubicin + cyclophosphamide 64.8%); Taxanes (Docetaxel + cyclophosphamide 35.2%)<br>ChT-naive and/or RT-naive
Chan et al., 2019 [12]			X		ChT-naive and/or RT-naive<br>ChT: Anthracyclines 64.7%; Taxane 35.3%
Chen et al., 2021 [13]	X	X	X		RT: Pre ChT 1.4%; Post ChT 83.3%<br>ET: Pre ChT 4.3%; Post ChT 77.8%<br>ChT:<br>Cyclophosphamide + methotrexate + 5-fluorouracil 8.3%;<br>Cyclophosphamide + methotrexate + epirubicin + doxorubicin 2.7%;<br>Cyclophosphamide + 5-fluorouracil + epirubicin + doxorubicin 11.1%;<br>Cyclophosphamide + 5-fluorouracil + epirubicin + doxorubicin + docetaxel 41.7%;<br>Cyclophosphamide + 5-fluorouracil + epirubicin + doxorubicin + docetaxel + cisplatin 11.1%
Cheng et al., 2016 [14]			X		No ET for inclusion criteria<br>ChT: 100.0% Doxorubicin + paclitaxel + cyclophosphamide + fluorouracil

Cho et al., 2024 [15]		X	X		ET: 96.9% in NR treatment<br>ChT: 17.6% in NR treatment
Conroy et al., 2013 [16]	X	X	X		RT: 79.2%<br>ET: Tamoxifen 54.2%; Aromatase inhibitor 70.8%<br>ChT:<br>Doxorubicin + cyclophosphamide 29.2%<br>Doxorubicin + cyclophosphamide + taxane 20.8%<br>Doxorubicin + cyclophosphamide + 5-fluorouracil 8.3%<br>Doxorubicin+ taxane 12.5%<br>Cyclophosphamide + methotrexate + 5-fluorouracil 8.3%<br>Cyclophosphamide + methotrexate + 5-fluorouracil + doxorubicin + cyclophosphamide + 5-fluorouracil 4.2%<br>Taxane only 4.2%<br>Doxorubicin + cyclophosphamide + taxane + capecitabine 8.3%<br>Taxane and capecitabine 4.2%
Duivon et al., 2024 [17]	X	X	X		RT: 95.0%<br>ET: 82.0%<br>ChT: 64.0% in NR treatment
Gan et al., 2025 [18]			X		ChT: 100.0% Anthracyclines + Taxanes (Paclitaxel)
Ganz et al., 2013 [19]	X	X	X		RT: 76.0%<br>ET: 72.0% (Tamoxifen 54.0%; Aromatase Inhibitor 46.0%)<br>ChT: Anthracyclines 15.1%
Harrison et al., 2021 [20]	X	X	X		RT: ChT group 73.0%; ChT naive 65.0%<br>ET: Tamoxifen ChT group 43.0%; ChT naive 58.0%<br>ChT: 50.6% of BC patients<br>Doxorubicin + cyclophosphamide + paclitaxel / docetaxel 38.1%<br>Cyclophosphamide + methotrexate +5-fluorouracil 9.5%<br>Doxorubicin + cyclophosphamide 26.2%<br>Cyclophosphamide + paclitaxel or docetaxel 21.4%<br>Doxorubicin + cyclophosphamide + 5-fluorouracil 2.4%<br>Epirubicin + cyclophosphamide + paclitaxel 2.4%

Henneghan et al., 2018 [21]	X	X	X		RT: 60.6%) %<br>ET: 84.6% (66.7% currently)<br>ChT: Anthracyclines 56.1%
Henneghan et al., 2021 [22]		X	X		ET: 66.7% currently<br>ChT: previously, NR treatment
Janelsins et al., 2022 [23]	X		X		RT: not concurrently while receiving ChT<br>ChT: Anthracyclines 53.8%; Other 46.2%
Jenkins et al., 2016 [24]	X	X	X	X	RT: ChT 87.5%; non ChT 83.3%<br>ET: ChT 37.5%; non ChT 100.0%<br>ChT: 57.1%<br>doxorubicin + cyclophosphamide 12.5%;<br>fluorouracil + epirubicin + cyclophosphamide 25.0%;<br>fluorouracil + epirubicin + cyclophosphamide + docetaxel 65.5%<br>TT: ChT 25.0% (Trastuzumab); non ChT 0.0%
Keetile et al., 2023 [25]			X		ChT:<br>cyclophosphamide + methotrexate + fluorouracil 46.9%;<br>fluorouracil + adriamycin + cyclophosphamide 53.1%
Kesler et al., 2013 [26]			X		ChT:<br>Doxorubicin + cyclophosphamide/paclitaxel 81.8%;<br>Cyclophosphamide + 5-fluorouracil + paclitaxel/ methotrexate =13.6%
Koleck et al., 2014 [27]		X	X		ET: 100.0% anti-estrogen therapy (anastrozole)<br>ChT: 47.4% in NR treatment
Koleck et al., 2017 [28]		X	X		ET: 100.0% anti-estrogen therapy (anastrozole)<br>ChT: 39.9% in NR treatment
Koleck, et al., 2016 [29]		X	X		ET: 100.0% anti-estrogen therapy (anastrozole)<br>ChT: 47.4% in NR treatment

Li et al., 2020 [30]		X	X		ChT: fluorouracil + cyclophosphamide + paclitaxel + doxorubicin (NR%)<br>ET: NR%
Lyon et al., 2016 [31]	X		X	X	RT: 79%<br>ChT:<br>docetaxel + doxorubicin 52.0%;<br>docetaxel + cyclophosphamide 28.0%;<br>docetaxel + carboplatin + trastuzumab 14.0%<br>cyclophosphamide + methotrexate + fluorouracil 3.0%<br>doxorubicin + cyclophosphamide 3.0%
Madison et al., 2023 [32]	X		X		Surgery only: 19.0%<br>Surgery + NR ChT 27.0%<br>Surgery + RT + NR ChT 32.0%
Mandelblatt et al., 2023 [33]	X	X	X		RT: 19.1%<br>ET: 74.2% (aromatase inhibitors 89.2%)<br>ChT ± ET: 25.8% in NR treatment
Myers et al., 2022 [34]	X	X	X	X	RT: 63.0%<br>ET: currently 67.0%<br>ChT: 100.0% in NR treatment<br>TT: currently Anti-HER2 therapy (%NR)
Ng et al., 2016 [35]			X		ChT-naive and RT-naive<br>ChT: Anthracyclines 64.8%; taxane 35.2%
Ng et al., 2017* [36]			X		ChT-naive and RT-naive<br>ChT: Anthracyclines 56.9%; Taxane 43.1%
Nudelman et al., 2023 [37]		X	X		ChT-naive and ET-naive<br>ChT: 25.8% in NR treatment<br>ET: 79.1%
Palesh et al., 2025 [38]		X	X		ET: 23.7%<br>ChT: 100.0% in NR treatment

Pang et al., 2021 [39]			X		ChT: 100.0% in NR treatment
Pang et al., 2023 [40]	X		X		RT+ ChT: 78.0%<br>ChT: 23% (Anthracyclines 43.8%; Taxane 97.9%; Platinum 25.8%; Cytoxan 65.6%)
Park et al., 2025 [41]	X		X		RT: 76.8%<br>ChT: 100% (Anthracycline 43.85; taxane 97.7%; platinum 25.8%; Cytoxan 65.6%)
Patel et al., 2023 [42]	X				Surgery Only: 20.8%<br>RT: 37.0%<br>ChT: 10.4%<br>(Only ChT or combined:<br>Adriamycin + Cytoxan 46.6%;<br>Carboplatin + Paclitaxel 4.1%;<br>Docetaxel 45.2%;<br>Cytoxan + Methotrexate + Fluorouracil 1.4%;<br>NR 2.7%)<br>ChT + RT: 31.8%<br>ET: 72.1%
Toh et al., 2020 [43]	X	X	X		ChT-naive and/or RT-naive<br>RT: 68.0%<br>ChT: 100.0% (Anthracyclines 68.0%; Taxane 32.0%)<br>ET: 82.8%
Vardy et al., 2019* [44]	X	X	X		RT: 76.2%<br>ChT: 76.2% in NR treatment<br>ET: 65.1% (at time of testing 57.1%)
Von Ah et al., 2022 [45]	X	X	X		RT: Intervention 89.5%; Control 76.5%<br>ET/ChT: Intervention (Tamoxifen 57.9%; Aromatase inhibitor 42.1%); Control (Tamoxifen 29.4%; Aromatase inhibitor 58.8%)
Yang et al., 2020 [46]	X		X		Neoadjuvant: 10.3%<br>RT: 75.9%<br>ChT:<br>Doxorubicin + cyclophosphamide 1.7%;

					Cyclophosphamide + methotrexate + fluorouracil 3.4%;<br>Docetaxel + doxorubicin + cyclophosphamide 43.1%;<br>Docetaxel + cyclophosphamide 32.8%<br>Docetaxel + carboplatin + trastuzumab 19.0%
Yao et al., 2022 [47]			X		ChT: 100.0% (Anthracyclines %NR; Taxanes %NR)
Yao et al., 2023 [48]			X		ChT-naive and RT-naive<br>ChT: 100.0% in NR treatment
Yap et al., 2020* [49]	X	X	X		RT post ChT: 66.1%<br>ChT: 100.0% (Anthracyclines 69,0%; Taxanes 31,0%)<br>ET post ChT: 80.5% (Aromatase inhibitor 35.1%; Tamoxifen 45.4%)
Yap et al., 2021 [50]	X	X	X		RT post ChT: 71.3%<br>ChT: 100.0% (Anthracyclines 70.6%; Taxanes 29.4%)<br>ET: 80.5% post ChT (Aromatase inhibitor 35.3%; Tamoxifen 47.8%)
Yu et al., 2022 [51]			X		ChT: 64.7% (Anthracyclines %NR; Taxanes %NR)
Zhao et al., 2020 [52]			X		ChT: 73.8% (Anthracyclines %NR; Taxanes %NR)
Zuniga et al., 2018 [53]	X	X	X		RT: BCS 27.6%; Control 0.0%<br>ChT: 34.5% (in NR treatment); Control 0.0%<br>ChT + RT: 37.9%; Control 0.0%<br>ET: 65.5%; Control 0.0%

*Note: studies with asterisk investigated both biochemical and genetic biomarkers.

Table S5. Outcome measures. The table displays the biomarker measures assessed in each study, specifying type, source of biomarker collection, quality of measurement and name of biomarker, as well as the cognitive measures administered in each study, distinguishing between objective cognitive assessment and self-reported measures. Cognitive domains assessed are also reported.

Author(s), year	Type	Source	Quality of measurement	Name/identifier of biomarker	Objective cognitive assessment	Subjective cognitive assessment	Cognitive domains assessed
Andreano et al., 2012 [1]	Neuroendocrine	Saliva	Good	Neuroendocrine: Cortisol; Estradiol; Progesterone	WMS-III verbal paired associates I test; logical memory I test (week 0 and 1);<br><br>Computerized n-bask test (only week 2)		Verbal memory; Working memory
Aspelund et al., 2024 [2]	Neuroendocrine; Common laboratory tests	Saliva	Good	Neuroendocrine: Cortisol<br><br>Common laboratory tests: α-amylase	PVT; TMT (A + B); WAIS-IV (Digit Span); RAVLT; COWAT	PROMIS (Cognitive Function)	Attention (Sustained); Reaction time; Processing speed; Working memory; Executive functions; Verbal memory and learning; Verbal fluency; Self-reported cognition
Belcher et al., 2022 [3]	Immune-related (cytokine)	Blood (Serum)	Good	Immune-related (cytokine): IL-4; IL-6; IL-8; IL-10;	CANTAB (Rapid Visual Processing Task); Backward		Attention (sustained and visual); Processin

				TNF-α; sTNFRI; sTNFRII	counting task; TMT (A)		g speed; Visual scanning
Bender et al., 2018 [4]	Genetic (polymorphism)	Saliva, blood (whole blood)	Not reported	Genetic (polymorphism): 39 functional and tagging SNPs for DNA repair genes (ERCC2, ERCC3, ERCC5, PARP1) and oxidative stress genes (CAT, GPX1, SEPP1, SOD1, SOD2)<br><br>Genotypes were also determined for the two functional SNPs (rs429358 and rs7412) that comprise the e2, e3, and e4 alleles of the APOE gene.	(see Bender et al., 2015): -Digit Vigilance; CANTAB; Rivermed Story; RAVLT ROCF; D-KEFS Verbal Fluency; TMT B; D-KEFS Stroop Test; Digit Symbol Substitution; NART-R		Concentration, working memory, executive function; Attention; Learning and memory; Executive function; Mental flexibility; Psychomotor efficiency; Visuospatial ability

Boivin et al., 2020 [5]	Immune-related (cytokine); common laboratory tests	Blood (Plasma, serum)	Very Good	Immune-related (immune cell markers): CD3; CD4; CD8; CD19; CD16/CD56<br><br>Common laboratory test: Natural-killer cell activity; Total white-blood-cell count; Micro-hematocrit; Total plasma protein; Toxicity-preventing activity; Plasma lipid-density profile<br><br>Neuroendocrine: Cortisol	ANAM clinical battery		Psychomotor speed; Attention (vigilance); Processing speed; Working memory; Short-term memory; Learning memory; Reasoning (spatial and analytic); Executive functions
Bower et al., 2013 [6]	Genetic (polymorphism)	Blood (whole blood)	Good	Genetic (polymorphism): SNPs in the promoter regions of three cytokine genes: ILB 511 CT (rs16944); IL6 174 GC (rs1800795); and TNF 308		SMQ	Self-reported memory

				GA (rs1800629)			
Boyle et al., 2017 [7]	Immune-related (cytokine); common laboratory tests	Blood (Plasma)	Poor	Immune-related (cytokine): IL-6<br><br>Common laboratory tests: CRP	Dot-prob attentional task; TMT (A)		Attention
Carlson et al., 2018 [8]	Immune-related; common laboratory test; neuroendocrine	Blood (Serum)	Good	Immune-related (cytokine): IL-6; TNF-α<br><br>Common laboratory test: CRP<br><br><br>Neuroendocrine: IGF-1	MoCA; FCSRT (computerized version)		Global cognition; Episodic memory
Carrol et al., 2019* [9]	Genetic	Blood (Plasma)	Medium	Genetic: Leukocyte DNA damage; PBMC Telomerase Activity; PBMC Telomere Length	CVLT-II; WMS-IV LM; BVMT-R; Rey-Osterrieth Complex Figure Test; TMT A & B; PASAT; WAIS-IV (Block Design); Verbal Fluency	FACT-Cog	Self-reported cognition; Learning & memory; Attention & processing speed; Visuospatial abilities; Executive functions

Chae et al., 2016* [10]	Immune-related (cytokine); genetic (polymorphism)	Blood (Plasma, buffy coat)	Good	Immune-related (cytokine):<br>IL-6; TNF-α<br><br>Genetic (polymorphism):<br>IL6-174 (rs1800795 G>C)<br>TNF-308 (rs1800629 G>A)	Neuropsychological assessment tools using HeadminderTM	FACT-Cog	Self-reported cognition; Objective cognitive function
Chae et al., 2018 [11]	Genetic (mitochondrial DNA)	Blood (Plasma, buffy coat)	Not reported	Genetic (mitochondrial DNA):<br><br>Mitochondrial DNA	CANTAB	FACT-Cog	Self-reported cognition; Memory; Attention; Executive functions
Chan et al., 2019 [12]	Genetic (Polymorphism)	Blood (Buffy coat)	Good	Genetic (polymorphism):<br>DNMT1 rs2162560	Headminder; CANTAB (Headminder used for the first half of the sample, then discontinued production and authors shifted to CANTAB)	FACT-Cog	Self-reported cognition; Processing speed; Response speed; Memory; Attention
Chen et al., 2021 [13]	Immune-related (cytokine)	Blood (Not reported)	Not reported	Immune-related (cytokine):<br>From helper T cell type 1 (IFN-γ; IL-12p70; IL-1β; IL-2; TNF-α);<br>Type 2 (IL-4;	Block Design; Digit Symbol Substitution; WAIS-III (Digit Span); Color Trails Test, Part 1; Color Trails Test, Part 2;	FACT-Cog	Visuospatial construction; Processing speed; Attention; Working memory;

				IL-5; IL-10; IL-13); Type 17 (IL-5; IL-17A)	Semantic Fluency; OFT; Word List Immediate Recall; Word List Long-Delay Recall ; Word List Recognition; WMS-III (Prospective Memory); Global Deficit Score		Executive function; Verbal fluency; Learning & memory; Prospective memory; Self-reported cognition
Cheng et al., 2016 [14]	Genetic (polymorphism)	Blood (whole blood)	Good	Genetic (polymorphism): SNPs of COMT (rs165599; rs4680; rs737865); APOE (rs429358; rs7412); BDNF (rs6265)	MMSE; VFT; Digit span	PMRQ	Global cognition; Verbal fluency; Short-term memory; Self-reported RM and PM
Cho et al., 2024 [15]	Genetic (Epigenetic)	Blood (whole blood)	Good	Genetic (epigenetic): DNA methylation of BDNF and RASA2	Digit Vigilance tests	PAOFI	Processing speed; Self-evaluation of intellectual functioning
Conroy et al., 2013 [16]	Genetic (DNA damage)	Blood (whole blood)	Good	Genetic (DNA damage): Direct and oxidative DNA	RAVLT; BLT; WAIS-III; PASAT; WRAT-4 (Word reading test); WASI	MASQ; FACT-cog	Learning; Memory; Attention; Language; Visuospatial abilities;

				Damage-Comet Assay	(Vocabulary test; Block design test); Digit span; COWA; D-KEFS color-word interference test; TMT; SDMT		Executive functions; Psychomotor; Self-reported cognition; Self-reported mood and anxiety
Duivon et al., 2024 [17]	Immune-related (cytokine); common laboratory tests	Blood (Not reported)	Not reported	Immune-related (cytokine): IL-2; IL-4; IL-6; IL-8; IL-10; TNFα<br><br>Common laboratory tests: CRP	HVLT; TMT (A + B); Stroop test; Spatial span; Digit span; Letter number sequencing; Symbol search		Episodic memory; Working memory; Processing speed; Attention; Executive function
Gan et al., 2025 [18]	Immune-related (cytokine); Common laboratory tests	Blood (Plasma)	Good	Immune-related (cytokine): IL-1β; TNF-α; IL-4<br><br>Common laboratory tests: NLR; PLR; MLR; GLR; SII	MMSE	PRMQ	Global Cognition; Self-reported RM and PM
Ganz et al., 2013 [19]	Immune-related (cytokine); common laboratory tests	Blood (Plasma)	Medium	Immune-related (cytokine): IL-1ra; sTNF-RII; IL-6<br><br>Common laboratory	WTAR; CVLT-2; WMS-III; BVMT-R; ROCF; WAIS-3; TMT (A + B); Stroop test	SMQ	Estimated IQ; Verbal Learning; Verbal Memory; Visual Learning; Visual Memory; Visuospat

				tests: CRP			ial Function; Psychomotor Speed; Executive Functions; Motor speed; Self-reported memory
Harrison et al., 2021 [20]	Genetic (Polymorphism)	Saliva	Good	Genetic (polymorphism): APOE; BDNF; COMPT; GST; MDR1	WAIS-IV (Symbol search); Digit span; NABC; DKEFS- letter fluency; HVLT-R	BRIEF	Processing speed; Working memory; Executive functions; Verbal memory
Henneghan et al., 2018 [21]	Immune-related (cytokine); common laboratory tests	Blood (Serum)	Very Good	Immune-related (cytokine): IL-1β; IL-2; IL-4; IL-5; IL-6; IL-7; IL-8; IL-10; IL-12p70; IL-13; TNF-α; IFN-γ; GM-CSF<br><br>Common laboratory tests: CRP	HVLT-R; TMT (A + B); COWAT	FACT-Cog	Self-reported cognition; Learning and memory (verbal); Executive functions; Language production
Henneghan et al., 2021 [22]	Immune-related (cytokine)	Blood (Serum)	Good	Immune-related (cytokine): TNF-α; GM-CSF; IFN-γ; IL-1β; IL-2; IL-2; IL-4; IL-5; IL-6; IL-7;		FACT-Cog (Perceived Cognitive Impairments subscale)	Self-reported cognition

				IL-8; IL-10; IL-13			
Janelsins et al., 2022 [23]	Immune-related (cytokine)	Blood (serum)	Good	Immune-related (cytokine): sTNFRI; sTNFRII; MCP-1; IL-6; IL-8	CANTAB battery (DMS, VRM, RVP, OTS); HVLT-R; TMT; COWA; Phone BTACT battery (RAVLT; Digits backward; Category fluency; Backward counting)		Verbal learning; Episodic memory; Working memory; Attention (sustained); Psychomotor speed; Executive functions; Verbal fluency
Jenkins et al., 2016 [24]	Immune-related (cytokine); neurotrophine	Blood (Serum)	Poor	Immune-related (cytokine): IL-6; IL-10; sTNF-RII; TNF-α<br><br>Neutrophine: BDNF	RAVLT; LNS; Stroop test; COWAT; DLCT	FACT-Cog	Memory; Processing speed; Executive function; Self-reported cognition
Keetile et al., 2023 [25]	Immune-related (cytokine)	Blood (Plasma)	Medium	Immune-related (cytokine): IL-1β; IL-6; IL-8; TNF-α		FACT-Cog	Self-reported cognition
Kesler et al., 2013 [26]	Immune-related (cytokine)	Blood (Serum)	Good	Immune-related (cytokine): IL-1β; IL-6; IL-8; IL-10;	HVLT	MMQ	Memory; Self-reported memory

				IL-12; IFN-γ; TNF-α			
Koleck et al., 2014 [27]	Genetic (Polymorphism)	Saliva, blood (whole blood)	Good	Genetic (polymorphism): APOE rs429358 and rs7412 in ε2; ε3; ε4 alleles	CANTAB (Spatial Working Memory Test; Stockings of Cambridge Test; Paired Associates Learning Test; Rapid Visual Information Processing Test); Digit Vigilance Test; Delis Kaplan Color Word Interference Test; Verbal Fluency Test; TMT-B; Digit Symbol Substitution Test; Rivermead Behavioral Memory Test; RAVLT; ROCF		Attention; Learning & Memory; Psychomotor speed; Mental flexibility; Executive function; Visuospatial ability
Koleck et al., 2017 [28]	Genetic (Polymorphism)	Saliva, blood (whole blood)	Good	Genetic (polymorphism): AURKA; BAG1; BCL2; BIRC5; CCNB1; CD68; CENPA; CMC2; CTSL2;	CANTAB (Rapid Visual Information Processing; Stockings of Cambridge; Spatial Working Memory; Paired Associates		Attention; Concentration; Executive function; Mental flexibility; Psychomotor speed; Verbal memory;

				DIAPH3; ERBB2; ESR1; GRB7; GSTM1; MELK MKI67; MMP11; MYBL2; NDC80; ORC6; PGR; RACGAP1; RFC4; RRM2; SCUBE2	Learning); Digit Vigilance Test; D-KEFS Color-Word Interference Test; Digit Symbol Substitution Test; Rey Complex Figure Test; RAVLT; ROCF; Verbal Fluency; Rivermed Story Test		Visual memory; Visual working memory
Koleck, et al., 2016 [29]	Genetic (Polymorphism)	Saliva, blood (whole blood)	Good	Genetic (polymorphism): CAT; GPX1; SEPP1; SOD1; SOD2; ERCC2; ERCC3; ERCC5; PARP1	CANTAB (Rapid Visual Information Processing; Stockings of Cambridge; Spatial Working Memory; Paired Associates Learning); Digit Vigilance; Delis Kaplan Color-Word Interference; Digit Symbol Substitution; RAVLT; Verbal Fluency; Rivermed Story; ROCF; NART		Attention; Concentration; Mental Flexibility; Executive Functions; Psychomotor Speed; Verbal Memory; Visual Memory; Visual Working Memory; Estimated verbal intelligence

Li et al., 2020 [30]	Genetic (polymorphisms)	Blood	Good	Genetic (polymorphisms): BDNF (rs6265); APOE (rs429358, rs7412); COMT (rs165599; rs4680; rs737865)	MMSE; VFT; DST; EBPM; TBPM		Global cognition; Verbal fluency; Short-term memory; Prospective memory
Lyon et al., 2016 [31]	Immune-related (cytokine)	Blood (Plasma)		Immune-related (cytokine): IL-1β; IL-2; IL-4; IL-5; IL-6; IL-7; IL-8; IL-10; IL-12(p70); IL-13; IL-17; G-CSF; GM-CSF; IFN-γ; MCP-1; MIP-1β; TNF-α	Performance-based computerized neurocognitive testing system; CNSVS		Memory; Psychomotor speed; Reaction time; Attention (complex); Cognitive flexibility
Madison et al., 2023 [32]	Immune-related (cytokine); common laboratory test	fasting blood	Not reported	Immune-related (cytokine): TNF-α; LBP<br>Common laboratory test: CRP	Only in study 4 TMT (A + B); Phonemic fluency; Semantic fluency; CPT-3; HVLT; n-back test	Kohli scale (Studies 1,2,3); BCPT cognitive subscale (studies 2,3,4)	Visual scanning; Set shifting; Verbal fluency; Attention (Sustained); Verbal learning; Memory; Working memory; Self-reported subjective cognitive

							functioning
Mandelblatt et al., 2023 [33]	Immune-related (cytokine)	Blood (platelet-poor EDTA plasma)	Very Good	Immune-related (cytokine): IL-6; IL-8; IL-10; TNF-α; IFN-γ	*APE domain* NAB (Digit forward; Digit Backward); TMT (A + B); COWAT; Digit symbol test<br><br>*LM domain* WAIS (Logical memory I and II); NAB (List A Immediate recall; List A Short delay recall; List long delay)	CES-D; STAI-S; FACT-Cog	Attention; Processing speed; Executive function; Learning; Memory; Self-reported cognition
Myers et al., 2022 [34]	Immune-related (cytokine); neurotrophine; neuroendocrine; common laboratory test	Blood (Fingerstick bloodspot sampling)	Good	Immune-related (cytokine): TNF-α; IL-1α; IL-1β; IL-2; IL-6; IFN-γ; FGF-2<br><br>Neurotrophine: BDNF; VEGF-A<br><br>Neuroendocrine: IGF-1	TMT (A + B)	FACT-Cog	Attention; Self-report cognition

				Common laboratory test: CRP			
Ng et al., 2016 [35]	Genetic (polymorphisms)	Blood	Good	Genetic (polymorphisms): BDNF Val66Met (rs6265)	Headminder ® web-based neuropsychological battery	FACT-Cog	Self-reported cognition; Processing speed; Response speed; Memory; Attention
Ng et al., 2017* [36]	Neurotrophine	Blood (Plasma, buffy coat)	Not reported	Neurotrophine: BDNF; Genetic		FACT-Cog	Self-report cognition
Nudelman et al., 2023 [37]	genetic	saliva or anticoagulated whole blood		SNPs; APOE	Digit span (forward and backward); TMT A; TMT B; COWAT; WAIS-III (Digit Symbol subtest); WSM-III (logical memory I and II); NAB (List Learning immediate - short delayed and long delayed recall)		Attention; Processing speed; Executive function; Learning and memory

Palesh et al., 2025 [38]	Other (Extracellular vesicles)	Blood (Whole blood)	Not reported	Other: NDE	HVLT; TMT (A + B); COWAT		Immediate and delayed recall; Visual scanning; Processing speed; Attention (Divided); Verbal fluency
Pang et al., 2021 [39]	Immune-related (cytokine)	Blood (plasma)	Poor	Immune-related (cytokine): IL-1β; TNF-α; IL-4	MMSE	PRMQ	General cognition; Self-reported PM and RM
Pang et al., 2023 [40]	Immune-related (cytokine)	Blood (plasma)	Poor	Immune-related (cytokine): IL-1β; TNF-α; IL-4	MMSE	PRMQ	Global cognition; Self-reported PM and RM

Park et al., 2025 [41]	Genetic (Polymorphism)	Saliva, blood (whole blood)	Good	Genetic (polymorphism): AK091365 rs1487441; AKAP6 rs17522122; ANKK1 rs1800497; APBA1 rs3897757; APOE rs429358; ARPP21 rs13098807; BCL11 A rs7581162; BDNF rs6265; CDKN2B rs1333049; CHD13 rs8055236; COMT rs4680 G/A Val158met; CXCL12 rs1746048; CYP2D6 rs5758605; DRD2 rs6277; HTR2A rs6313; HTR2A rs6314; Intergenic rs28714259; MTHFR rs1801133; NDST3 rs6838310; SLC6A4 rs16965628; TOMM40 rs10119;	WTAR; BVMT-R; HVLT-R; WMS-IV (logical memory I and II); WAIS-IV (digit span); WAIS-IV (CTT-1); WAIS-IV (CTT-2); Stroop test; COWAT	FACT-Cog	Visuospatial memory; Verbal memory; Logical memory; Attention; Executive functions; Verbal fluency; Self-reported cognition

				TMEM161 B rs6452790			
Patel et al., 2023 [42]	Immune-related (cytokine); common laboratory test	Blood	Not reported	Immune-related (cytokine): sTNF-RII; IL-6; IL-1RA<br><br>Common laboratory test: CRP	WAIS-IV (Digit Span)	BRIEF-A	Self-reported global cognition; Working memory
Toh et al., 2020 [43]	Immune-related (cytokine)	Blood (Plasma)	Good	Immune-related (cytokine): IFN-γ; TNF-α; IL-1β; IL-2; IL-4; IL-6; IL-		FACT-Cog	Self-reported cognition

				8; IL-10; GM-CSF			
Vardy et al., 2019* [44]	Immune-related (cytokine); common laboratory tests; genetic	Blood (not specified)	Not reported	Immune-related (cytokine): IL-1β; IL-2; IL-4; IL-6; IL-8; IL-10; IL-12; TNF-α; IFN-γ; GM-CSF<br><br>Common laboratory tests: Hemoglobin; Electrolytes; Creatinine; Liver function tests; Coagulation markers; Thrombin-antithrombin complexes; Prothrombin fragments 1 & 2; D-dimers; Homocysteine<br><br>Neuroendocrine: Estradiol; FSH; LH<br><br>Genetic: APOE genotype (ε4	CANTAB; Modified Six Elements Test; GDS	FACT-Cog	Self-reported cognition; Learning and memory (verbal); Attention (auditory); Working memory; Visual-motor coordination; Executive functions; Processing speed; Global cognitive status

				carrier status)			
Von Ah et al., 2022 [45]	Neurotrophine	Blood (Plasma)	Not reported	Neurotrophine: BDNF	RAVLT; Rivermead Behavioural Paragraph Recall; Digit span; SDMT; COWAT		Learning and episodic memory; Attention; Working memory; Processing speed; Verbal fluency; Executive functions
Yang et al., 2020 [46]	Genetic (Epigenetic)	Blood (whole blood)		Genetic (epigenetic): Methylation of ECE2; USP6NL; PPFIBP2; DDHD1; RIPOR2; KLF5; UBE2V1;	CNSVS computerized test battery		Memory; Psychomotor speed; Reaction time; Complex attention; Cognitive flexibility

				HSD17B3; DGKA; RPS6KA1			
Yao et al., 2022 [47]	Common laboratory tests	Blood (not specified)	Not reported	Common laboratory tests: PIV; NLR; PLR; MLR; GLR; SII	MMSE	FACT-Cog	Global cognition; Perceived cognitive impairment
Yao et al., 2023 [48]	Genetic (polymorphism)	Blood (whole blood)		Genetic (polymorphism): ALDH2 rs671_GG; rs886205_GG; rs4648328_CC; and rs4767944_TT	MMSE; Verbal fluency; Digit span		Global cognition; Executive functions; Working memory; Verbal fluency
Yap et al., 2020* [49]	Immune-related (cytokine); Genetic	Blood (Plasma; whole blood)	Good	Immune-related (cytokine): BDNF; Val66Met polymorphism (rs6265)	CANTAB	FACT-Cog	Executive function; Visuospatial working memory; Processing speed; Psychomotor response speed; Learning and episodic memory; Attention (sustained and vigilance); Self-

							reported cognition
Yap et al., 2021 [50]	Immune-related (cytokine); neurotrophine	Blood (Plasma, buffy coat)	Good	Immune-related (cytokine): IL-1β; IL-4; IL-6, IL-8, IL-10; TNF-α; IFN-γ; GM-CSF<br><br>Neurotrophine: BDNF		FACT-Cog	Self-perceived cognition
Yu et al., 2022 [51]	Common laboratory tests	Blood (Not reported)	Not reported	Common laboratory test: NLR; CEA; CA153	MMSE	PRMQ; FACT-Cog	Self-perceived PM and RM; Self-perceived cognition; Global cognition
Zhao et al., 2020 [52]	Immune-related (cytokine)	Blood (Plasma)	Good	Immune-related (cytokine)Immune-related (cytokine) IL-1β; IL-4; TNF-α	MMSE	PRMQ; FACT-Cog	Global cognition; Self-perceived PM and RM; Self-perceived cognition

Zuniga et al., 2018 [53]	Immune-related (cytokine); common laboratory tests	Blood (Serum, plasma)	Very Good	Immune-related (cytokine): CRP; IL-6; sTNF-RII; IL-1ra<br><br>Common laboratory tests: Carotenoids [(E/Z)-phytoene; Phytofluene; α-carotene, β-carotene; Lycopene; Lutein + zeaxanthin; β-cryptoxanthin]; Clinical Lipid Panel [Total cholesterol; HDL-C; LDL-C; VLDL-C; Triglycerides]	NIHTB-CB	FACT-Cog	Episodic memory; Working memory; Language; Processing speed; Self-perceived cognition

*Note: studies with asterisk investigated both biochemical and genetic biomarkers.

Table S6. Results on biochemical biomarkers. The table shows main results of each study on the relationships between biochemical biomarkers and self-reported cognitive assessment and objective cognitive assessment, separately. Main negative results are also reported as well as the relevant statistic of interest. 95% confidence intervals (CIs) were reported by the original studies or calculated by the authors when possible; CIs are not applicable for nonparametric rank tests (Friedman/Wilcoxon), and not computed when models reported only χ^2 and p without coefficients/SEs.

Author(s), Year	Relevant statistics	Main results on biochemical biomarkers and self-reported cognition	Main results on biochemical biomarkers and objective cognition	Null findings

Andreano et al., 2012 [1]	t-test; two-way ANOVA	/	BC group showed reduced retention on the logical recall test at week 2 (*F* [1,36] = 4.465, $p < 0.05$, $\eta 2$ = 0.019, 95% CI not available; insufficient data) independent of stress condition.<br><br>For emotionally arousing material, a drug x stress condition interaction was observed (*F* [3,36] = 2.792, $p < 0.05$, $\eta 2$ = 0.018, 95% CI not available; insufficient data); post-hoc tests showed stress enhanced recall in HC (*t* [17] = 2.649, $p < 0.05$, *d* = 1.27, 95% CI not available; insufficient data) but had no effect in BC (*t* [16] = -0.337, $p > 0.7$, 95% CI not available; insufficient data).<br>Cortisol-memory associations differed significantly between groups (*z* = 2.18, $p < 0.05$, 95% CI not available; insufficient data); only HC showed a positive correlation between week 2 recall and cortisol 20 min post-stressor (*r* = 0.544, $p < 0.05$, *df* = 9, 95% CI [-0.08, 0.86]).<br><br>A drug x stress interaction was also reported for percent cortisol change at 30 min (*F* [3,36] = 4.496, $p < 0.05$, $\eta 2$ = 0.078, 95%	No significant cortisol changes were detected in BC at any time point following cold pressor stress.<br><br>No group differences were found between BC and HC in VPA, n-back, or story recall across timepoints.<br><br>In BC, recall did not differ between neutral and emotionally arousing stories.

			CI not available; insufficient data).	
Aspelund et al., 2024 [2]	Welch two-sample t-tests; multiple linear regression analysis<br><br>Global composite score was calculated with the mean z-scores of all neuropsychological outcomes	/	Model for biological predictors of overall cognitive function in BC patients was significant (F [4,28] = 3.6, p = 0.02, 95% CI not available; insufficient data), explaining 25% of the variance. Age (β = -0.44, p = 0.02) and steeper diurnal α-amylase slope (β = -0.42, p = 0.02) were significant predictors.<br><br>Model for biological predictors of processing speed was significant (F [4,41] = 5.7, p < 0.001, 95% CI not available; insufficient data), explaining 30% of the variance. Age was a strong predictor (β = 0.64, p < 0.001), while diurnal cortisol slope was marginally significant (β = 0.26, p = 0.05).	For verbal memory, multilinear regression models testing biological predictors and psychological predictors were non-significant.
Belcher et al., 2022 [3]	Linear mixed models; linear regression models	/	At post-ChT, higher sTNFRII was associated with worse RVP score (β = -1.316, SE = 0.587, p = 0.03, 95% CI [−2.467, −0.165]).<br><br>Longitudinally, higher pre-ChT IL-4 predicted improvement in RVP from pre- to post-ChT (β = 0.820, SE = 0.336, p = 0.02, 95% CI [0.161, 1.479]).	Cytokines and receptor levels showed no association with the interval between pre-ChT blood draw and first ChT administration, nor with the interval between last ChT administration and post-ChT blood draw.

			At pre-ChT, higher IL-8 was associated with worse BCT score (*β* = 0.610, SE = 0.241, *p* = 0.01, 95% CI [0.138, 1.082]).<br><br>At post-ChT, higher IL-8 (*β* = 0.841, SE = 0.260, *p* = 0.001, 95% CI [0.331, 1.351]), sTNFRI (*β* = 6.638, SE = 2.208, *p* = 0.003, 95% CI [2.310, 10.966]), and sTNFRII (*β* = 0.913, SE = 0.455, *p* = 0.045, 95% CI [0.021, 1.805]) were associated with worse BCT scores.<br><br>Longitudinally, higher pre-ChT sTNFRI predicted worse BCT performance over time (*β* = 5.566, SE = 2.367, *p* = 0.02, 95% CI [0.927, 10.205]).<br><br>Greater increases in IL-4 from pre- to post-ChT were associated with improved BCT performance (*β* = 0.564, SE = 0.253, *p* = 0.03, 95% CI [0.068, 1.060]).<br><br>At pre-ChT, higher IL-4 was associated with better TMT-A score (*β* = 1.098, SE = 0.516, *p* = 0.03, 95% CI [0.087, 2.109]), and higher IL-10 was also associated with better TMT-A score (*β* = 0.835, SE = 0.414, *p* =	

			0.04, 95% CI [0.024, 1.646]).	
Boivin et al., 2020 [5]	Tukey t test; repeated-measures ANOVA	/	In the active treatment group at 2 months, higher CD4 and CD8 levels were associated with poorer ANAM performance:<br><br>CD4 was negatively correlated with Code Substitution (*r* = -0.61, *p* < 0.05, 95% CI [−0.829, −0.229]), Digit-Set Comparison (*r* = -0.60, *p* < 0.05, 95% CI [−0.824, −0.214]), Mathematical Processing (*r* = -0.62, *p* < 0.05, 95% CI [−0.834, −0.245]), Running-Memory Continuous Performance (*r* = -0.58, *p* < 0.05, 95% CI [−0.814, −0.185]), Logical Reasoning-Symbolic (*r* = -0.69, *p* < 0.05, 95% CI [−0.868, −0.356]), and Spatial Processing-Simultaneous (*r* = -0.64, *p* < 0.05, 95% CI [−0.844, −0.275]).<br><br>CD8 was negatively correlated with Code Substitution (*r* = -0.74, *p* < 0.01, 95% CI [−0.891, −0.442]), Digit-Set Comparison (*r* = -0.68, *p* < 0.05, 95% CI [−0.863, −0.340]), and Mathematical Processing (*r* = -0.61, *p* < 0.05, 95% CI [−0.829, −0.229]).	For the BC survivor group (not on active treatment), no significant partial correlations were observed at any assessment point.<br><br>CD4, CD8, and CD16 were not significantly correlated with ANAM neuropsychological performance in the survivor comparison group.<br><br>After treatment completion, CD4 and CD8 were not significantly correlated with ANAM performance on any subtest.
Boyle et al., 2017 [7]	Multiple regression	CRP was significantly associated with	/	No significant association was

		greater attention to sad faces after controlling for demographic and treatment covariates (*β* = 6.92, *t*(85) = 2.11, *p* = 0.04, 95% CI ≈ [0.40, 13.44]), for depressive symptoms and TMT-A (*β* = 7.10, *t*(83) = 2.08, *p* = 0.04, 95% CI ≈ [0.31, 13.89]), and for medications (*β* = 6.81, *t*(82) = 1.99, *p* = 0.05, 95% CI ≈ [0.00, 13.62]), but not after adjusting for BMI (*β* = 6.31, *t*(81) = 1.59, *p* = 0.12, 95% CI ≈ [−1.59, 14.21]).<br><br>CRP was also significantly related to attentional bias away from happy faces after controlling for demographic, treatment-related, and medication covariates (*β* = 5.69, *t*(82) = 2.02, *p* = 0.046, 95% CI ≈ [0.09, 11.29]). However, this association became non-significant when additionally adjusting for depressive symptoms and TMT-A (*β* = 5.56, *t*(80) = 1.89, *p* = 0.062, 95% CI ≈ [−0.29, 11.41]) or BMI (*β* = 5.35, *t*(81)		observed between CRP and angry bias.<br><br>IL-6 did not predict attentional bias in any model.

		= 1.79, p = 0.077, 95% CI ≈ [−0.59, 11.29]).		
Carlson et al., 2018 [8]	Correlation, one-way ANOVAs	/	Lower MoCA scores were correlated with lower IGF-1 (r = 0.66, p = 0.007, 95% CI [0.22, 0.88]) and greater rcSO2 laterality (r = 0.58, p < 0.02, 95% CI [0.12, 0.84]).<br><br>Participants with serum IGF-1 < 85 ng/mL showed significantly greater cognitive impairment compared to those with higher IGF-1 (F [1,14] = 4.7, p = 0.05, 95% CI [.12, .84]*).<br><br>CRP levels were nearly twofold higher in participants with cognitive impairment (3.2 ± 0.7 vs. 1.7 ± 1.7 mg/L).<br><br>*Note: 95% CIs were derived via Fisher's r-to-z for the reported correlations (using their n's) and, for the IGF-1 group comparison, by converting F [1,14] to a point-biserial r and applying the same Fisher method	CRP, IL-6, and TNFα showed no significant associations with MoCA scores.
Carrol et al., 2019* [9]	Linear regression models	/	/	sTNF-RII did not significantly predict self-reported cognitive complaints after covariate adjustment and was not associated with any objectively measured cognitive domain.

Chae et al., 2016* [10]	Logistic regression, generalized estimating equation (GEE) model	Plasma IL-6 and TNF-α concentrations changed significantly across the three timepoints ($p < 0.001$), with peak levels observed at the end of ChT (Full test statistics were not reported).<br><br>Higher plasma IL-6 was strongly associated with worse self-perceived cognition (FACT-Cog total; $\beta = -0.036$, SE = 0.011, $p = .001$, 95% CI [−0.058, −0.014]).	/	Plasma IL-6 was not associated with cognitive domains assessed by HeadminderTM.<br><br>Plasma TNF-α showed no associations with either self-perceived or objective cognitive impairment.
Chen et al., 2021 [13]	Stepwise regression analyses; secondary multivariate regression analyses	In controls and pre-ChT patients:<br><br>Higher IL-5 was associated with greater subjective impairment (FACT-PCI; $\beta = -7.98$, $p = 0.025$, 95% CI [-14.87, -1.08]).<br><br>Higher IL-10 was associated with better self-rated cognitive abilities (FACT-PCA; $\beta = 3.88$, $p = 0.011$, 95% CI [0.97, 6.79].<br><br>Higher IL-13 was associated with worse self-rated cognitive abilities (FACT-PCA; $\beta = -2.28$, $p = 0.008$, 95% CI [-3.95, -0.62]).	/	Aside from IL-4, IL-5, IL-10, and IL-13, no other cytokines tested (IL-1β, IL-2, IL-12p70, TNF-α, IL-17A) were significantly associated with either objective or subjective (FACT-Cog) cognitive outcomes in multivariate models.<br><br>No significant differences in log-transformed cytokine values were observed between the three subgroups.

Duivon et al., 2024 [17]	Logistic regression models<br><br>(Composite z-score for 5 cognitive domains was created)	/	At year 2, overall cognitive impairment was associated with lower baseline IL-8 (OR = 0.85, p = 0.02, 95% CI [0.72, 0.98]) and higher baseline CRP (OR = 2.89, p = 0.03, 95% CI [1.10, 7.66]). In combined models, only CRP remained significant (OR = 2.84, p = 0.04, 95% CI [1.06, 7.64]).<br><br>High baseline IL-6 was associated with episodic memory impairment (OR = 5.50, p = 0.03, 95% CI [1.43, 36.6]).<br><br>High baseline CRP was associated with impaired processing speed (OR = 2.47, p = 0.04, 95% [CI 1.05, 5.87]).<br><br>Lower baseline TNFα was associated with working memory impairment (OR = 0.64, p = 0.01, 95% CI [0.44, 0.89]).	/
Gan et al., 2025 [18]	Multiple regressions, mediation analysis with process plug-in with the bootstrap method	SII as mediator: SII substantially mediated the relationship between distress and prospective memory. Direct effect: 0.316 (62.6%), 95% CI [-0.182, 0.814]; Indirect effect: 0.189 (37.4%), 95% CI [0.024, 0.406].<br><br>IL-1β as mediator: IL-1β partially	/	No significant group differences were found for TNFα or IL-4.<br><br>Immunoinflammatory markers PLR and MLR also showed no significant differences.

		mediated the relationship between distress and retrospective memory. Direct effect: 0.535 (80.7%), 95% CI [0.181, 0.889]; Indirect effect: 0.128 (19.3%), 95% CI [0.021, 0.256].		
Ganz et al., 2013 [19]	Repeated measures ANOVA; partial correlations	At baseline, mean plasma sTNFRII was higher in the ChT group than in the no-ChT group (2629 vs. 2149 pg/ml, p = 0.0015- full test statistic not reported, 95% CI not available; insufficient data), after controlling for age, BMI, and radiation.<br><br>sTNFRII levels declined significantly in the ChT group from Assessment 1 to 3 (p = 0.003 - full test statistic not reported, 95% CI not available; insufficient data), but remained unchanged in the no-ChT group.<br><br>In the ChT group, higher baseline sTNFRII was correlated with greater memory complaints (r = -0.21, p = 0.05, 95% CI not available; insufficient data) after adjusting for	/	/

		age, BMI, radiation, depression, and time since last ChT; the association was no longer significant when fatigue severity was included.<br><br>Decline in sTNFRII from Assessment 1 to 3 correlated with improvements in memory complaints in the ChT group (r = -0.34, p = 0.04, 95% CI [−0.58, −0.04], 95% CI not available; insufficient data).		
Henneghan et al., 2018 [21]	Machine learning: multivariate, non-parametric random-forest regressions	/	Immediate recall (HVLT-I; F = 10.02, adjusted R^2 = 0.71, p < .001): Predicted by IL-1β and IL-2 (plus education). Better scores were observed at IL-1β ~0.1-0.25 pg/mL and IL-2 < 0.2 pg/mL; higher values were associated with poorer recall. GM-CSF, IL-12p70, and IL-6 were not influential.<br><br>Delayed recall (HVLT-D; F = 11.80, adjusted R^2 = 0.75, p < .001): Predicted by IL-4, IL-1β, and TNF-α. Better performance occurred at IL-4 < 0.6 pg/mL, IL-1β < 0.1 pg/mL, and TNF-α ~0.2-0.7 pg/mL; higher values, particularly, were associated with worse outcomes.	/

			Executive function: models predicting Trails A and Trails B performance were significant ($F \approx 9–10$, adjusted $R^2 = 0.72–0.77$, $p < .001$). GM-CSF, IL-8, and IL-2 predicted faster Trails A; IL-1β and IL-2 predicted Trails B (IL-2 > 0.1 favorable).<br><br>Verbal fluency: Related to IL-10, IL-7, and IL-4 ($F = 10.07$, adjusted $R^2 = 0.72$, $p < .001$).<br><br>Perceived cognition: Driven primarily by psychosocial rather than cytokine factors ($F = 23.68$, adjusted $R^2 = 0.86$, $p < .001$).<br><br>Because random-forest regression was used, individual parameter estimates and confidence intervals were not available; therefore, no CIs are provided	
Henneghan et al., 2021 [22]	Correlation matrix; network analysis	Subjectively perceived cognitive impairment (FACT-Cog PCI, z-scores; higher = more symptoms) was negatively correlated with GM-CSF ($r = -0.34$, $p < 0.01$, 95% CI [−0.55, −0.10]).<br><br>IL-2 was also negatively correlated with	/	/

		subjective impairment after covariate adjustment (*r* = -0.27, $p < 0.05$, 95% CI [−0.49, −0.02]).		
Janelsins et al., 2022 [23]	Multivariable linear regression	/	Inflammatory changes: From pre- to post-ChT, IL-6, MCP-1, sTNFRI, and sTNFRII increased (all $p < 0.05$), while IL-1β decreased ($p = 0.003$). In controls, MCP-1 also increased ($p < 0.001$) but to a lesser extent.<br><br>IL-6 (change from baseline to post-ChT): Each 1-log-unit increase predicted poorer executive/fluency performance: more choices to reach correct plan in OTS ($\beta = +0.033 \pm 0.011$, $p = 0.004$, 95% CI [0.011, 0.055]), fewer problems solved on first try ($\beta = -0.298 \pm 0.127$, $p = 0.022$, 95% CI [−0.547, −0.049]), and fewer words on category fluency ($\beta = -0.548 \pm 0.255$, $p = 0.036$, 95% CI [−1.048, −0.048]).<br><br>MCP-1 (change): Each one-unit increase predicted slower processing speed on TMT-B ($\beta = +0.017 \pm 0.006$, $p = 0.006$, 95% CI [0.005, 0.029]).<br><br>IL-6 (baseline): Paradoxically, higher	/

			baseline IL-6 predicted better performance post-ChT: fewer OTS choices (*β* = -0.026 ± 0.009, *p* = 0.004, 95% CI [−0.044, −0.008]), more problems solved on first try (*β* = +0.328 ± 0.111, *p* = 0.004, 95% CI [0.110, 0.546]), and more words on category fluency (*β* = +0.515 ± 0.201, *p* = 0.014, 95% CI [0.121, 0.909]).	
Jenkins et al., 2016 [24]	Correlations	/	/	No significant correlations were found between cytokine levels and perceived cognitive function.
Keetile et al., 2023 [25]	Pearson's correlation	Mid-treatment with ChT: Higher IL-1β was associated with better self-perceived cognitive function (*r* = 0.205, *p* = .030, 95% CI [0.02, 0.38]), whereas higher TNF-*α* was associated with more self-perceived complaints (*r* = −0.191, *p* = .042, 95% CI [−0.36, −0.01]).<br><br>Post-treatment: Higher IL-8 was significantly associated with more self-perceived cognitive complaints (*r*= -0.185, *p* = 0.050, 95% CI [−0.36, −0.00]).	/	Baseline (pre-ChT): No associations were found between cytokine levels and FACT-Cog scores.

Kesler et al., 2013 [26]	Multiple linear regression analyses (forced entry)	/	BC group: Lower HVLT total performance was significantly associated with cytokine levels and left hippocampal volume (F = 3.48, adjusted R^2 = 0.482, p = 0.04, 95% CI not available; insufficient data). Significant contributions came from IL-6 × TNF-α interaction (β = 2.46, p = 0.006, 95% CI not available; insufficient data) and TNF-α × left hippocampus interaction (β = 3.28, p = 0.05, 95% CI not available; insufficient data).<br><br>Controls: Better HVLT delayed performance was associated with lower TNF-α (β = 0.642, p = 0.009, 95% CI not available; insufficient data) and larger left hippocampal volume (β = 0.455, p = 0.05; F = 4.66, adjusted R^2 = 0.268, p = 0.02, 95% CI not available; insufficient data). HVLT total and MMQ performance showed no associations in controls.	No other cytokine levels showed significant differences between groups.
Lyon et al., 2016 [31]	Multiple linear regression models: backwards model selection procedure with the Akaike information criterion	/	Baseline (pre-ChT): Better psychomotor speed with higher G-CSF (β = 4.60, t = 2.20, p < 0.04, 95% CI ≈ [0.50, 8.70]), worse with higher IL-17 (β = 2.80, t = -2.69, p < 0.01,	/

			95% CI ≈ [0.76, 4.84]). Faster reaction time with higher GM-CSF ($\beta = 1.00$, $t = 2.52$, $p < 0.02$, 95% CI ≈ [0.22, 1.78]). Better executive function with higher G-CSF ($\beta = 2.30$, $t = 2.12$, $p < 0.04$, 95% CI ≈ [0.17, 4.43]). Better cognitive flexibility with higher G-CSF ($\beta = 2.60$, $t = 2.33$, $p < 0.03$, 95% CI ≈ [0.41, 4.79]).<br><br>Mid-ChT: Better composite memory with higher IL-7 ($\beta = 1.60$, $t = 2.41$, $p < 0.02$, 95% CI ≈ [0.30, 2.90]). Better visual memory with higher IL-7 ($\beta = 1.80$, $t = 2.56$, $p < 0.02$, 95% CI ≈ [0.42, 3.18]). Better psychomotor speed with higher IL-5 ($\beta = 2.00$, $t = 2.18$, $p = 0.04$, 95% CI ≈ [0.20, 3.80]) and IL-17 ($\beta = 4.50$, $t = 4.94$, $p < 0.01$, 95% CI ≈ [2.72, 6.29]), worse with higher IL-1β ($\beta = 3.60$, $t = 3.19$, $p < 0.002$, 95% CI ≈ [1.39, 5.81]) and IL-12 ($\beta = 2.80$, $t = 3.16$, $p < 0.002$, 95% CI ≈ [1.06, 4.54]).<br><br>6 months post-ChT start: Better psychomotor speed with higher IL-17 ($\beta = 2.30$, $t = 2.08$, $p = 0.04$, 95% CI ≈ [0.13, 4.47]), worse with higher IL-1β ($\beta = 2.90$, $t = -2.03$, $p = 0.05$, 95% CI ≈ [0.10, 5.70]). Composite memory worse with higher GM-CSF (β =	

			1.30, $t = 2.38$, $p = .020$, 95% CI ≈ [0.23, 2.37]) and IL-7 ($\beta = 2.50$, $t = 2.40$, $p = .020$, 95% CI ≈ [0.46, 4.54]); better with higher IL-5 ($\beta = 1.80$, $t = 2.11$, $p = .039$, 95% CI ≈ [0.13, 3.47]) and IL-12 ($\beta = 1.20$, $t = 2.14$, $p = .036$, 95% CI ≈ [0.04, 2.36]). Faster reaction time with higher IL-7 ($\beta = 2.50$, $t = 2.50$, $p = .015$, 95% CI ≈ [0.54, 4.46]) and IL-17 ($\beta = 1.50$, $t = 2.67$, $p = .010$, 95% CI ≈ [0.40, 2.60]), slower with higher G-CSF ($\beta = 3.60$, $t = 3.45$, $p = .001$, 95% CI ≈ [1.56, 5.65]) and IFN-γ ($\beta = 2.40$, $t = 3.48$, $p = .001$, 95% CI ≈ [1.05, 3.75]). Better cognitive flexibility with higher IL-7 ($\beta = 3.30$, $t = 2.99$, $p = .004$, 95% CI ≈ [1.14, 5.46]). Better executive function with higher IL-7 ($\beta = 3.40$, $t = 3.11$, $p = .003$, 95% CI ≈ [1.26, 5.54]), worse with higher IL-10 ($\beta = 2.30$, $t = 3.68$, $p = .001$, 95% CI ≈ [1.08, 3.53]).<br><br>1 year post-ChT: Worse psychomotor speed with higher MCP-1 ($\beta = 2.62$, $t = 2.35$, $p = .022$, 95% CI ≈ [0.44, 4.81]) and MIP-1β ($\beta = 7.67$, $t = 2.45$, $p = .017$, 95% CI ≈ [1.53, 13.81]). Better complex attention with higher IL-4 ($\beta = 2.57$, $t = 3.32$, $p = .002$, 95% CI ≈ [1.05, 4.09]) and IL-6 ($\beta = 1.53$, $t =$	

			2.02, *p* = .048, 95% CI ≈ [0.05, 3.02]). Better executive function with higher IFN-γ (*β* = 2.33, *t* = 2.17, *p* = .034, 95% CI ≈ [0.23, 4.44]), IL-4 (*β* = 3.62, *t* = 3.26, *p* = .002, 95% CI ≈ [1.44, 5.80]), and IL-8 (*β* = 4.61, *t* = 2.94, *p* = .005, 95% CI ≈ [1.54, 7.68]).<br><br>2 years post-ChT: Worse composite memory with higher IL-7 (*β* = −2.72, *t* = −2.75, *p* = .008, 95% CI ≈ [−4.72, −0.72]). Worse visual memory with higher IL-7 (*β* = −2.85, *t* = −2.99, *p* = .004, 95% CI ≈ [−4.77, −0.93]). Better psychomotor speed with higher GM-CSF (*β* = +2.21, *t* = 2.37, *p* = .023, 95% CI ≈ [0.31, 4.11]) and IL-17 (*β* = +2.98, *t* = 2.99, *p* = .004, 95% CI ≈ [0.99, 4.97]), worse with higher MCP-1 (*β* = −2.91, *t* = −2.98, *p* = .004, 95% CI ≈ [−4.87, −0.95]) and TNF-*α* (*β* = −2.85, *t* = −2.92, *p* = .005, 95% CI ≈ [−4.82, −0.88]). Better complex attention with higher MIP-1β (*β* = +2.72, *t* = 2.81, *p* = .006, 95% CI ≈ [0.78, 4.66]), worse with higher MCP-1 (*β* = −2.62, *t* = −2.63, *p* = .012, 95% CI ≈ [−4.67, −0.57]). Worse verbal memory with higher IL-5 (*β* = −1.95, *t* =	

			−2.11, *p* = .040, 95% CI ≈ [−3.81, −0.09]).	
Madison et al., 2023 [32]	Generalized estimating equations (GEE)	Depression's association with Kohli focus scores was moderated by inflammation (X^2 = 6.65, *p* = 0.010, 95% CI not available; insufficient data). Depression predicted memory and focus problems at all inflammation levels (*p* < 0.021), with strongest effects at high inflammation.<br><br>In the same models, depression was associated with poorer cognitive function on BCPT (X^2 = 17.54, *p* < 0.001, 95% CI not available; insufficient data), Kohli focus (X^2 = 36.14, *p* < 0.001, 95% CI not available; insufficient data), and Kohli memory scales (X^2 = 19.08, *p* < 0.001, 95% CI not available; insufficient data).<br><br>A significant depression × intestinal permeability interaction predicted focus problems (X^2 = 4.32, *p* = 0.038, 95% CI not available; insufficient data) and marginally predicted memory	Inflammation × depression: The combination of high inflammation and depression predicted HVLT total recall (X^2 = 5.15, *p* = 0.023, 95% CI not available; insufficient data). Depressed patients recalled fewer words at average (X^2 = 8.90, *p* = 0.003, , 95% CI not available; insufficient data) and low (X^2 = 11.91, *p* = 0.0006, 95% CI not available; insufficient data) inflammation, but not at high inflammation (*p* = 0.58, full statistic not reported).<br><br>LBP × depression: Depression predicted slower Trail A completion time only at higher LBP (X^2 = 36.64, *p* = 0.010, 95% CI not available; insufficient data), not at average or low levels. Depression also predicted poorer HVLT recall at average (X^2 = 9.92, *p* = 0.002, 95% CI not available; insufficient data) and low (X^2 = 11.18, *p* = 0.0008, 95% CI not available; insufficient data), but not at high LBP.<br><br>Intestinal permeability: Greater permeability was associated with	Memory complaints were reported on the Kohli memory scale, but the trend was non-significant.<br><br>Depression, intestinal permeability, and inflammation did not significantly predict BCPT scores.<br><br>Inflammation alone was not associated with subjective cognitive function.<br><br>Sensitivity analyses: Substituting CRP for the inflammatory index showed only a marginal CRP × depression effect for Kohli focus, and no effect for Kohli memory.<br><br>LBP was not directly related to subjective cognitive function.<br><br>The combination of heightened inflammation and depression did not predict objective cognitive performance.<br><br>Depression and inflammation did not independently predict performance on any other neuropsychological test.

		problems ($X^2 = 2.95$, $p = 0.086$, 95% CI not available; insufficient data), with stronger associations for women with higher intestinal permeability.	slower response time on 2-back ($X^2 = 5.85$, $p = 0.016$, 95% CI not available; insufficient data) and slower Trail A completion time ($X^2 = 5.07$, $p = 0.024$, 95% CI not available; insufficient data).	
Mandelblatt et al., 2023 [33]	Multilevel mediation analyses	/	Survivors had significantly lower adjusted APE scores than controls across all models:<br>IL-6 model: $\beta = -0.097$, SE = 0.038, $p < .05$, 95% CI [−0.172, −0.023].<br>IL-10 model: $\beta = -0.095$, SE = 0.038, $p < .05$, 95% CI [−0.170, −0.020].<br>TNF-α model: $\beta = -0.095$, SE = 0.038, $p < .05$, 95% CI [−0.170, −0.020].<br><br>Higher IL-6, IL-10, and TNF-α were associated with lower APE scores across six neurocognitive tests:<br>IL-6-APE: $\beta = -0.106$, SE = 0.036, $p < .01$, 95% CI [−0.177, −0.035].<br>IL-10-APE: $\beta = -0.100$, SE = 0.034, $p < .01$, 95% CI [−0.167, −0.033].<br>TNF-α-APE: $\beta = -0.171$, SE = 0.063, $p < .01$, 95% CI [−0.295, −0.048].<br><br>Mediation analysis indicated that lower APE scores in survivors versus controls were partly explained by elevated IL-6: the indirect effect of group on	Self-reported impairment was significantly greater for survivors than controls in all models (except IL-10), but this effect was not mediated by immune markers and disappeared after adjusting for anxiety and depression.<br><br>Lower adjusted APE scores in survivors versus controls were mediated by elevated IL-10 (similar to IL-6), but this mediation was not statistically significant.<br><br>Survivors tended to have higher IL-8 and IL-10 than controls, but differences were not significant; no differences were observed for TNF-α or IFN-γ.<br><br>In the APE domain, there were trend-level mediated effects of IL-6 for Trail Making B and Digit Symbol.<br><br>For LM, no associations were found between survivor/control group and cognitive

			APE through IL-6 was significant ($\beta = -0.023$, SE = 0.009, $p = .01$, 95 % CI [−0.041, −0.005])	performance; thus, no significant mediation effects were observed.<br><br>Overall, multiple immune markers showed no or only limited associations with LM and self-reported cognition.
Myers et al., 2022 [34]	Pairwise correlations; mixed models	/	In the intervention group, IGF-1 levels increased significantly from baseline to 4 weeks and baseline to 16 weeks ($p < 0.01$, test statistic not reported, 95% CI not available; insufficient data).<br><br>Increases in IGF-1 were correlated with better neurocognitive performance on TMT-B ($r = 0.31$, $p = 0.02$, 95 % CI [0.05, 0.53]).	No between-group differences were observed for inflammatory biomarkers across the three assessment timepoints.<br><br>The association between IGF-1 and improvement in TMT-B performance was not significant in the mixed model.<br><br>No significant correlations were found between increases in IGF-1 and changes in self-reported cognitive function.
Ng et al., 2017* [36]	Generalized estimating equations (GEE)	Among patients with MCID-level self-perceived cognitive decline, BDNF changed significantly over time ($p = 0.029$), with a decline from assessment 1 to 3 ($p = 0.016$).<br><br>In non-impaired patients, BDNF also declined significantly from assessment 1 to 3 ($p < 0.001$).	/	No significant differences in BDNF were found between impaired and non-impaired groups at any time point.

		*Note: nonparametric tests do not provide CIs<br><br>After adjusting for fatigue, anxiety, depression, age, BMI, and genotype, higher plasma BDNF was significantly associated with greater self-perceived concentration deficits over time (GEE model, coefficient = −0.00003 per pg/mL, SE ≈ 0.00002, p = 0.032, 95% CI [−0.00006, −0.000003]).		
Palesh et al., 2025 [38]	Growth mixture modeling (GMM); multinomial logistic regression	/	/	Three NDE trajectory groups were identified: "high spikers" (class 1), "steady average" (class 2), and "steady low" (class 3).<br><br>No group × time effects were observed in any model.<br><br>No significant differences were found in cognitive subclass frequencies between NDE groups.<br><br>No significant main effects of NDE class or NDE class × time interactions on cognitive performance.
Pang et al., 2021 [39]	Correlations	/	Before and after the CALM intervention (and in the care-as-usual group),	/

			cytokine levels correlated significantly with MMSE scores:<br>IL-1β (*r* = -0.343 / -0.538, $p < 0.05$, 95 % CI [−0.57, −0.06] / [−0.73, −0.28])<br>TNF-*α* (*r* = -0.375 / -0.330, $p < 0.05$, 95 % CI [−0.60, −0.09] / [−0.56, −0.05])<br>IL-4 (r = 0.310 / -0.541 / -0.813, $p < 0.05$, 95 % CI [0.03, 0.55] / [−0.73, −0.28] / [−0.90, −0.62])	
Pang et al., 2023 [40]	Multiple regression model; correlation analysis	/	Psychological distress had a significant negative indirect effect on MMSE via IL-1β (*β* = -0.34, 95% CI [-0.59, -0.17]), TNF-*α* (*β* = -0.32, 95% CI [-0.52, -0.16]), and IL-4 (*β* = -0.32, 95% CI [-0.54, -0.13]).<br><br>The direct effect of distress on cognitive function remained significant (*β* = -1.31 to -1.33, $p < 0.05$, 95% CI not available; insufficient data) but was attenuated compared with the total effect (*β* = -1.65, $p < 0.05$, 95% CI not available; insufficient data).	/
Patel et al., 2023 [42]	Longitudinal multivariate linear mixed models	/	/	No inflammatory markers were significantly associated with either objective or self-reported cognitive outcomes.
Toh et al., 2020 [43]	Mann-Whitney U	Cognitive trajectories (FACT-Cog): Patients were classified as "non-	/	At each blood draw, cytokine distributions overlapped substantially, and no

		impaired" (n = 76) or "cognitively impaired" (n = 52), with impaired subtypes: acute (n = 11), delayed (n = 20), intermittent (n = 5), and persistent (n = 16).<br><br>Non-impaired group: Significant longitudinal cytokine changes observed for GM-CSF (p = 0.005), TNF-α (p = 0.015), IL-4 (p = 0.011), IL-6 (p = 0.032), IL-8 (p = 0.045), and IL-10 (p = 0.025).<br><br>Post-hoc: TNF-α (assessment 3 vs 2, p = 0.011); IL-8 (3 vs 2, p = 0.012); IL-4 (3 vs 1, $p < 0.001$).<br><br>Acute impairment trajectory: Changes over time in TNF-α (p = 0.017).<br><br>Persistent impairment trajectory: Significant cytokine changes for GM-CSF (p = 0.013), IL-1β (p = 0.017), IL-4 ($p < 0.001$), IL-6 (p = 0.002), and IL-8 (p = 0.028).<br><br>Elevated pro-inflammatory cytokines (IL-1β, IL-6, IL-8).		cytokine distinguished impaired from non-impaired women after correction.<br><br>Cytokine levels in the persistent impairment trajectory did not differ significantly from those in the non-impaired group.

		Post-hoc: IL-6 (2 vs 1, *p* = 0.013; 3 vs 1, *p* = 0.002), IL-4 (3 vs 2, *p* = 0.010; 3 vs 1, *p* = 0.002).<br>*Note: nonparametric tests do not provide CIs		
Vardy et al., 2019* [44]	Kruskal-Wallis test; and χ2 test; Wilcoxon rank sum test; Spearman rank sum correlation	/	IL-8 levels were modestly correlated with worse objective cognitive performance (clinical Global Deficit Score: ρ = 0.27, *p* = 0.03, 95% CI not available; insufficient data).	/
Von Ah et al., 2022 [45]	Correlations	/	/	Serum BDNF did not differ between groups.<br><br>BDNF levels showed no significant correlations with cognitive performance at baseline or post-intervention.
Yao et al., 2022 [47]	Correlations	CALM group: PIV and MLR were negatively correlated with FACT-Cog scores (*r* = -0.297, *p* = 0.021, 95% CI [–0.63, 0.09]; *r* = -0.264, *p* = 0.038, 95% CI [–0.61, 0.12]).<br><br>Control group: NLR and GLR were significantly upregulated (*z* = 2.618, *p* = 0.023, 95% CI not available; insufficient data; *z* = 2.212, *p* = 0.027, 95% CI not available; insufficient data).	/	/

Yap et al., 2020* [49]	multivariable logistic regression models	Plasma BDNF decreased from baseline (assessment 1) to post-ChT (assessment 3) in patients with and without CRCI, as well as in those with persistent CRCI and those without CRCI at assessment 4 (all $p < 0.01$). The decline was larger in patients impaired at assessment 3 ($p = 0.021$).<br><br>At assessment 3, a smaller decline in BDNF was associated with lower odds of clinically meaningful subjective CRCI (FACT-Cog decline ≥ 10.6; OR = 0.88 per ng/mL, $p = 0.041$, 95% CI [0.79, 0.99]).<br><br>Higher absolute BDNF at assessment 3 predicted reduced risk of persistent overall CRCI (OR = 0.74 per ng/mL, $p = 0.027$, 95% CI [0.57, 0.97]), persistent functional-interference CRCI (OR = 0.62, $p = 0.040$, 95% CI [0.39, 0.98]), and delayed functional-interference CRCI (OR = 0.55, $p =$	/	No statistically significant reduction in plasma BDNF from baseline (assessment 1) to post-ChT (assessment 3) was observed in participants with delayed CRCI.

		0.050, 95% CI [0.31, 1.00]).		
Yap et al., 2021 [50]	Friedman test; Wilcoxon signed-rank test with Bonferroni correction; linear mixed model analyses	/	/	No significant interactions were found between individual cytokines and cognitive impairment status when comparing patients who reported CRCI during follow-up with those who did not.<br><br>Interactions between other impairment trajectories and cytokines, including BDNF, were also non-significant.
Yu et al., 2022 [51]	Correlations	After chemotherapy, patients with cognitive impairment showed higher NLR (z = -2.444, p = 0.015) and CA153 (z = -2.293, p = 0.022) compared to those without impairment.<br><br>*Note: nonparametric tests do not provide CIs<br><br>NLR correlated significantly with RM, PM, and FACT-Cog scores (r = 0.173, 0.197, 0.214; all $p < 0.05$).<br><br>FACT-Cog CogPCA scores were significantly correlated with NLR (r = 0.164, p <	MMSE was significantly negatively correlated with NLR (r = -0.404, p < 0.001, 95% CI [−0.55, −0.23]), CEA (r = -0.205, p < 0.001, 95% CI [−0.38, −0.02]), and CA153 (r = -0.322; p < 0.001, 95% CI [−0.48, −0.14]).	/

		0.05), CEA (r = 0.160, $p < 0.05$), and CA153 (r = 0.255; $p < 0.05$). *Note: 95% CI not available; insufficient data (sample size and correlation type not specified)		
Zhao et al., 2020 [52]	Bivariate correlations	/	Cognition was significantly negatively correlated with IL-1β (r = -0.681, $p < 0.05$, 95% CI [−0.751, −0.596]), TNF-α (r = -0.572, $p < 0.05$, 95% CI [−0.661, −0.468]), and IL-4 (r = -0.626, $p < 0.05$, 95% CI [−0.705, −0.531]).<br><br>Post-ChT patients with cognitive impairment showed higher IL-1β (z = -4.353, $p < 0.05$), TNF-α (z = -3.383, $p < 0.05$), and IL-4 (z = -2.522, $p < 0.05$) compared to post-ChT patients without impairment.<br><br>Compared to pre-ChT patients, post-ChT patients with cognitive impairment had significantly higher IL-1β (z = -4.743, $p < 0.01$), TNF-α (z = -3.672, $p < 0.01$), and IL-4 (z = -2.954, $p < 0.01$).<br><br>*Note: nonparametric tests do not provide CIs	No significant differences in IL-1β (Z = -0.515, p > 0.05), TNF-α (Z = -0.256, p > 0.05), or IL-4 (Z = -0.185, p > 0.05) were observed between pre-ChT patients and post-ChT patients without cognitive impairment.
Zuniga et al., 2018 [53]	ANCOVA; multiple linear regression	Participants were grouped by carotenoid levels.	/	/

		A significant group effect was found for FACT-Cog scores ($F_{(3,60)} = 9.498$, $p < 0.001$, partial $\eta^2 = 0.322$).<br><br>Low-carotenoid BC patients reported significantly lower FACT-Cog scores (more complaints) compared to low-carotenoid healthy controls (Mdiff = -43.0, p < 0.001, 95% CI [-68.8, -17.1]) and high-carotenoid healthy controls (Mdiff = -44.54, p < 0.001, 95% CI [-71.1, -18.0]).		

Table S7. Results on biochemical biomarkers. The table shows main results of each study on the relationships between genetic biomarkers and self-reported cognitive assessment and objective cognitive assessment, separately. Main negative results are also reported as well as the relevant statistic of interest. 95% confidence intervals (CIs) were reported by the original studies or calculated by the authors when possible; CIs are not applicable for nonparametric rank tests (Friedman/Wilcoxon), and not computed when models reported only χ^2 and p without coefficients/SEs.

**Author(s), year**	**Relevant statistics**	**Main results on genetic biomarkers and self-reported cognition**	**Main results on genetic biomarkers and objective cognition**	**Null findings**
Bender et al., 2018 [4]	Group-based trajectory modeling; multinomial logistic regression; bivariate analysis; chi-square analyses	/	**DNA repair genes** associated with differential executive function subgroup membership**:**<br><br>PARP1 rs2271347 minor A allele (low vs. moderate OR = 3.87, p = 0.005, 95% CI	/

			[1.49, 10.00]), ERCC3 rs4150402 minor A Allele (low vs. moderate OR = 3.22, *p* = 0.025, 95% CI [1.16, 8.97]), and ERCC5 rs751402 minor T (low vs high: OR = 0.19, *p* = 0.011, 95% CI [0.05, 0.69]; low vs moderate OR = 0.29, *p* = 0.020, 95% CI [0.10, 0.82])<br><br>**Oxidative stress gene:**<br><br>GPX1 rs1050450 minor A allele associated with concentration subgroup membership (high vs. moderate OR = 6.27, *p* = 0.014, 95% CI [1.44, 27.28]).<br><br>**DNA repair gene (additional):**<br><br>ERCC3 rs4150407 minor G allele associated with concentration subgroup membership (low vs. moderate OR = 7.76, *p* = 0.023, 95% CI [1.33, 45.22]).	
Bower et al., 2013 [6]	Multivariate regression; logistic regression	/	/	Memory complaints were marginally worse in patients with

				the TNF-308 GG genotype.
Carrol et al., 2019* [9]	Linear regression models	/	High DNA damage was associated with lower executive function (β = -0.23, *p* = 0.027, 95% CI ≈ [−0.44, −0.03]) and showed a trend toward poorer memory (*p* = 0.06, full statistic not reported).<br><br>Lower telomerase activity predicted worse attention (*β* = 0.30, *p* = 0.006, 95% CI ≈ [0.09, 0.51]), executive function (β = 0.30, *p* = 0.002, 95% CI ≈ [0.11, 0.49]), and motor speed (β = 0.24, *p* = 0.037, 95% CI ≈ [0.01, 0.47]).	No biomarker (DNA damage, telomerase activity, or telomere length) significantly predicted self-reported cognitive complaints after covariate adjustment.<br><br>Telomere length was not associated with any objectively measured cognitive domain.
Chae et al., 2016* [10]	Logistic regression; generalized estimating equation model	/	/	No significant associations were found between IL6-174 or TNF-308 genotypes and risk of either self-perceived or objective cognitive impairment.
Chae et al., 2018 [11]	Multiple logistic regression	/	/	mtDNA reduction was not a significant predictor of either self-perceived CRCI or objective CRCI.
Chan et al., 2019 [12]	Binary logistic regression analysis;	The A allele of DNMT1 rs2162560 was associated with lower odds of self-	/	No significant demographic or genotype

	univariate analysis	perceived concentration difficulties (OR = 0.45, *p* = 0.01, 95% CI [0.25, 0.82]) and functional interference (OR = 0.48, *p* = 0.03, 95% CI [0.24, 0.95]).<br><br>In patients ≤ 51 years, the A allele was protective against declines in self-perceived memory (OR = 0.26, *p* = 0.01, 95% CI [0.09, 0.71]), concentration (OR = 0.30, *p* = 0.01, 95% CI [0.12, 0.74]), and mental acuity (OR = 0.42, p = 0.04, 95% CI [0.18, 0.96]).		differences were found between patients assessed with Headminder versus CANTAB.<br><br>DNMT1 rs2162560 genotype was not significantly associated with other FACT-Cog cognitive domains or with objective cognitive impairment.
Cheng et al., 2016 [14]	Logistic and linear regressions	/	COMT rs165599: GA genotype: lower odds of cognitive decline vs. GG (OR = 0.515, *p* = 0.048, 95% CI [0.27, 0.98].<br>AA genotype: lower odds vs. GG (OR = 0.318, *p* = 0.048, 95% CI [0.14, 0.74]).<br>Additive model: rs165599 increased CRCI risk (OR = 0.556, *p* = 0.037, 95% CI [0.37, 0.85]); no effects in dominant or recessive models.<br><br>COMT rs737865: AG genotype: significantly different from GG (OR = 0.995, *p* = 0.049, 95% CI [0.56, 1.76]).	COMT rs737865: No significant associations were found with cognitive outcomes under recessive, dominant, or additive models.<br>APOE (rs429358, rs7412): No significant differences between groups.<br><br>BDNF (rs6265): No significant differences between groups.

			GG genotype: lower odds vs. reference (OR = 0.157, $p = 0.049$, 95% CI [0.04, 0.71]).	
Cho et al., 2024 [15]	Multiple linear regression	CpG site cg20108357: Strongest association with PAOFI total score ($\beta = -0.33$, $p < 0.001$, 95% CI [−1.95, −0.54]); greater methylation linked to better perceived cognitive function.<br><br>CpG site cg00567892: Negatively associated with all PAOFI scores except the language/communication domain ($\beta = -0.23$, $p = 0.019$, 95% CI [−3.25, −0.30]); greater methylation linked to better perceived cognitive function across most domains.	BDNF gene (88 CpG sites): Only cg21291635 was significantly associated with processing speed ($\beta = 0.25$, $p = 0.011$, 95% CI [0.06, 0.44]); greater methylation linked to poorer processing speed.<br><br>RASA2 gene (37 CpG sites): Only cg20247102 was significantly associated with processing speed ($\beta = 0.23$, $p = 0.020$, 95% CI [0.04, 0.42]); greater methylation linked to poorer processing speed.	BDNF M-value: Not associated with processing speed or PAOFI total score.<br><br>RASA2 M-value: Not associated with processing speed or PAOFI total score.
Conroy et al., 2013 [16]	Linear regression	/	/	No significant correlations were observed between oxidative DNA damage and cognitive scores.
Harrison et al., 2021 [20]	t-test; Wilcoxon rank tests	/	Processing speed performance was significantly lower in ChT-treated survivors carrying the APOE ε4 allele ($p < 0.05$; exact test statistic not reported).	/

Koleck et al., 2014 [27]	Multiple linear regression analysis	/	APOE ε4 allele: Carriers had poorer verbal learning and memory at assessment 1 (β = -0.334, p = 0.031, 95% CI ≈ [−0.637, −0.031]) and assessment 2 (β = -0.3222, p = 0.038, 95% CI ≈ [−0.627, −0.018]), independent of cancer or treatment status. Carriers showed a greater decline in visual learning and memory from assessment 1 to 2 (β = -0.269, p = 0.027, 95% CI ≈ [−0.507, −0.031]).<br><br>APOE ε4 × Anastrozole: Negatively impacted executive function at assessment 1 (β = -0.4448, p = 0.088, 95% CI ≈ [−0.956, 0.066]) and assessment 2 (β = -0.5771, p = 0.033, 95% CI ≈ [−1.108, −0.047]). Negatively impacted change in visual learning and memory from assessment 1 to 3 (β = -0.567, p = 0.042, 95% CI ≈ [−1.114, −0.020]). Negatively impacted change in attention from	No significant associations were observed for APOE ε4 genotype status (χ^2 = 1.192, p = 0.551).

			assessment 2 to 3 (β = -0.5715, p = 0.045, 95% CI ≈ [−1.130, −0.013]).<br><br>APOE ε4 × ChT + Anastrozole: Positively impacted verbal learning and memory from assessment 1 to 3 (β = 0.5468, p = 0.064; trend-level, 95% CI ≈ [−0.032, 1.125]).	
Koleck et al ., 2017 [28]	Multiple linear regression modeling; Genetic risk/protection scores	/	Candidate genes: Every candidate gene except CMC2, MMP11, and RACGAP1 harbored at least one polymorphism whose minor allele (or interaction with treatment) significantly ($p < 0.05$) influenced performance in one or more cognitive domains.<br><br>Attention: MIR125A (rs12976445), ESR1 (rs2347867, rs3020314, rs6557171, rs985694), MYBL2 (rs2070235), SCUBE2 (rs6486125). Genetic risk/protection score positively associated with	/

			attention (β = 0.47, p < 0.001, ΔR^2 = 0.066, 95% CI not available; insufficient data).<br><br>Concentration: AURKA, BCL2, CCNB1, CENPA, DIAPH3, ESR1 (+ CCDC170), GRB7, MELK, PGR.<br>Genetic score: β = 0.54, p < 0.001, ΔR^2 = 0.189, 95% CI not available; insufficient data.<br><br>Executive function: BAG1, BCL2, CCNB1, CTSL2, DIAPH3, ESR1, GSTM1, MELK, MYBL2, PGR, SCUBE2.<br>Genetic score: β = 0.36, p < 0.001, ΔR^2 = 0.204, 95% CI not available; insufficient data.<br><br>Mental flexibility: BCL2, DIAPH3, ESR1 (+ CCDC170), MIR125A, NFE2L2, MKI67, MYBL2, NDC80, RFC4, RRM2, SCUBE2.<br>Genetic score: β = 0.54, p < 0.001, ΔR^2 = 0.224, 95% CI not available; insufficient data.<br><br>Psychomotor speed: BCL2,	

			CENPA, ESR1, MKI67, PGR. Strongest effect: $\beta = 0.67$, $p < 0.001$, $\Delta R^2 = 0.093$, 95% CI not available; insufficient data.<br><br>Verbal memory: AURKA, BCL2, BIRC5, CCNB1, CD68, CENPA, CTSL2, DIAPH3, ESR1 (+ CCDC170), GSTM1, MYBL2, NDC80, ORC6, PGR. Genetic score: $\beta = 0.34$, $p < 0.001$, $\Delta R^2 = 0.209$, 95% CI not available; insufficient data.<br><br>Visual memory: BAG1, BCL2, CCNB1, DIAPH3, ESR1, GSTM1, MYBL2, PGR, RRM2. Genetic score: $\beta = 0.75$, $p < 0.001$, $\Delta R^2 = 0.148$, 95% CI not available; insufficient data.<br><br>Visual working memory: AURKA, BAG1, BIRC5, CCNB1, CD68, DIAPH3, ESR1, GRB7, GSTM1, MELK, MYBL2, PGR. Genetic score: $\beta = 0.42$, $p < 0.001$, $\Delta R^2 = 0.241$, 95% CI not available; insufficient data.	

Koleck, et al., 2016 [29]	Hierarchical multiple linear regression; genetic risk scores	/	Attention: ERCC3 rs2134794 (β = -0.309, *p* = 0.010, 95% CI not available; insufficient data) and ERCC5 rs873601 (β = -0.288, *p* = 0.015, 95% CI not available; insufficient data) minor alleles associated with poorer performance.<br><br>Mental flexibility: SNP main effects across oxidative stress/DNA repair genes: ERCC2 rs13181 (β = -0.179, *p* = 0.031, 95% CI not available; insufficient data), ERCC3 rs4150407 (β = 0.234, *p* = 0.016, 95% CI not available; insufficient data), ERCC3 rs4150477 (β = 0.190, *p* = 0.038, 95% CI not available; insufficient data), PARP1 rs2271347 (β = 0.202, *p* = 0.034, 95% CI not available; insufficient data), SEPP1 rs230819 (β = 0.255, *p* = 0.018, 95% CI not available; insufficient data), SOD1 rs1041740 (β = 0.254, *p* = 0.006, 95% CI not	/

			available; insufficient data).<br><br>Psychomotor speed:<br>SNP main effects: CAT rs511895 (β = 0.237, p = 0.031, 95% CI not available; insufficient data), CAT rs769214 (β = -0.421, p = 0.020, 95% CI not available; insufficient data), ERCC5 rs11069498 (β = -0.236, p = 0.044, 95% CI not available; insufficient data), ERCC5 rs751402 (β = -0.224, p = 0.050, 95% CI not available; insufficient data), ERCC5 rs873601 (β = -0.227, p = 0.037, 95% CI not available; insufficient data), SEPP1 rs3877899 (β = -0.327, p = 0.005, 95% CI not available; insufficient data).<br>Interaction: Anastrozole-only × ERCC5 rs2296148 (β = 1.075, p = 0.024, 95% CI not available; insufficient data) and SOD1 rs1041740 (β = -0.619, p = 0.015, 95% CI not	

			available; insufficient data).<br><br>Concentration: SOD2 polymorphisms: rs4880 (β = -0.303, p = 0.024, 95% CI not available; insufficient data), rs5746136 (β = -0.257, p = 0.023, 95% CI not available; insufficient data), rs8031 (β = -0.332, p = 0.011, 95% CI not available; insufficient data). Interaction: Anastrozole-only × ERCC2 rs3916874 (β = 0.533, p = 0.050, 95% CI not available; insufficient data), ERCC2 rs50872 (β = -0.882, p = 0.001, 95% CI not available; insufficient data), ERCC3 rs4150407 (β = 0.546, p = 0.047, 95% CI not available; insufficient data), ERCC5 rs2296147 (β = 0.585, p = 0.043, 95% CI not available; insufficient data).<br><br>Executive function: Interaction: ChT + Anastrozole × DNA repair variants: ERCC3 rs2134794 (β =	

			0.470, p = 0.023, 95% CI not available; insufficient data), ERCC3 rs4150407 (β = -0.466, p = 0.035, 95% CI not available; insufficient data), ERCC3 rs4150477 (β = -0.417, p = 0.046, 95% CI not available; insufficient data), ERCC5 rs2296147 (β = 0.477, p = 0.034, 95% CI not available; insufficient data), PARP1 rs2271347 (β = -0.589, p = 0.006, 95% CI not available; insufficient data).<br><br>Verbal memory: Interaction: ChT + Anastrozole × ERCC5 rs11069498 (β = 0.536, p = 0.034, 95% CI not available; insufficient data), ERCC5 rs4150360 (β = 0.568, p = 0.031, 95% CI not available; insufficient data), ERCC5 rs751402 (β = 0.486, p = 0.038, 95% CI not available; insufficient data).<br><br>Visual working memory: Interaction: ChT + Anastrozole × ERCC5	

			rs11069498 (β = 0.629, p = 0.027, 95% CI not available; insufficient data), ERCC5 rs4150360 (β = 0.673, p = 0.023, 95% CI not available; insufficient data).<br><br>Visual memory: Interaction: ChT + Anastrozole × ERCC5 rs751402 (β = 0.499, p = 0.023, 95% CI not available; insufficient data), CAT rs1001179 (β = -0.512, p = 0.032, 95% CI not available; insufficient data), CAT rs769214 (β = 0.480, p = 0.024, 95% CI not available; insufficient data). SNP main effects: CAT rs525938 (β = -0.282, p = 0.049, 95% CI not available; insufficient data), CAT rs566979 (β = -0.282, p = 0.049, 95% CI not available; insufficient data).	
Li et al., 2020 [30]	Logistic regression; general genetic model; linear regression	/	COMT rs737865: A/A genotype associated with higher EBPM scores (β = 1.536, p = 0.040, 95% CI [1.02, 2.313]). Recessive model showed a slight association with	/

			EBPM (β = 1.499, p = 0.041, 95% CI [1.017, 2.211]).<br><br>G/G genotype linked to lower probability of CRCI (OR = 0.519, p = 0.047, 95% CI [0.272, 0.991]). Recessive model significantly increased CRCI risk (OR = 2.888, p = 0.032, 95% CI [1.096, 7.612]).<br><br>COMT rs165599: G/G genotype associated with greater likelihood of cognitive decline compared to G/A and A/A (OR = 2.019, p = 0.024, 95% CI [1.097-3.717]).	
Ng et al., 2016 [35]	Binary logistic regression	BDNF Val66Met (rs6265): Met/Met vs. Val/Val: lower odds of self-perceived impairment (OR = 0.26, p = 0.036, 95% CI [0.08, 0.92]).<br><br>Domain-specific findings:<br>Verbal fluency: Met carriers vs. Val/Val (OR = 0.34, p = 0.031, 95% CI [0.12-0.90]).<br><br>Multitasking ability: Met carriers vs. Val/Val (OR = 0.37, p = 0.030, 95% CI [0.15, 0.91]).<br><br>Older subgroup (≥55 years, n = 54):<br>Global FACT-Cog decline: Met carriers vs.	/	No significant associations were found between BDNF Val66Met genotype and impairment in any objectively measured cognitive domain.

		Val/Val (OR = 0.07, p = 0.035, 95% CI [0.01-0.83]).<br><br>Val/Met vs. Val/Val: OR = 0.06, p = 0.031, 95% CI [0.01, 0.77].<br><br>Multitasking: Val/Met vs. Val/Val (OR = 0.09, p = 0.040, 95% CI [0.01, 0.90]).		
Ng et al., 2017* [36]	Generalized estimating equations	Patients with the Met/Met genotype had significantly lower odds of cognitive decline compared to Val/Val (OR = 0.26, p = 0.036, 95% CI [0.08, 0.92]).<br><br>The Met allele was associated with greater protection against overall self-perceived impairment (p = 0.041, full statistic not reported) and concentration deficit (p = 0.043, full statistic not reported).	/	/
Nudelman et al., 2023 [37]	Linear regressions; general linear models	/	GWAS findings: Two loci showed genome-wide significant SNP × diagnosis interactions: Chromosome 1 rs76859653 (F = 32.68, p < .001, partial η^2 = .048), Adjusted APE means (95% CI): Controls, noncarriers = 0.10 [0.06, 0.15]; carriers = 0.71 [0.46, 0.97]; BC, noncarriers = 0.08 [0.03, 0.12]; carriers = −0.56 [−0.91, −0.22]. Chromosome 2 rs78786199 (F =	Gene-level analysis did not identify any genes enriched for variants significantly associated with attention, processing speed, or executive function 1-year scores (controlling for baseline) when comparing cancer patients with controls.

			32.27, $p < .001$, partial $\eta^2 = .047$), Adjusted APE means (95% CI): Controls, noncarriers = 0.12 [0.07, 0.16]; carriers = 0.27 [0.09, 0.45]; BC, noncarriers = 0.08 [0.04, 0.13]; carriers = −0.77 [−1.06, −0.48].<br><br>Controls: Minor allele carriers had similar or greater attention, processing speed, and executive function at 1-year follow-up vs. non-carriers.<br><br>BC cases: Minor allele carriers had lower attention, processing speed, and executive function at 1-year follow-up vs. non-carriers.<br><br>Gene-based analysis: POC5 (centriolar protein) significantly enriched for variants associated with LM domain performance differences ($p < .001$).	
Park et al., 2025 [41]	Linear mixed models	COMT rs4680: GG genotype associated with the most favorable improvement in CogTot ($p < .05$, Cohen's $d \approx 0.3$–	DRD2 rs6277: GG genotype associated with decreases in visuospatial	/

		0.5, full statistics not reported).<br><br>DRD2 rs6277: GG genotype associated with greater improvement in perceived cognitive functioning ($p < .05$; Cohen's $d \approx 0.3–0.4$, full statistics not reported).	memory (BVMT-DR, BVMT-TR), verbal memory (HVLT-DR), and executive functioning ($p < 0.05$; full statistics not reported).	
Vardy et al., 2019* [44]	Kruskal-Wallis test; and χ2 test; Wilcoxon rank sum test; Spearman rank sum correlation	/	/	No significant differences in GDS (clinical or CANTAB), cognitive impairment rates, or FACT-Cog scores were observed between carriers and non-carriers of the APOE ε4 allele.
Yang et al., 2020 [46]	Stepwise linear regression	/	Epigenetics - Memory domain:<br><br>Memory was the only domain with significant epigenetic correlates; 56 CpG sites remained significant after covariate adjustment (overall model $p = 0.0049$).<br><br>Greater methylation change predicted larger declines (or smaller gains) in standardized memory scores (genes: USP6NL, UBE2V1, RIPOR2, ECE2, PPFIBP2,	Epigenetics - Other domains: no CpG sites significantly predicted psychomotor speed, reaction time, complex attention, or cognitive flexibility after covariate adjustment (all models non-significant).

			DDHD1, KLF5, HSD17B3, DGKA, RPS6KA1; $r < 0$, $p < .001$, 95% CI not available; insufficient data).	
Yao et al., 2023 [48]	Independent-samples t- test; Mann- Whitney U- test	/	Polymorphisms associated with greater risk of cognitive impairment during ChT: ALDH2 rs671_GG: impaired DST ($z = 2.769$, $p = 0.006$, 95% CI not available; insufficient data) and VFT ($t = 4.624$, $p < 0.001$, 95% CI not available; insufficient data).<br><br>rs886205_GG: impaired MMSE ($z = 2.405$, $p < 0.05$, 95% CI not available; insufficient data), DST ($z = 3.663$, $p < 0.001$, 95% CI not available; insufficient data), VFT ($z = 4.709$, $p < 0.001$, 95% CI not available; insufficient data).<br><br>rs4648328_CC: impaired DST ($z = 2.850$, $p = 0.004$, 95% CI not available; insufficient data), VFT ($t = 3.477$, $p = 0.001$, 95% CI not	/

			available; insufficient data).<br><br>rs4767944_TT: impaired DST (z = 2.967, p = 0.003, 95% CI not available; insufficient data), VFT (t = 2.776, p = 0.008, 95% CI not available; insufficient data).<br><br>Cognitive performance significantly declined after ChT in these genotypes ($p < 0.05$, 95% CI not available; insufficient data).<br><br>The effect of ALDH2 rs671 was the most pronounced.	
Yap et al., 2020* [49]	Multivariable logistic regression models	Plasma BDNF:<br>At end of ChT, smaller reduction from baseline to T3 was protective against overall subjective CRCI (OR = 0.88, p = 0.041, 95% CI [0.79, 0.99]). Longitudinally, higher post-ChT BDNF was protective against persistent subjective CRCI (OR = 0.74, p = 0.027, 95% CI [0.57, 0.97]) and functional interference (OR = 0.62, p = 0.040, 95% CI [0.39, 0.98]).<br><br>BDNF Met allele:<br>Reduced odds of subjective CRCI in multitasking (OR = 0.30, p		No significant associations were found between cognitive outcomes and COMT rs737865, APOE rs429358/rs7412, or BDNF rs6265.

		= 0.007, 95% CI [0.12, 0.71]) and memory (OR = 0.27, *p* = 0.016, 95% CI [0.10, 0.79]) at end of ChT. Reduced odds of persistent subjective CRCI in mental acuity (OR = 0.18, *p* = 0.027, 95% CI [0.04, 0.83]) and multitasking (OR = 0.06, p = 0.007, 95% CI [0.008, 0.46]).		

*Note: studies with asterisk investigated both biochemical and genetic biomarkers.

References

1. Andreano, J.M. and W., J. and Donley, L. and Cahill, L. Effects of Breast Cancer Treatment on the Hormonal and Cognitive Consequences of Acute Stress. *Psycho-Oncology* **2012**, *21*, 1091–1098, doi:10.1002/pon.2006.
2. Aspelund, S.G.; Halldorsdottir, T.; Agustsson, G.; Sigurdardottir Tobin, H.R.; Wu, L.M.; Amidi, A.; Johannsdottir, K.R.; Lutgendorf, S.K.; Telles, R.; Daly, H.F.; et al. Biological and Psychological Predictors of Cognitive Function in Breast Cancer Patients before Surgery. *Support Care Cancer* **2024**, *32*, 88, doi:10.1007/s00520-023-08282-5.
3. Belcher, E.K. and C., E. and Gilmore, N.J. and Hardy, S.J. and Kleckner, A.S. and Kleckner, I.R. and Lei, L. and Heckler, C. and Sohn, M.B. and Thompson, B.D. and Lotta, L.T. and Werner, Z.A. and Geer, J. and Hopkins, J.O. and Corso, S.W. and Rich, D.Q. and Van Wijngaarden, E. and Janelsins, M.C. Inflammation, Attention, and Processing Speed in Patients with Breast Cancer before and after Chemotherapy. *Journal of the National Cancer Institute* **2022**, *114*, 712–721, doi:10.1093/jnci/djac022.
4. Bender, C.M. and M., John D. and Sereika, Susan M. and Gentry, Amanda L. and Casillo, Frances E. and Koleck, Theresa A. and Rosenzweig, Margaret Q. and Brufsky, Adam M. and McAuliffe, Priscilla and Zhu, Yehui and Conley, Yvette P. Trajectories of Cognitive Function and Associated Phenotypic and Genotypic Factors in Breast Cancer. *ONCOLOGY NURSING FORUM* **2018**, *45*, 308–326, doi:10.1188/18.ONF.308-326.
5. Boivin, M.J. and A., G.P. and Felt, N.G. and Shamoun, L. Preliminary Study on the Effects of Treatment for Breast Cancer: Immunological Markers as They Relate to Quality of Life and Neuropsychological Performance. *BMC Women's Health* **2020**, *20*, doi:10.1186/s12905-020-00971-1.
6. Bower, J.E. and G., P.A. and Irwin, M.R. and Castellon, S. and Arevalo, J. and Cole, S.W. Cytokine Genetic Variations and Fatigue among Patients with Breast Cancer. *Journal of Clinical Oncology* **2013**, *31*, 1656–1661, doi:10.1200/JCO.2012.46.2143.
7. Boyle, C.C. and G., P.A. and Van Dyk, K.M. and Bower, J.E. Inflammation and Attentional Bias in Breast Cancer Survivors. *Brain, Behavior, and Immunity* **2017**, *66*, 85–88, doi:10.1016/j.bbi.2017.05.016.
8. Carlson, B.W.; Craft, M.A.; Carlson, J.R.; Razaq, W.; Deardeuff, K.K.; Benbrook, D.M. Accelerated Vascular Aging and Persistent Cognitive Impairment in Older Female Breast Cancer Survivors. *Geroscience* **2018**, *40*, 325–336, doi:10.1007/s11357-018-0025-z.
9. Carroll, J.E. and S., B.J. and Tometich, D.B. and Zhai, W. and Zhou, X. and Luta, G. and Ahles, T.A. and Saykin, A.J. and Nudelman, K.N.H. and Clapp, J.D. and Jim, H.S. and Jacobsen, P.B. and Hurria, A. and Graham, D. and McDonald, B.C. and Denduluri, N. and Extermann, M. and Isaacs, C. and Dilawari, A.A. and Root, J. and Stern, R.A. and Mandelblatt, J.S. Sleep Disturbance and Neurocognitive Outcomes in Older Patients with Breast Cancer: Interaction with Genotype. *Cancer* **2019**, *125*, 4516–4524, doi:10.1002/cncr.32489.
10. Chae, J.-W. and N., T. and Yeo, H.L. and Shwe, M. and Gan, Y.X. and Ho, H.K. and Chan, A. Impact of TNF-α (Rs1800629) and IL-6 (Rs1800795) Polymorphisms on Cognitive Impairment in Asian Breast Cancer Patients. *PLoS ONE* **2016**, *11*, doi:10.1371/journal.pone.0164204.

11. Chae, J.-W.; Chua, P.S.; Ng, T.; Yeo, A.H.L.; Shwe, M.; Gan, Y.X.; Dorajoo, S.; Foo, K.M.; Loh, K.W.-J.; Koo, S.-L.; et al. Association of Mitochondrial DNA Content in Peripheral Blood with Cancer-Related Fatigue and Chemotherapy-Related Cognitive Impairment in Early-Stage Breast Cancer Patients: A Prospective Cohort Study. *Breast Cancer Res Treat* **2018**, *168*, 713–721, doi:10.1007/s10549-017-4640-7.
12. Chan, A. and Y., Angie and Shwe, Maung and Tan, Chia Jie and Foo, Koon Mian and Chu, Pat and Khor, Chiea Chuen and Ho, Han Kiat An Evaluation of DNA Methyltransferase 1 (DNMT1) Single Nucleotide Polymorphisms and Chemotherapy-Associated Cognitive Impairment: A Prospective, Longitudinal Study. *Scientific reports* **2019**, *9*, 14570, doi:10.1038/s41598-019-51203-y.
13. Chen, V.C.-H. and L., C.K. and Hsiao, H.P. and Tzang, B.S. and Hsu, Y.H. and Wu, S.I. and Stewart, R. Effects of Cancer, Chemotherapy and Cytokines on Subjective and Objective Cognitive Functioning among Patients with Breast Cancer. *Cancers* **2021**, *13*, doi:10.3390/cancers13112576.
14. Cheng, H.; Li, W.; Gan, C.; Zhang, B.; Jia, Q.; Wang, K. The COMT (Rs165599) Gene Polymorphism Contributes to Chemotherapy-Induced Cognitive Impairment in Breast Cancer Patients. *Am J Transl Res* **2016**, *8*, 5087–5097.
15. Cho, M.; Sereika, S.M.; Cummings, M.; Erickson, K.I.; Bender, C.M.; Conley, Y.P. DNA Methylation of BDNF and RASA2 Genes Is Associated With Cognitive Function in Postmenopausal Women With Breast Cancer.; 2024; Vol. 51, p. 349.
16. Conroy, S.K. and M., B.C. and Smith, D.J. and Moser, L.R. and West, J.D. and Kamendulis, L.M. and Klaunig, J.E. and Champion, V.L. and Unverzagt, F.W. and Saykin, A.J. Alterations in Brain Structure and Function in Breast Cancer Survivors: Effect of Post-Chemotherapy Interval and Relation to Oxidative DNA Damage. *Breast Cancer Research and Treatment* **2013**, *137*, 493–502, doi:10.1007/s10549-012-2385-x.
17. Duivon, M. and L., J. and Di Meglio, A. and Pradon, C. and Vaz-Luis, I. and Martin, A.L. and Everhard, S. and Broutin, S. and Rigal, O. and Bousrih, C. and Lévy, C. and Lerebours, F. and Lange, M. and Joly, F. Inflammation at Diagnosis and Cognitive Impairment Two Years Later in Breast Cancer Patients from the Canto-Cog Study. *Breast Cancer Research* **2024**, *26*, 93, doi:10.1186/s13058-024-01850-5.
18. Gan, C. and Y., S. and Zhao, J. and Shi, H. and Xu, J. and Zhang, M. and Cheng, H. Expression of Inflammatory States in Response to Psychological Distress in Breast Cancer Survivors and Its Relationship to Subjective Memory Function Complaints. *BMC Women's Health* **2025**, *25*, doi:10.1186/s12905-025-03674-7.
19. Ganz, P.A. and B., J.E. and Kwan, L. and Castellon, S.A. and Silverman, D.H.S. and Geist, C. and Breen, E.C. and Irwin, M.R. and Cole, S.W. Does Tumor Necrosis Factor-Alpha (TNF-α) Play a Role in Post-Chemotherapy Cerebral Dysfunction? *Brain, Behavior, and Immunity* **2013**, *30*, S99–S108, doi:10.1016/j.bbi.2012.07.015.
20. Harrison, R.A. and R., Vikram and Kesler, Shelli R. The Association of Genetic Polymorphisms with Neuroconnectivity in Breast Cancer Patients. *SCIENTIFIC REPORTS* **2021**, *11*, doi:10.1038/s41598-021-85768-4.
21. Henneghan, A.M. and P., O. and Harrison, M. and Kesler, S.R. Identifying Cytokine Predictors of Cognitive Functioning in Breast Cancer Survivors up to 10years Post Chemotherapy Using Machine Learning. *Journal of Neuroimmunology* **2018**, *320*, 38–47, doi:10.1016/j.jneuroim.2018.04.012.
22. Henneghan, A. and W., Michelle L. and Bourne, Garrett and Sales, Adam C. A Cross-Sectional Exploration of Cytokine-Symptom Networks in Breast Cancer Survivors Using Network Analysis. *CANADIAN JOURNAL OF NURSING RESEARCH* **2021**, *53*, 303–315, doi:10.1177/0844562120927535.
23. Janelsins, M.C. and L., L. and Netherby-Winslow, C. and Kleckner, A.S. and Kerns, S. and Gilmore, N. and Belcher, E. and Thompson, B.D. and Werner, Z.A. and Hopkins, J.O. and Long, J. and Cole, S. and Culakova, E. Relationships between Cytokines and Cognitive Function from Pre- to Post-Chemotherapy in Patients with Breast Cancer. *Journal of Neuroimmunology* **2022**, *362*, doi:10.1016/j.jneuroim.2021.577769.
24. Jenkins, V. and T., Ryan and Cercignani, Mara and Sacre, Sandra and Harrison, Neil and Whiteley-Jones, Hefina and Mullen, Lisa and Chamberlain, Giselle and Davies, Kevin and Zammit, Charles and Matthews, Lucy and Harder, Helena A Feasibility Study Exploring the Role of Pre-Operative Assessment When Examining the Mechanism of `chemo-Brain' in Breast Cancer Patients. *SPRINGERPLUS* **2016**, *5*, doi:10.1186/s40064-016-2030-y.

25. Keetile, N. and O., Elzbieta and Lentoor, Antonio G. and Rasakanya, Tsakani Association of Circulating Levels of Inflammatory Cytokines and Chemotherapy-Associated Subjective Cognitive Impairment in a South African Cohort of Breast Cancer Patients. *NEUROSCI* **2023**, *4*, 296–304, doi:10.3390/neurosci4040024.
26. Kesler, S. and J., M. and Koovakkattu, D. and Palesh, O. and Mustian, K. and Morrow, G. and Dhabhar, F.S. Reduced Hippocampal Volume and Verbal Memory Performance Associated with Interleukin-6 and Tumor Necrosis Factor-Alpha Levels in Chemotherapy-Treated Breast Cancer Survivors. *Brain, Behavior, and Immunity* **2013**, *30*, S109–S116, doi:10.1016/j.bbi.2012.05.017.
27. Koleck, T.A. and B., Catherine M. and Sereika, Susan M. and Ahrendt, Gretchen and Jankowitz, Rachel C. and McGuire, Kandace P. and Ryan, Christopher M. and Conley, Yvette P. Apolipoprotein E Genotype and Cognitive Function in Postmenopausal Women With Early-Stage Breast Cancer. *ONCOLOGY NURSING FORUM* **2014**, *41*, E313–E325, doi:10.1188/14.ONF.E313-E325.
28. Koleck, T.A. and B., C.M. and Clark, B.Z. and Ryan, C.M. and Ghotkar, P. and Brufsky, A. and McAuliffe, P.F. and Rastogi, P. and Sereika, S.M. and Conley, Y.P. An Exploratory Study of Host Polymorphisms in Genes That Clinically Characterize Breast Cancer Tumors and Pretreatment Cognitive Performance in Breast Cancer Survivors. *Breast Cancer: Targets and Therapy* **2017**, *9*, 95–110, doi:10.2147/BCTT.S123785.
29. Koleck, T.A. and B., Catherine M. and Sereika, Susan M. and Brufsky, Adam M. and Lembersky, Barry C. and McAuliffe, Priscilla F. and Puhalla, Shannon L. and Rastogi, Priya and Conley, Yvette P. Polymorphisms in DNA Repair and Oxidative Stress Genes Associated with Pre-Treatment Cognitive Function in Breast Cancer Survivors: An Exploratory Study. *SPRINGERPLUS* **2016**, *5*, doi:10.1186/s40064-016-2061-4.
30. Li, W.; Zhao, J.; Ding, K.; Chao, H.H.; Li, C.-S.R.; Cheng, H.; Shen, L. Catechol-O-Methyltransferase Gene Polymorphisms and the Risk of Chemotherapy-Induced Prospective Memory Impairment in Breast Cancer Patients with Varying Tumor Hormonal Receptor Expression. *Med Sci Monit* **2020**, *26*, e923567-1-e923567-10, doi:10.12659/MSM.923567.
31. Lyon, D.E. and C., R. and Chen, H. and Kelly, D.L. and McCain, N.L. and Starkweather, A. and Ahn, H. and Sturgill, J. and Jackson-Cook, C.K. Relationship of Systemic Cytokine Concentrations to Cognitive Function over Two Years in Women with Early Stage Breast Cancer. *Journal of Neuroimmunology* **2016**, *301*, 74–82, doi:10.1016/j.jneuroim.2016.11.002.
32. Madison, A.A.; Andridge, R.; Renna, M.E.; Sheridan, J.F.; Lustberg, M.; Ramaswamy, B.; Wesolowski, R.; Williams, N.O.; Sardesai, S.D.; Noonan, A.M.; et al. Inflamed but Not Impulsive: Acute Inflammatory Cytokine Response Does Not Impact Prepotent Response Inhibition. *Journal of Affective Disorders* **2023**, *342*, 1–9, doi:10.1016/j.jad.2023.09.008.
33. Mandelblatt, J.S. and S., Brent J. and Zhou, Xingtao and Nakamura, Zev M. and Cohen, Harvey J. and Ahles, Tim A. and Ahn, Jaeil and Bethea, Traci N. and Extermann, Martine and Graham, Deena and Isaacs, Claudine and Jacobsen, Paul B. and Jim, Heather S.L. and McDonald, Brenna C. and Patel, Sunita K. and Rentscher, Kelly E. and Root, James C. and Saykin, Andrew J. and Tometich, Danielle B. and Van Dyk, Kathleen and Zhai, Wanting and Breen, Elizabeth C. and Carroll, Judith E. Plasma Levels of Interleukin-6 Mediate Neurocognitive Performance in Older Breast Cancer Survivors: The Thinking and Living With Cancer Study. *CANCER* **2023**, *129*, 2409–2421, doi:10.1002/cncr.34784.
34. Myers, J.S.; Pathak, H.B.; He, J.; Ghosh, A.; Puri, R.V.; Asakura, Y.; Miyashita, M. Combined Exercise and Game-Based Cognitive Training Intervention: Correlative Pilot Study of Neurotrophic and Inflammatory Biomarkers for Women with Breast Cancer. *Cancer Nurs* **2022**, *47*, 10.1097/NCC.0000000000001175, doi:10.1097/NCC.0000000000001175.
35. Ng, T. and T., S.M. and Yeo, H.L. and Shwe, M. and Gan, Y.X. and Cheung, Y.T. and Foo, K.M. and Cham, M.T. and Lee, J.A. and Tan, Y.P. and Fan, G. and Yong, W.S. and Preetha, M. and Loh, W.J.K. and Koo, S.L. and Jain, A. and Lee, G.E. and Wong, M. and Dent, R. and Yap, Y.S. and Ng, R. and Khor, C.C. and Ho, H.K. and Chan, A. Brain-Derived Neurotrophic Factor Genetic Polymorphism (Rs6265) Is Protective against Chemotherapy-Associated Cognitive Impairment in Patients with Early-Stage Breast Cancer. *Neuro-Oncology* **2016**, *18*, 244–251, doi:10.1093/neuonc/nov162.
36. Ng, T. and L., Y.Y. and Chae, J.W. and Yeo, A.H.L. and Shwe, M. and Gan, Y.X. and Ng, R.C.H. and Chu, P.P.Y. and Khor, C.C. and Ho, H.K. and Chan, A. Evaluation of Plasma Brain-Derived Neurotrophic

Factor Levels and Self-Perceived Cognitive Impairment Post-Chemotherapy: A Longitudinal Study. *BMC Cancer* **2017**, *17*, doi:10.1186/s12885-017-3861-9.
37. Nudelman, K. and N., Kwangsik and Zhang, Michael and McDonald, Brenna C. and Zhai, Wanting and Small, Brent J. and Wegel, Claire E. and Jacobsen, Paul B. and Jim, Heather S.L. and Patel, Sunita K. and Graham, Deena M.A. and Ahles, Tim A. and Root, James C. and Foroud, Tatiana and Breen, Elizabeth C. and Carroll, Judith E. and Mandelblatt, Jeanne S. and Saykin, Andrew J. Genetic Variants Associated with Longitudinal Cognitive Performance in Older Breast Cancer Patients and Controls †. *CANCERS* **2023**, *15*, doi:10.3390/cancers15112877.
38. Palesh, O. and B., S.E. and Truong, T. and Hong, S. and Mitsuhashi, M. and Nyagaka, R. and Lee, S. and Gandhi, A. and De La Torre Schutz, A. and Kesler, S.R. Natural Trajectory Subclasses of Cognitive Impairment in Breast Cancer Patients Experiencing Insomnia. *Cancer* **2025**, *131*, doi:10.1002/cncr.35816.
39. Pang, L.; Bi, Z.; Jing, Y.; Yin, X.; Zhang, X.; Yao, S.; Zhao, J.; Cheng, H. Changes in Cytokine Levels in Breast Cancer Patients with CRCI before or after CALM Intervention. *Am J Cancer Res* **2021**, *11*, 5415–5427.
40. Pang, L. and L., W. and Yao, S. and Jing, Y. and Yin, X. and Cheng, H. Psychological Distress Is Involved in CRCI in Breast Cancer Survivors via Mediating Cytokine Levels. *Cancer Medicine* **2023**, *12*, 11806–11815, doi:10.1002/cam4.5847.
41. Park, J.Y. and L., Cecile A. and Rodriguez, Carmen S. and Meng, Hongdao and Kip, Kevin E. and Morgan, Sandra and Joshi, Anisha and Hueluer, Gizem and Wang, Julia R. and Tinsley, Sara and Cox, Charles and Kiluk, John and Donovan, Kristine A. and Moscoso, Manolete and Bornstein, Elizabeth and Lucas, Jean M. and Fonseca, Tamela and Krothapalli, Mahathi and Padgett, Lynne S. and Nidamanur, Sreenidhi and Hornback, Estella and Patel, Diya and Chamkeri, Ramya and Reich, Richard R. The Moderating Role of Genetics on the Effectiveness of the Mindfulness-Based Stress Reduction for Breast Cancer (MBSR(BC)) Program on Cognitive Impairment. *Biological research for nursing* **2025**, *27*, 216–228, doi:10.1177/10998004241289629.
42. Patel, S.K. and B., E.C. and Paz, I.B. and Kruper, L. and Mortimer, J. and Wong, F.L. and Bhatia, S. and Irwin, M.R. and Behrendt, C.E. Inflammation-Related Proteins as Biomarkers of Treatment-Related Behavioral Symptoms: A Longitudinal Study of Breast Cancer Patients and Age-Matched Controls. *Brain, Behavior, and Immunity - Health* **2023**, *32*, doi:10.1016/j.bbih.2023.100670.
43. Toh, Y.L. and W., C. and Ho, H.K. and Chan, A. Distinct Cytokine Profiles across Trajectories of Self-Perceived Cognitive Impairment among Early-Stage Breast Cancer Survivors. *Journal of Neuroimmunology* **2020**, *342*, doi:10.1016/j.jneuroim.2020.577196.
44. Vardy, J.L.; Stouten-Kemperman, M.M.; Pond, G.; Booth, C.M.; Rourke, S.B.; Dhillon, H.M.; Dodd, A.; Crawley, A.; Tannock, I.F. A Mechanistic Cohort Study Evaluating Cognitive Impairment in Women Treated for Breast Cancer. *Brain Imaging and Behavior* **2019**, *13*, 15–26, doi:10.1007/s11682-017-9728-5.
45. Von Ah, D.; McDonald, B.C.; Crouch, A.D.; Ofner, S.; Perkins, S.; Storey, S.; Considine, R.; Unverzagt, F. Randomized Double-Masked Controlled Trial of Cognitive Training in Breast Cancer Survivors: A Preliminary Study. *Support Care Cancer* **2022**, *30*, 7457–7467, doi:10.1007/s00520-022-07182-4.
46. Yang, G.S. and M., X. and Jackson-Cook, C.K. and Starkweather, A.R. and Lynch Kelly, D. and Archer, K.J. and Zou, F. and Lyon, D.E. Differential DNA Methylation Following Chemotherapy for Breast Cancer Is Associated with Lack of Memory Improvement at One Year. *Epigenetics* **2020**, *15*, 499–510, doi:10.1080/15592294.2019.1699695.
47. Yao, S. and D., K. and Liu, S. and Zhang, Q. and Li, W. and Tang, L. and Yu, S. and Pang, L. and Yin, X. and Cheng, H. The Managing Cancer and Living Meaningfully (CALM) Intervention Alleviates Chemotherapy-Related Cognitive Impairment in Patients with Breast Cancer by Modulating Pan-Immune-Inflammation Values. *Integrative Cancer Therapies* **2022**, *21*, doi:10.1177/15347354221140498.
48. Yao, S. and L., W. and Liu, S. and Cai, Y. and Zhang, Q. and Tang, L. and Yu, S. and Jing, Y. and Yin, X. and Cheng, H. Aldehyde Dehydrogenase 2 Polymorphism Is Associated with Chemotherapy-Related Cognitive Impairment in Patients with Breast Cancer Who Receive Chemotherapy. *Cancer Medicine* **2023**, *12*, 5209–5221, doi:10.1002/cam4.5319.
49. Yap, N.Y.; Tan, N.Y.T.; Tan, C.J.; Loh, K.W.-J.; Ng, R.C.H.; Ho, H.K.; Chan, A. Associations of Plasma Brain-Derived Neurotrophic Factor (BDNF) and Val66Met Polymorphism (Rs6265) with Long-Term

Cancer-Related Cognitive Impairment in Survivors of Breast Cancer. *Breast Cancer Res Treat* **2020**, *183*, 683–696, doi:10.1007/s10549-020-05807-y.
50. Yap, N.Y. and T., Yi Long and Tan, Chia Jie and Acharya, Munjal M. and Chan, Alexandre Relationship between Cytokines and Brain-Derived Neurotrophic Factor (BDNF) in Trajectories of Cancer-Related Cognitive Impairment. *CYTOKINE* **2021**, *144*, doi:10.1016/j.cyto.2021.155556.
51. Yu, S. and Z., J. and Wang, M. and Cheng, G. and Li, W. and Tang, L. and Yao, S. and Pang, L. and Yin, X. and Jing, Y. and Cheng, H. The Correlation between Neutrophil-to-Lymphocyte Ratio, Carcinoembryonic Antigen, and Carbohydrate Antigen 153 Levels with Chemotherapy-Related Cognitive Impairment in Early-Stage Breast Cancer Patients. *Frontiers in Medicine* **2022**, *9*, doi:10.3389/fmed.2022.945433.
52. Zhao, J.; Zuo, H.; Ding, K.; Zhang, X.; Bi, Z.; Cheng, H. Changes in Plasma IL-1β, TNF-α and IL-4 Levels Are Involved in Chemotherapy-Related Cognitive Impairment in Early-Stage Breast Cancer Patients. *American Journal of Translational Research* **2020**, *12*, 3046–3056.
53. Zuniga, K.E. and M., N.E. Low Serum Carotenoids Are Associated with Self-Reported Cognitive Dysfunction and Inflammatory Markers in Breast Cancer Survivors. *Nutrients* **2018**, *10*, doi:10.3390/nu10081111.
